# Supplementary material for: Machine learning reveals distinct T-cell receptor clusters in plasma cell dyscrasias compared to healthy controls
Source: PLoS One. 2025 Oct 27;20(10):e0334053. doi: 10.1371/journal.pone.0334053 (PMC12558469; doi:10.1371/journal.pone.0334053)
Supplement: S1 File — S1 Fig. TCR repertoire downsampling normalizes variation and maintains clonality distribution. A) Box plot comparing the total number of productive rearrangements per sample, categorized by patient diagnosis (n = 612 samples). Wide variability in productive rearrangements is observed across samples and diagnoses, likely reflecting technical variations in sample processing and sequencing depth. Wilcoxon rank-sum test p-values are indicated as follows: *** p < 0.001, ** p < 0.01, * p < 0.05. B) Downsampling of TCRB repertoires reduces the variation in clonality (measured by Gini coefficient) while maintaining the overall distribution across sample types. This normalization step ensures unbiased comparison of TCRB clonality between different groups. Monoclonal gammopathy of undetermined significance (MGUS), smoldering multiple myeloma (SMM), and multiple myeloma (MM). S2 Fig. TCRB repertoire diversity decreases with age. A) Scatter plot comparing age to TCRB clonality (measured by Gini coefficient) in healthy individuals (n = 166) to baseline samples from patients with monoclonal gammopathy of undetermined significance (MGUS, n = 80), smoldering multiple myeloma (SMM, n = 55), and multiple myeloma (MM, n = 31). A moderate positive correlation exists between age and TCRB clonality, particularly evident in healthy and MGUS groups due to their wider age range. B) Subgroup analysis of TCRB repertoire diversity by age and disease state. Healthy individuals aged 40–49 years exhibit significantly greater TCRB diversity compared to MGUS patients of similar age. Within the 60–69 age group, MGUS patients display significantly higher TCRB clonality (lower diversity) than SMM patients. P-values were calculated using the Wilcoxon rank-sum test. *** = 0.001, ** = 0.01, * = 0.05, NS = not significant. S3 Fig. The version of the ImmunoSeq kit does not impact TCRB repertoire diversity. Box plots display the distributions of age, number of productive rearrangements, original TCRB clonal [file pone.0334053.s001.pdf]

SUPPLEMENTAL INFORMATION

Machine learning reveals distinct T-cell receptor clusters in plasma cell dyscrasias compared to healthy controls

David G. Coffey[1*], Yong Zhang[2*], Elizabeth Hill[3], Frank Cross Jr[2], Reena Philip[2,4], Marc R. Theoret[2,4], Ola Landgren[1], Andrea Baines[2], Dickran Kazandjian[1]

Affiliations:
[1]Division of Myeloma, Sylvester Comprehensive Cancer Center, University of Miami, Miami, FL, USA
[2]Center for Drug Evaluation and Research, US Food and Drug Administration, Silver Spring, MD, USA
[3]Lymphoid Malignancies Branch, National Cancer Institute, National Institutes of Health, Bethesda, MD, USA
[4]Oncology Center of Excellence, U.S. Food and Drug Administration, Silver Spring, MD, USA
* These authors contributed equally to this work

SUPPLEMENTAL METHODS

The following R code evaluates the performance of multiple machine learning models in distinguishing healthy individuals from patients with plasma cell dyscrasias (PCD) based on T-cell receptor beta (TCRB) cluster features. In this context, X is a matrix of binary input features, where each row represents a sample and each column corresponds to a differentially abundant TCRB cluster. A value of 1 indicates the presence of that cluster in the sample, and 0 indicates its absence. The response variable y contains the disease status labels (“Healthy” or “PCD”) for each sample, aligned with the rows in X.

To assess model performance and generalizability, the script performs five iterations of stratified train/test splits (80/20), preserving class balance in each split. For each iteration and model type, it trains a classifier, predicts on the test set, computes evaluation metrics, and stores the ROC curve. The results for each model and iteration are compiled into a single data frame, which is shown in Supplemental Table 4.

```
library(dplyr)
library(tibble)
library(caret)
library(pROC)

# Assumes X (data frame) and y (factor) are already imported

# Set up repeated stratified train/test splits
n.repeats = 5
test.size = 0.20

# Initialize variables
results = list()
roc_list = list()
model_names = c("rf", "svmRadial", "glmnet", "nnet", "gbm", "knn")
models = list()

# Loop over model types
for (model.name in model_names) {
  metrics = data.frame()
  roc_curves = list()

  for (i in 1:n.repeats) {
    train.index = createDataPartition(y, p = 1 - test.size, list = FALSE)
    X.train = X[train.index, ]
    X.test = X[-train.index, ]
    y.train = y[train.index]
    y.test = y[-train.index]

    ctrl = trainControl(method = "none", classProbs = TRUE, summaryFunction = twoClassSummary)
    model = train(X.train, y.train, method = model.name, metric = "ROC", trControl = ctrl)

    prob = predict(model, X.test, type = "prob")
    pred = predict(model, X.test)

    roc.obj = roc(y.test, prob$Myeloma)
    roc_curves[[i]] = roc.obj

    cm = confusionMatrix(pred, y.test, positive = "Myeloma")
    acc = cm$overall["Accuracy"]
    kappa = cm$overall["Kappa"]
```

```
    sens = cm$byClass["Sensitivity"]
    spec = cm$byClass["Specificity"]
    auc = as.numeric(auc(roc.obj))

    metrics = rbind(metrics, data.frame(
      Repeat = i,
      Accuracy = as.numeric(acc),
      Kappa = as.numeric(kappa),
      Sensitivity = as.numeric(sens),
      Specificity = as.numeric(spec),
      AUROC = auc
    ))
  }

  results[[model.name]] = metrics
  roc_list[[model.name]] = roc_curves
  models[[model.name]] = model
}

# Combine and calculate 95% CI
combined_results = bind_rows(lapply(names(results), function(m) {
  mutate(results[[m]], Model = m)
}))
```

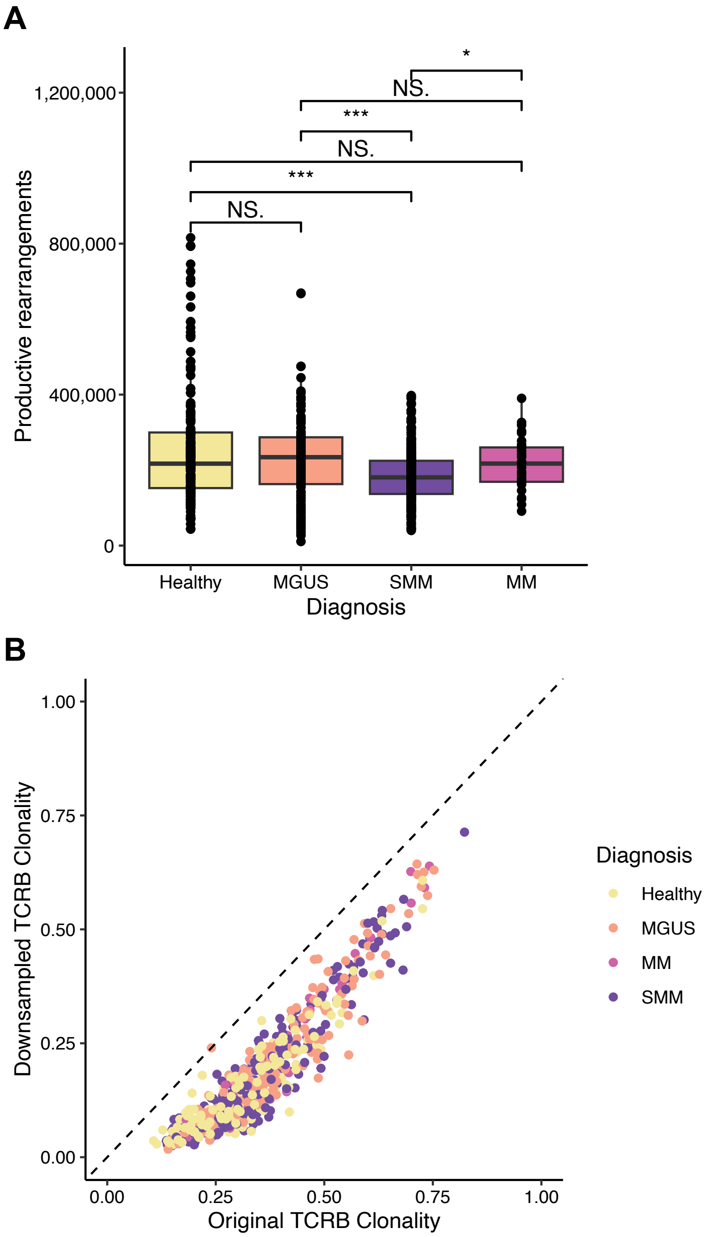

S1 Figure. TCR repertoire downsampling normalizes variation and maintains clonality distribution. A) Box plot comparing the total number of productive rearrangements per sample, categorized by patient diagnosis (n = 612 samples). Wide variability in productive rearrangements is observed across samples and diagnoses, likely reflecting technical variations in sample processing and sequencing depth. Wilcoxon rank-sum test p-values are indicated as follows: *** $p < 0.001$, ** $p < 0.01$, * $p < 0.05$. B) Downsampling of TCRB repertoires reduces the variation in clonality (measured by Gini coefficient) while maintaining the overall distribution across sample types. This normalization step ensures unbiased comparison of TCRB clonality between different groups. Monoclonal gammopathy of undetermined significance (MGUS), smoldering multiple myeloma (SMM), and multiple myeloma (MM).

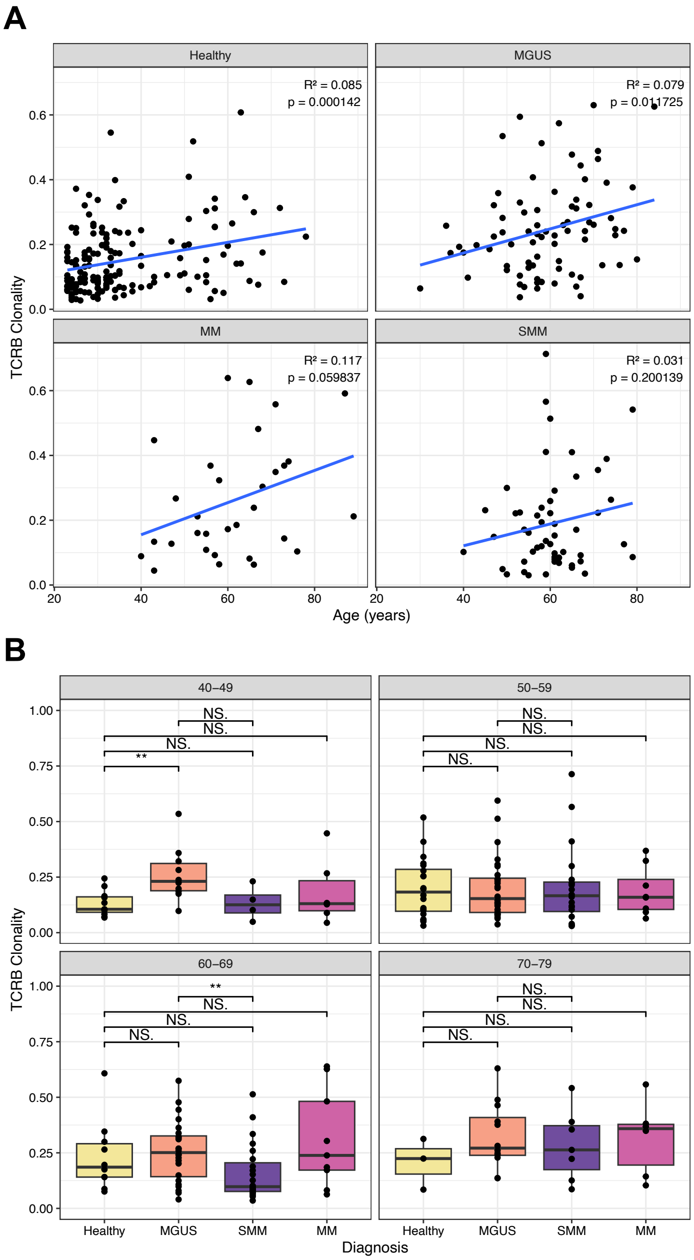

S2 Figure. TCRB repertoire diversity decreases with age. A) Scatter plot comparing age to TCRB clonality (measured by Gini coefficient) in healthy individuals (n = 166) to baseline samples from patients with monoclonal gammopathy of undetermined significance (MGUS, n = 80), smoldering multiple myeloma (SMM, n = 55), and multiple myeloma (MM, n = 31). A moderate positive correlation exists between age and TCRB clonality, particularly evident in healthy and MGUS groups due to their wider age range. B) Subgroup analysis of TCRB repertoire diversity by age and disease state. Healthy individuals aged 40-49 years exhibit significantly greater TCRB diversity compared to MGUS patients of similar age. Within the 60-69 age group, MGUS patients display significantly higher TCRB clonality (lower diversity) than SMM patients. P-values were calculated using the Wilcoxon rank-sum test. *** = 0.001, ** = 0.01, * = 0.05, NS = not significant.

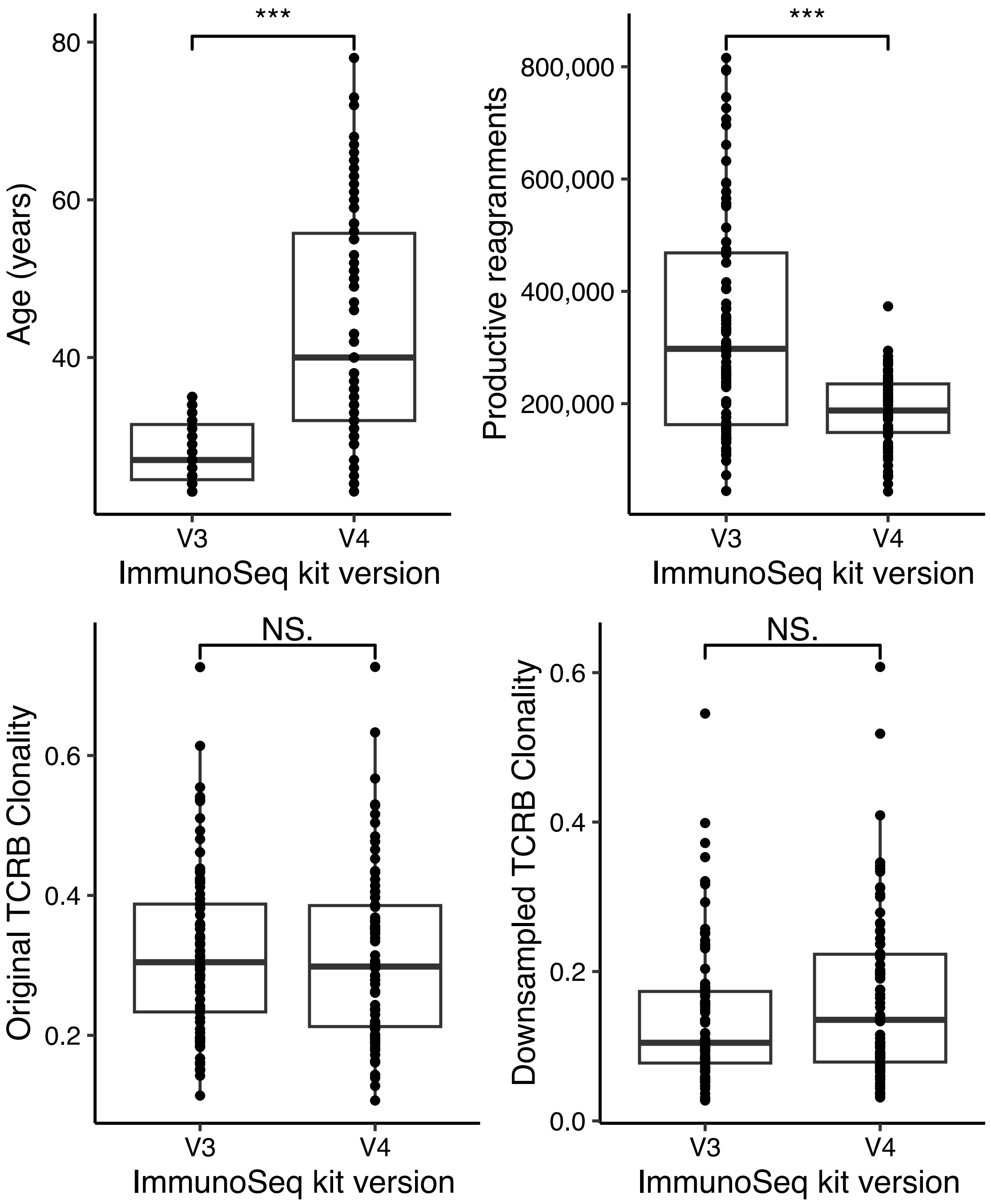

S3 Figure. The version of the ImmunoSeq kit does not impact TCRB repertoire diversity. Box plots display the distributions of age, number of productive rearrangements, original TCRB clonality, and downsampled TCRB clonality across individuals sequenced with ImmunoSeq version 3 (V3, n = 84) and version 4 (V4, n = 82) among healthy donors. P-values were calculated using the Wilcoxon rank-sum test. *** = 0.001, ** = 0.01, * = 0.05, NS = not significant.

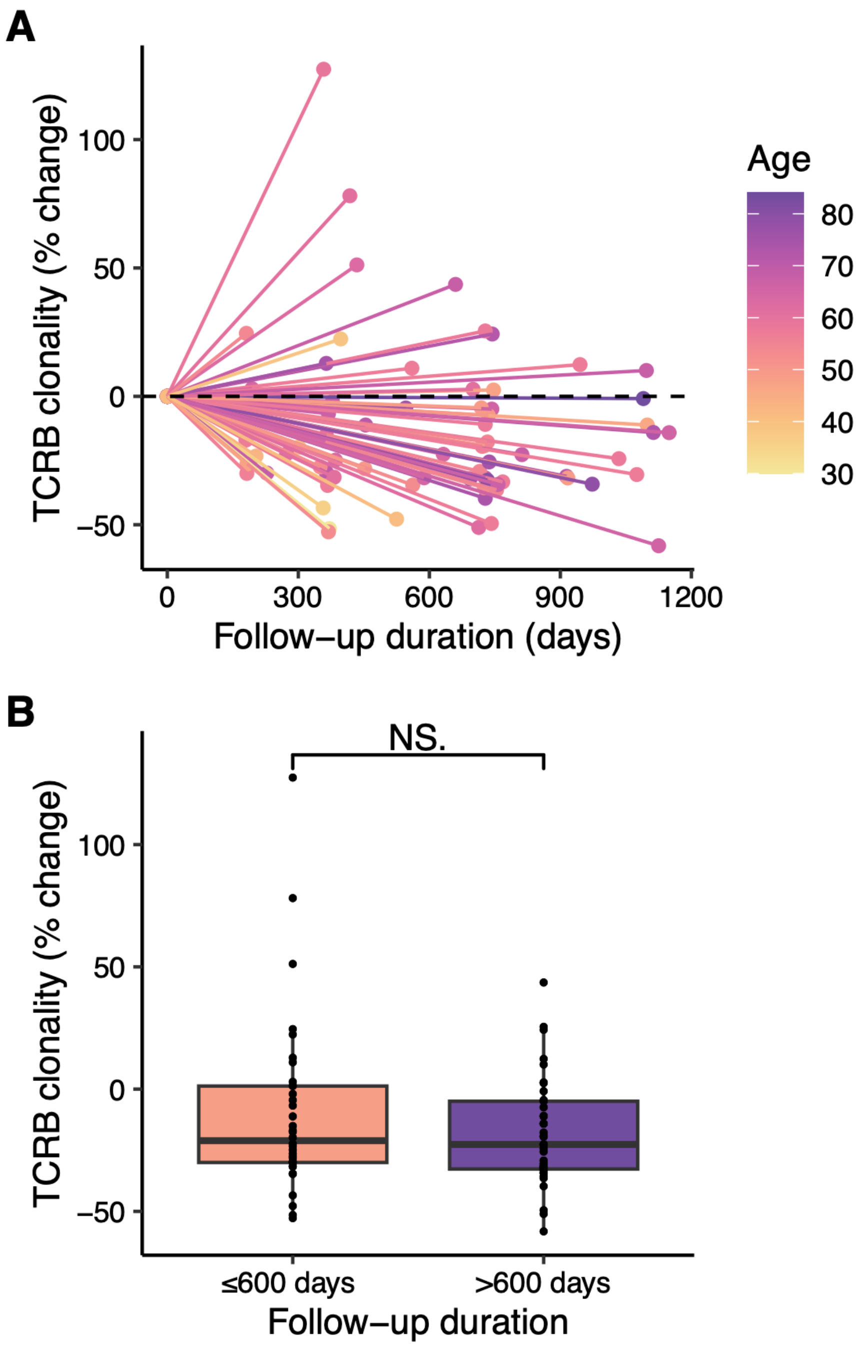

S4 Figure. Longitudinal stability of the T cell receptor beta (TCRB) repertoire in patients with monoclonal gammopathy of undetermined significance (MGUS). A) Percent change in TCRB clonality between two time points for 72 MGUS patients with at least two samples collected over time. Each line represents an individual patient. While a trend toward increased TCRB diversity over time is observed for the majority of patients, B) a paired Wilcoxon rank-sign test reveals no statistically significant difference in clonality changes within the first 600 days compared to later time points. NS = not significant.

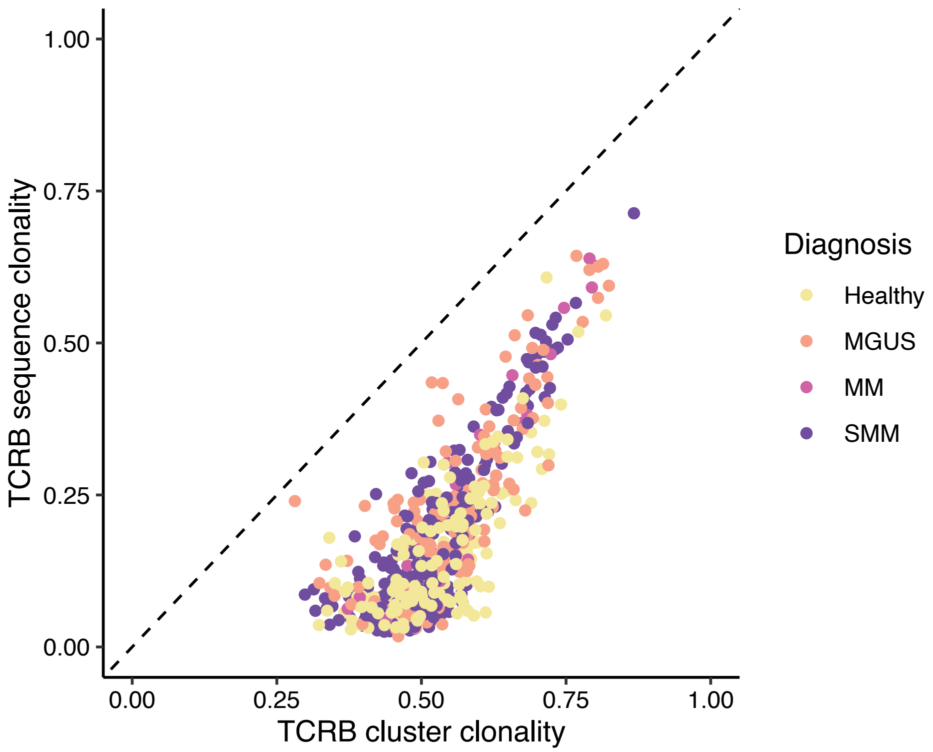

S5 Figure. Correlation between TCRB CDR3β amino acid sequence diversity and TCRB cluster diversity. The Gini coefficient, a measure of inequality, was calculated for the frequency of TCRB CDR3β amino acid sequences and the frequency of TCRB clusters (identified using the ClusTCR method). A higher Gini coefficient indicates greater diversity in the TCR repertoire. Spearman correlation 0.762.

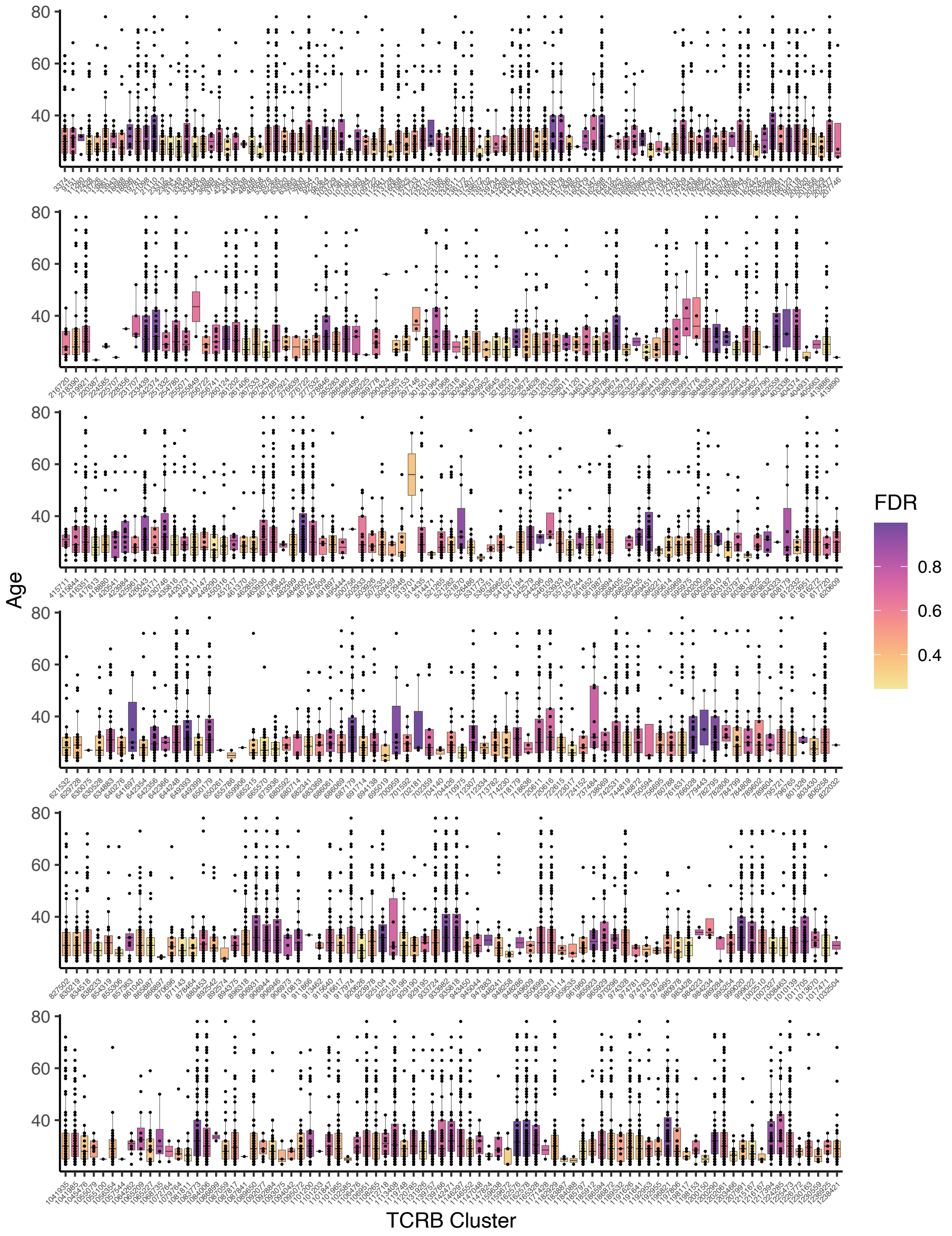

S6 Figure. Age distribution of healthy individuals across all differentially abundant clusters used in the training dataset. To assess potential age bias in the TCRB cluster composition, we compared the age distribution of healthy individuals within each differentially abundant cluster to the age distribution of all healthy individuals (n=166) in the training dataset. Using a Wilcoxon Rank Sum test with FDR correction, we found no significant differences in age distributions between any cluster and the overall healthy population. This indicates that the identified TCRB clusters are not driven by age-specific variations.
