# Supplementary material for: Machine learning reveals distinct T-cell receptor clusters in plasma cell dyscrasias compared to healthy controls
Source: PLoS One. 2025 Oct 27;20(10):e0334053. doi: 10.1371/journal.pone.0334053 (PMC12558469; doi:10.1371/journal.pone.0334053)
Supplement: S1 Table — Minimal residual disease (MRD) assessment is shown for smolder multiple myeloma (SMM) samples after cycles 1, 4, 8, 20, and 32 of carfilzomib, lenalidomide, and dexamethasone (KRd). Deepest response assessed by the International Myeloma Working Group following induction therapy for multiple myeloma (MM) is shown. Monoclonal gammopathy of undetermined signifiance (MGUS) samples are from diagnose and follow-up of untreated patients. Healthy samples were obtained from two pubicaly available datasets. sCR, stringent complete response; CR, complete response; VGPR, very good partial response; PR, partial response. (PDF) [file pone.0334053.s002.pdf]

S1 Table. Individual sample characteristics (n = 612). Minimal residual disease (MRD) assessment is shown for smolder multiple myeloma (SMM) samples after cycles 1, 4, 8, 20, and 32 of carfilzomib, lenalidomide, and dexamethasone (KRd). Deepest response assessed by the International Myeloma Working Group following induction therapy for multiple myeloma (MM) is shown. Monoclonal gammopathy of undetermined signifiance (MGUS) samples are from diagnose and follow-up of untreated patients. Healthy samples

Sample	Patient	Time point	Diagnosis	Race	ImmunoSeq version	Tissue	KRd Cycle 8	KRd Cycle 20	KRd Cycle 32	Induction best response	Source of healthy data	Follow-up days for MGUS
Keck0003_MC1	Keck0003_MC1	Baseline	Healthy	Native American	V3	PBMC					https://doi.org/10.21417/B7001Z	
Keck0004_MC1	Keck0004_MC1	Baseline	Healthy	Caucasian	V3	PBMC					https://doi.org/10.21417/B7001Z	
Keck0005_MC1	Keck0005_MC1	Baseline	Healthy	Caucasian	V3	PBMC					https://doi.org/10.21417/B7001Z	
Keck0006_MC1	Keck0006_MC1	Baseline	Healthy	Caucasian	V3	PBMC					https://doi.org/10.21417/B7001Z	
Keck0008_MC1	Keck0008_MC1	Baseline	Healthy	Caucasian	V3	PBMC					https://doi.org/10.21417/B7001Z	
Keck0010_MC1	Keck0010_MC1	Baseline	Healthy	Caucasian	V3	PBMC					https://doi.org/10.21417/B7001Z	
Keck0011_MC1	Keck0011_MC1	Baseline	Healthy	Caucasian	V3	PBMC					https://doi.org/10.21417/B7001Z	
Keck0012_MC1	Keck0012_MC1	Baseline	Healthy	Asian	V3	PBMC					https://doi.org/10.21417/B7001Z	
Keck0013_MC1	Keck0013_MC1	Baseline	Healthy	Caucasian	V3	PBMC					https://doi.org/10.21417/B7001Z	
Keck0021_MC1	Keck0021_MC1	Baseline	Healthy	Caucasian	V3	PBMC					https://doi.org/10.21417/B7001Z	
Keck0024_MC1	Keck0024_MC1	Baseline	Healthy	Caucasian	V3	PBMC					https://doi.org/10.21417/B7001Z	
Keck0027_MC1	Keck0027_MC1	Baseline	Healthy	Caucasian	V3	PBMC					https://doi.org/10.21417/B7001Z	
Keck0030_MC1	Keck0030_MC1	Baseline	Healthy	Caucasian	V3	PBMC					https://doi.org/10.21417/B7001Z	
Keck0031_MC1	Keck0031_MC1	Baseline	Healthy	Caucasian	V3	PBMC					https://doi.org/10.21417/B7001Z	
Keck0033_MC1	Keck0033_MC1	Baseline	Healthy	Asian	V3	PBMC					https://doi.org/10.21417/B7001Z	
Keck0036_MC1	Keck0036_MC1	Baseline	Healthy	Caucasian	V3	PBMC					https://doi.org/10.21417/B7001Z	
Keck0037_MC1	Keck0037_MC1	Baseline	Healthy	Caucasian	V3	PBMC					https://doi.org/10.21417/B7001Z	
Keck0039_MC1	Keck0039_MC1	Baseline	Healthy	Caucasian	V3	PBMC					https://doi.org/10.21417/B7001Z	
Keck0040_MC1	Keck0040_MC1	Baseline	Healthy	Caucasian	V3	PBMC					https://doi.org/10.21417/B7001Z	
Keck0041_MC1	Keck0041_MC1	Baseline	Healthy	Caucasian	V3	PBMC					https://doi.org/10.21417/B7001Z	
Keck0044_MC1	Keck0044_MC1	Baseline	Healthy	Asian	V3	PBMC					https://doi.org/10.21417/B7001Z	
Keck0045_MC1	Keck0045_MC1	Baseline	Healthy	Caucasian	V3	PBMC					https://doi.org/10.21417/B7001Z	
Keck0046_MC1	Keck0046_MC1	Baseline	Healthy	Caucasian	V3	PBMC					https://doi.org/10.21417/B7001Z	
Keck0047_MC1	Keck0047_MC1	Baseline	Healthy	Caucasian	V3	PBMC					https://doi.org/10.21417/B7001Z	
Keck0048_MC1	Keck0048_MC1	Baseline	Healthy	Caucasian	V3	PBMC					https://doi.org/10.21417/B7001Z	
Keck0049_MC1	Keck0049_MC1	Baseline	Healthy	Asian	V3	PBMC					https://doi.org/10.21417/B7001Z	
Keck0050_MC1	Keck0050_MC1	Baseline	Healthy		V3	PBMC					https://doi.org/10.21417/B7001Z	
Keck0051_MC1	Keck0051_MC1	Baseline	Healthy	Caucasian	V3	PBMC					https://doi.org/10.21417/B7001Z	
Keck0052_MC1	Keck0052_MC1	Baseline	Healthy	Caucasian	V3	PBMC					https://doi.org/10.21417/B7001Z	
Keck0053_MC1	Keck0053_MC1	Baseline	Healthy	Caucasian	V3	PBMC					https://doi.org/10.21417/B7001Z	
Keck0054_MC1	Keck0054_MC1	Baseline	Healthy		V3	PBMC					https://doi.org/10.21417/B7001Z	
Keck0055_MC1	Keck0055_MC1	Baseline	Healthy	Caucasian	V3	PBMC					https://doi.org/10.21417/B7001Z	
Keck0056_MC1	Keck0056_MC1	Baseline	Healthy	Caucasian	V3	PBMC					https://doi.org/10.21417/B7001Z	
Keck0057_MC1	Keck0057_MC1	Baseline	Healthy	Caucasian	V3	PBMC					https://doi.org/10.21417/B7001Z	
Keck0058_MC1	Keck0058_MC1	Baseline	Healthy	Asian	V3	PBMC					https://doi.org/10.21417/B7001Z	
Keck0059_MC1	Keck0059_MC1	Baseline	Healthy	Caucasian	V3	PBMC					https://doi.org/10.21417/B7001Z	
Keck0062_MC1	Keck0062_MC1	Baseline	Healthy	Asian	V3	PBMC					https://doi.org/10.21417/B7001Z	
Keck0063_MC1	Keck0063_MC1	Baseline	Healthy	Caucasian	V3	PBMC					https://doi.org/10.21417/B7001Z	
Keck0064_MC1	Keck0064_MC1	Baseline	Healthy	Caucasian	V3	PBMC					https://doi.org/10.21417/B7001Z	
Keck0065_MC1	Keck0065_MC1	Baseline	Healthy	Asian	V3	PBMC					https://doi.org/10.21417/B7001Z	
Keck0066_MC1	Keck0066_MC1	Baseline	Healthy	Caucasian	V3	PBMC					https://doi.org/10.21417/B7001Z	
Keck0067_MC1	Keck0067_MC1	Baseline	Healthy	Asian	V3	PBMC					https://doi.org/10.21417/B7001Z	
Keck0068_MC1	Keck0068_MC1	Baseline	Healthy	Caucasian	V3	PBMC					https://doi.org/10.21417/B7001Z	
Keck0069_MC1	Keck0069_MC1	Baseline	Healthy	Caucasian	V3	PBMC					https://doi.org/10.21417/B7001Z	
Keck0070_MC1	Keck0070_MC1	Baseline	Healthy	Caucasian	V3	PBMC					https://doi.org/10.21417/B7001Z	
Keck0071_MC1	Keck0071_MC1	Baseline	Healthy	Caucasian	V3	PBMC					https://doi.org/10.21417/B7001Z	
Keck0072_MC1	Keck0072_MC1	Baseline	Healthy	Caucasian	V3	PBMC					https://doi.org/10.21417/B7001Z	
Keck0073_MC1	Keck0073_MC1	Baseline	Healthy	Caucasian	V3	PBMC					https://doi.org/10.21417/B7001Z	
Keck0074_MC1	Keck0074_MC1	Baseline	Healthy	Caucasian	V3	PBMC					https://doi.org/10.21417/B7001Z	
Keck0075_MC1	Keck0075_MC1	Baseline	Healthy	Asian	V3	PBMC					https://doi.org/10.21417/B7001Z	
Keck0076_MC1	Keck0076_MC1	Baseline	Healthy	Asian	V3	PBMC					https://doi.org/10.21417/B7001Z	
Keck0077_MC1	Keck0077_MC1	Baseline	Healthy	Caucasian	V3	PBMC					https://doi.org/10.21417/B7001Z	
Keck0078_MC1	Keck0078_MC1	Baseline	Healthy	Caucasian	V3	PBMC					https://doi.org/10.21417/B7001Z	
Keck0079_MC1	Keck0079_MC1	Baseline	Healthy	Caucasian	V3	PBMC					https://doi.org/10.21417/B7001Z	
Keck0080_MC1	Keck0080_MC1	Baseline	Healthy	Caucasian	V3	PBMC					https://doi.org/10.21417/B7001Z	
Keck0081_MC1	Keck0081_MC1	Baseline	Healthy	Caucasian	V3	PBMC					https://doi.org/10.21417/B7001Z	
Keck0082_MC1	Keck0082_MC1	Baseline	Healthy	Caucasian	V3	PBMC					https://doi.org/10.21417/B7001Z	
Keck0083_MC1	Keck0083_MC1	Baseline	Healthy	Caucasian	V3	PBMC					https://doi.org/10.21417/B7001Z	
Keck0084_MC1	Keck0084_MC1	Baseline	Healthy	Asian	V3	PBMC					https://doi.org/10.21417/B7001Z	
Keck0085_MC1	Keck0085_MC1	Baseline	Healthy	Caucasian	V3	PBMC					https://doi.org/10.21417/B7001Z	
Keck0086_MC1	Keck0086_MC1	Baseline	Healthy	Caucasian	V3	PBMC					https://doi.org/10.21417/B7001Z	
Keck0087_MC1	Keck0087_MC1	Baseline	Healthy	Caucasian	V3	PBMC					https://doi.org/10.21417/B7001Z	
Keck0088_MC1	Keck0088_MC1	Baseline	Healthy	Asian	V3	PBMC					https://doi.org/10.21417/B7001Z	

S1 Table. Individual sample characteristics (n = 612). Minimal residual disease (MRD) assessment is shown for smolder multiple myeloma (SMM) samples after cycles 1, 4, 8, 20, and 32 of carfilzomib, lenalidomide, and dexamethasone (KRd). Deepest response assessed by the International Myeloma Working Group following induction therapy for multiple myeloma (MM) is shown. Monoclonal gammopathy of undetermined signifiance (MGUS) samples are from diagnose and follow-up of untreated patients. Healthy samples

Sample	Patient	Time point	Diagnosis	Race	ImmunoSeq version	Tissue	KRd Cycle 8	KRd Cycle 20	KRd Cycle 32	Induction best response	Source of healthy data	Follow-up days for MGUS
Keck0089_MC1	Keck0089_MC1	Baseline	Healthy	Black or African American	V3	PBMC					https://doi.org/10.21417/B7001Z	
Keck0091_MC1	Keck0091_MC1	Baseline	Healthy	Black or African American	V3	PBMC					https://doi.org/10.21417/B7001Z	
Keck0093_MC1	Keck0093_MC1	Baseline	Healthy	Caucasian	V3	PBMC					https://doi.org/10.21417/B7001Z	
Keck0094_MC1	Keck0094_MC1	Baseline	Healthy	Caucasian	V3	PBMC					https://doi.org/10.21417/B7001Z	
Keck0095_MC1	Keck0095_MC1	Baseline	Healthy	Caucasian	V3	PBMC					https://doi.org/10.21417/B7001Z	
Keck0096_MC1	Keck0096_MC1	Baseline	Healthy	Caucasian	V3	PBMC					https://doi.org/10.21417/B7001Z	
Keck0101_MC1	Keck0101_MC1	Baseline	Healthy	Caucasian	V3	PBMC					https://doi.org/10.21417/B7001Z	
Keck0102_MC1	Keck0102_MC1	Baseline	Healthy	Caucasian	V3	PBMC					https://doi.org/10.21417/B7001Z	
Keck0103_MC1	Keck0103_MC1	Baseline	Healthy	Caucasian	V3	PBMC					https://doi.org/10.21417/B7001Z	
Keck0104_MC1	Keck0104_MC1	Baseline	Healthy	Caucasian	V3	PBMC					https://doi.org/10.21417/B7001Z	
Keck0105_MC1	Keck0105_MC1	Baseline	Healthy	Caucasian	V3	PBMC					https://doi.org/10.21417/B7001Z	
Keck0106_MC1	Keck0106_MC1	Baseline	Healthy	Caucasian	V3	PBMC					https://doi.org/10.21417/B7001Z	
Keck0108_MC1	Keck0108_MC1	Baseline	Healthy		V3	PBMC					https://doi.org/10.21417/B7001Z	
Keck0109_MC1	Keck0109_MC1	Baseline	Healthy	Caucasian	V3	PBMC					https://doi.org/10.21417/B7001Z	
Keck0110_MC1	Keck0110_MC1	Baseline	Healthy	Caucasian	V3	PBMC					https://doi.org/10.21417/B7001Z	
Keck0112_MC1	Keck0112_MC1	Baseline	Healthy	Caucasian	V3	PBMC					https://doi.org/10.21417/B7001Z	
Keck0115_MC1	Keck0115_MC1	Baseline	Healthy	Caucasian	V3	PBMC					https://doi.org/10.21417/B7001Z	
Keck0117_MC1	Keck0117_MC1	Baseline	Healthy	Caucasian	V3	PBMC					https://doi.org/10.21417/B7001Z	
Keck0118_MC1	Keck0118_MC1	Baseline	Healthy	Caucasian	V3	PBMC					https://doi.org/10.21417/B7001Z	
Keck0119_MC1	Keck0119_MC1	Baseline	Healthy	Asian	V3	PBMC					https://doi.org/10.21417/B7001Z	
Keck0120_MC1	Keck0120_MC1	Baseline	Healthy	Caucasian	V3	PBMC					https://doi.org/10.21417/B7001Z	
Subject_100	Subject_100	Baseline	Healthy	Black or African American	V4	Blood					https://doi.org/10.21417/ADPT2020V4CD	
Subject_101	Subject_101	Baseline	Healthy	Black or African American	V4	Blood					https://doi.org/10.21417/ADPT2020V4CD	
Subject_102	Subject_102	Baseline	Healthy	Black or African American	V4	Blood					https://doi.org/10.21417/ADPT2020V4CD	
Subject_103	Subject_103	Baseline	Healthy	Black or African American	V4	Blood					https://doi.org/10.21417/ADPT2020V4CD	
Subject_104	Subject_104	Baseline	Healthy	Black or African American	V4	Blood					https://doi.org/10.21417/ADPT2020V4CD	
Subject_105	Subject_105	Baseline	Healthy	Black or African American	V4	Blood					https://doi.org/10.21417/ADPT2020V4CD	
Subject_106	Subject_106	Baseline	Healthy	Black or African American	V4	Blood					https://doi.org/10.21417/ADPT2020V4CD	
Subject_12	Subject_12	Baseline	Healthy	Asian or Pacific Islander	V4	Blood					https://doi.org/10.21417/ADPT2020V4CD	
Subject_13	Subject_13	Baseline	Healthy	Asian or Pacific Islander	V4	Blood					https://doi.org/10.21417/ADPT2020V4CD	
Subject_14	Subject_14	Baseline	Healthy	Asian or Pacific Islander	V4	Blood					https://doi.org/10.21417/ADPT2020V4CD	
Subject_15	Subject_15	Baseline	Healthy	Asian or Pacific Islander	V4	Blood					https://doi.org/10.21417/ADPT2020V4CD	
Subject_151	Subject_151	Baseline	Healthy	Caucasian	V4	Blood					https://doi.org/10.21417/ADPT2020V4CD	
Subject_152	Subject_152	Baseline	Healthy	Caucasian	V4	Blood					https://doi.org/10.21417/ADPT2020V4CD	
Subject_153	Subject_153	Baseline	Healthy		V4	Blood					https://doi.org/10.21417/ADPT2020V4CD	
Subject_154	Subject_154	Baseline	Healthy	Caucasian	V4	Blood					https://doi.org/10.21417/ADPT2020V4CD	
Subject_155	Subject_155	Baseline	Healthy	Asian or Pacific Islander	V4	Blood					https://doi.org/10.21417/ADPT2020V4CD	
Subject_156	Subject_156	Baseline	Healthy	Asian or Pacific Islander	V4	Blood					https://doi.org/10.21417/ADPT2020V4CD	
Subject_157	Subject_157	Baseline	Healthy	Caucasian	V4	Blood					https://doi.org/10.21417/ADPT2020V4CD	
Subject_158	Subject_158	Baseline	Healthy	Hispanic	V4	Blood					https://doi.org/10.21417/ADPT2020V4CD	
Subject_16	Subject_16	Baseline	Healthy	Asian or Pacific Islander	V4	Blood					https://doi.org/10.21417/ADPT2020V4CD	
Subject_18	Subject_18	Baseline	Healthy	Asian or Pacific Islander	V4	Blood					https://doi.org/10.21417/ADPT2020V4CD	
Subject_22	Subject_22	Baseline	Healthy	Asian or Pacific Islander	V4	Blood					https://doi.org/10.21417/ADPT2020V4CD	
Subject_24	Subject_24	Baseline	Healthy	Asian or Pacific Islander	V4	Blood					https://doi.org/10.21417/ADPT2020V4CD	
Subject_26	Subject_26	Baseline	Healthy	Asian or Pacific Islander	V4	Blood					https://doi.org/10.21417/ADPT2020V4CD	
Subject_27	Subject_27	Baseline	Healthy	Asian or Pacific Islander	V4	Blood					https://doi.org/10.21417/ADPT2020V4CD	
Subject_39	Subject_39	Baseline	Healthy	Asian or Pacific Islander	V4	Blood					https://doi.org/10.21417/ADPT2020V4CD	
Subject_40	Subject_40	Baseline	Healthy	Asian or Pacific Islander	V4	Blood					https://doi.org/10.21417/ADPT2020V4CD	
Subject_41	Subject_41	Baseline	Healthy	Caucasian	V4	Blood					https://doi.org/10.21417/ADPT2020V4CD	
Subject_42	Subject_42	Baseline	Healthy	Caucasian	V4	Blood					https://doi.org/10.21417/ADPT2020V4CD	
Subject_44	Subject_44	Baseline	Healthy	Caucasian	V4	Blood					https://doi.org/10.21417/ADPT2020V4CD	
Subject_45	Subject_45	Baseline	Healthy	Caucasian	V4	Blood					https://doi.org/10.21417/ADPT2020V4CD	
Subject_46	Subject_46	Baseline	Healthy	Caucasian	V4	Blood					https://doi.org/10.21417/ADPT2020V4CD	
Subject_47	Subject_47	Baseline	Healthy	Caucasian	V4	Blood					https://doi.org/10.21417/ADPT2020V4CD	
Subject_48	Subject_48	Baseline	Healthy	Hispanic	V4	Blood					https://doi.org/10.21417/ADPT2020V4CD	
Subject_49	Subject_49	Baseline	Healthy	Caucasian	V4	Blood					https://doi.org/10.21417/ADPT2020V4CD	
Subject_50	Subject_50	Baseline	Healthy	Caucasian	V4	Blood					https://doi.org/10.21417/ADPT2020V4CD	
Subject_51	Subject_51	Baseline	Healthy	Caucasian	V4	Blood					https://doi.org/10.21417/ADPT2020V4CD	
Subject_52	Subject_52	Baseline	Healthy	Hispanic	V4	Blood					https://doi.org/10.21417/ADPT2020V4CD	
Subject_53	Subject_53	Baseline	Healthy	Caucasian	V4	Blood					https://doi.org/10.21417/ADPT2020V4CD	
Subject_54	Subject_54	Baseline	Healthy	Caucasian	V4	Blood					https://doi.org/10.21417/ADPT2020V4CD	
Subject_55	Subject_55	Baseline	Healthy	Caucasian	V4	Blood					https://doi.org/10.21417/ADPT2020V4CD	
Subject_56	Subject_56	Baseline	Healthy	Caucasian	V4	Blood					https://doi.org/10.21417/ADPT2020V4CD	

S1 Table. Individual sample characteristics (n = 612). Minimal residual disease (MRD) assessment is shown for smolder multiple myeloma (SMM) samples after cycles 1, 4, 8, 20, and 32 of carfilzomib, lenalidomide, and dexamethasone (KRd). Deepest response assessed by the International Myeloma Working Group following induction therapy for multiple myeloma (MM) is shown. Monoclonal gammopathy of undetermined signifiance (MGUS) samples are from diagnose and follow-up of untreated patients. Healthy samples

Sample	Patient	Time point	Diagnosis	Race	ImmunoSeq version	Tissue	KRd Cycle 8	KRd Cycle 20	KRd Cycle 32	Induction best response	Source of healthy data	Follow-up days for MGUS
Subject_57	Subject_57	Baseline	Healthy	Caucasian	V4	Blood					https://doi.org/10.21417/ADPT2020V4CD	
Subject_58	Subject_58	Baseline	Healthy	Caucasian	V4	Blood					https://doi.org/10.21417/ADPT2020V4CD	
Subject_59	Subject_59	Baseline	Healthy	Caucasian	V4	Blood					https://doi.org/10.21417/ADPT2020V4CD	
Subject_60	Subject_60	Baseline	Healthy	Caucasian	V4	Blood					https://doi.org/10.21417/ADPT2020V4CD	
Subject_62	Subject_62	Baseline	Healthy	Hispanic	V4	Blood					https://doi.org/10.21417/ADPT2020V4CD	
Subject_63	Subject_63	Baseline	Healthy	Hispanic	V4	Blood					https://doi.org/10.21417/ADPT2020V4CD	
Subject_64	Subject_64	Baseline	Healthy	Hispanic	V4	Blood					https://doi.org/10.21417/ADPT2020V4CD	
Subject_65	Subject_65	Baseline	Healthy	Hispanic	V4	Blood					https://doi.org/10.21417/ADPT2020V4CD	
Subject_66	Subject_66	Baseline	Healthy	Hispanic	V4	Blood					https://doi.org/10.21417/ADPT2020V4CD	
Subject_67	Subject_67	Baseline	Healthy	Hispanic	V4	Blood					https://doi.org/10.21417/ADPT2020V4CD	
Subject_68	Subject_68	Baseline	Healthy	Hispanic	V4	Blood					https://doi.org/10.21417/ADPT2020V4CD	
Subject_69	Subject_69	Baseline	Healthy	Hispanic	V4	Blood					https://doi.org/10.21417/ADPT2020V4CD	
Subject_70	Subject_70	Baseline	Healthy	Hispanic	V4	Blood					https://doi.org/10.21417/ADPT2020V4CD	
Subject_71	Subject_71	Baseline	Healthy	Hispanic	V4	Blood					https://doi.org/10.21417/ADPT2020V4CD	
Subject_72	Subject_72	Baseline	Healthy	Hispanic	V4	Blood					https://doi.org/10.21417/ADPT2020V4CD	
Subject_73	Subject_73	Baseline	Healthy	Hispanic	V4	Blood					https://doi.org/10.21417/ADPT2020V4CD	
Subject_74	Subject_74	Baseline	Healthy	Hispanic	V4	Blood					https://doi.org/10.21417/ADPT2020V4CD	
Subject_75	Subject_75	Baseline	Healthy	Hispanic	V4	Blood					https://doi.org/10.21417/ADPT2020V4CD	
Subject_76	Subject_76	Baseline	Healthy	Hispanic	V4	Blood					https://doi.org/10.21417/ADPT2020V4CD	
Subject_77	Subject_77	Baseline	Healthy	Hispanic	V4	Blood					https://doi.org/10.21417/ADPT2020V4CD	
Subject_79	Subject_79	Baseline	Healthy	Hispanic	V4	Blood					https://doi.org/10.21417/ADPT2020V4CD	
Subject_80	Subject_80	Baseline	Healthy	Caucasian	V4	Blood					https://doi.org/10.21417/ADPT2020V4CD	
Subject_81	Subject_81	Baseline	Healthy	Caucasian	V4	Blood					https://doi.org/10.21417/ADPT2020V4CD	
Subject_82	Subject_82	Baseline	Healthy	Black or African American	V4	Blood					https://doi.org/10.21417/ADPT2020V4CD	
Subject_83	Subject_83	Baseline	Healthy	Black or African American	V4	Blood					https://doi.org/10.21417/ADPT2020V4CD	
Subject_84	Subject_84	Baseline	Healthy	Black or African American	V4	Blood					https://doi.org/10.21417/ADPT2020V4CD	
Subject_85	Subject_85	Baseline	Healthy	Black or African American	V4	Blood					https://doi.org/10.21417/ADPT2020V4CD	
Subject_86	Subject_86	Baseline	Healthy	Black or African American	V4	Blood					https://doi.org/10.21417/ADPT2020V4CD	
Subject_88	Subject_88	Baseline	Healthy	Black or African American	V4	Blood					https://doi.org/10.21417/ADPT2020V4CD	
Subject_89	Subject_89	Baseline	Healthy	Black or African American	V4	Blood					https://doi.org/10.21417/ADPT2020V4CD	
Subject_90	Subject_90	Baseline	Healthy	Black or African American	V4	Blood					https://doi.org/10.21417/ADPT2020V4CD	
Subject_91	Subject_91	Baseline	Healthy	Black or African American	V4	Blood					https://doi.org/10.21417/ADPT2020V4CD	
Subject_92	Subject_92	Baseline	Healthy	Black or African American	V4	Blood					https://doi.org/10.21417/ADPT2020V4CD	
Subject_93	Subject_93	Baseline	Healthy	Black or African American	V4	Blood					https://doi.org/10.21417/ADPT2020V4CD	
Subject_94	Subject_94	Baseline	Healthy	Black or African American	V4	Blood					https://doi.org/10.21417/ADPT2020V4CD	
Subject_95	Subject_95	Baseline	Healthy	Black or African American	V4	Blood					https://doi.org/10.21417/ADPT2020V4CD	
Subject_96	Subject_96	Baseline	Healthy	Black or African American	V4	Blood					https://doi.org/10.21417/ADPT2020V4CD	
Subject_97	Subject_97	Baseline	Healthy	Black or African American	V4	Blood					https://doi.org/10.21417/ADPT2020V4CD	
Subject_98	Subject_98	Baseline	Healthy	Black or African American	V4	Blood					https://doi.org/10.21417/ADPT2020V4CD	
Subject_99	Subject_99	Baseline	Healthy	Black or African American	V4	Blood					https://doi.org/10.21417/ADPT2020V4CD	
NHMM-MC-0781-001	MGUS-001	Baseline	MGUS	Black or African American	V4	PBMC						
NHMM-MC-1025-001	MGUS-001	Follow-up	MGUS	Black or African American	V4	PBMC						632
NHMM-MC-0159-001	MGUS-002	Baseline	MGUS	Black or African American	V4	PBMC						
NHMM-MC-0839-002	MGUS-002	Follow-up	MGUS	Black or African American	V4	PBMC						728
NHMM-MC-0238-001	MGUS-003	Baseline	MGUS	Asian	V4	PBMC						
NHMM-MC-0678-001	MGUS-003	Follow-up	MGUS	Asian	V4	PBMC						385
NHMM-MC-0043-001	MGUS-004	Baseline	MGUS	Caucasian	V4	PBMC						
NHMM-MC-0666-001	MGUS-004	Follow-up	MGUS	Caucasian	V4	PBMC						560
NHMM-MC-0882-001	MGUS-005	Baseline	MGUS	Caucasian	V4	PBMC						
NHMM-MC-0966-001	MGUS-005	Follow-up	MGUS	Caucasian	V4	PBMC						194
NHMM-MC-0143-001	MGUS-006	Baseline	MGUS	Caucasian	V4	PBMC						
NHMM-MC-0141-001	MGUS-007	Baseline	MGUS	Caucasian	V4	PBMC						
NHMM-MC-0280-001	MGUS-008	Baseline	MGUS	Caucasian	V4	PBMC						
NHMM-MC-0921-002	MGUS-008	Follow-up	MGUS	Caucasian	V4	PBMC						768
NHMM-MC-0226-001	MGUS-009	Baseline	MGUS	Caucasian	V4	PBMC						
NHMM-MC-0866-001	MGUS-009	Follow-up	MGUS	Caucasian	V4	PBMC						727
NHMM-MC-0005-001	MGUS-010	Baseline	MGUS	Black or African American	V4	PBMC						
NHMM-MC-0946-001	MGUS-010	Follow-up	MGUS	Black or African American	V4	PBMC						1099
NHMM-MC-1021-001	MGUS-011	Baseline	MGUS	Caucasian	V4	PBMC						
NHMM-MC-0936-001	MGUS-011	Follow-up	MGUS	Caucasian	V4	PBMC						358
NHMM-MC-0041-001	MGUS-012	Baseline	MGUS	Black or African American	V4	PBMC						
NHMM-MC-0791-001	MGUS-012	Follow-up	MGUS	Black or African American	V4	PBMC						747
NHMM-MC-0504-001	MGUS-013	Baseline	MGUS	Black or African American	V4	PBMC						

S1 Table. Individual sample characteristics (n = 612). Minimal residual disease (MRD) assessment is shown for smolder multiple myeloma (SMM) samples after cycles 1, 4, 8, 20, and 32 of carfilzomib, lenalidomide, and dexamethasone (KRd). Deepest response assessed by the International Myeloma Working Group following induction therapy for multiple myeloma (MM) is shown. Monoclonal gammopathy of undetermined signifiance (MGUS) samples are from diagnose and follow-up of untreated patients. Healthy samples

Sample	Patient	Time point	Diagnosis	Race	ImmunoSeq version	Tissue	KRd Cycle 8	KRd Cycle 20	KRd Cycle 32	Induction best response	Source of healthy data	Follow-up days for MGUS
NHMM-MC-0986-001	MGUS-013	Follow-up	MGUS	Black or African American	V4	PBMC						728
NHMM-MC-0189-001	MGUS-014	Baseline	MGUS	Caucasian	V4	PBMC						
NHMM-MC-0858-002	MGUS-014	Follow-up	MGUS	Caucasian	V4	PBMC						743
NHMM-MC-0438-001	MGUS-015	Baseline	MGUS	Caucasian	V4	PBMC						
NHMM-MC-0968-001	MGUS-015	Follow-up	MGUS	Caucasian	V4	PBMC						744
NHMM-MC-0167-001	MGUS-016	Baseline	MGUS	Caucasian	V4	PBMC						
NHMM-MC-0832-001	MGUS-016	Follow-up	MGUS	Caucasian	V4	PBMC						726
NHMM-MC-0149-001	MGUS-017	Baseline	MGUS	Caucasian	V4	PBMC						
NHMM-MC-0824-001	MGUS-017	Follow-up	MGUS	Caucasian	V4	PBMC						713
NHMM-MC-0125-001	MGUS-018	Baseline	MGUS	Caucasian	V4	PBMC						
NHMM-MC-0027-001	MGUS-019	Baseline	MGUS	Caucasian	V4	PBMC						
NHMM-MC-0869-002	MGUS-019	Follow-up	MGUS	Caucasian	V4	PBMC						914
NHMM-MC-0877-001	MGUS-020	Baseline	MGUS	Caucasian	V4	PBMC						
NHMM-MC-0963-001	MGUS-020	Follow-up	MGUS	Caucasian	V4	PBMC						180
NHMM-MC-0137-001	MGUS-021	Baseline	MGUS	Caucasian	V4	PBMC						
NHMM-MC-0191-001	MGUS-022	Baseline	MGUS	Caucasian	V4	PBMC						
NHMM-MC-0822-001	MGUS-022	Follow-up	MGUS	Caucasian	V4	PBMC						693
NHMM-MC-0508-001	MGUS-023	Baseline	MGUS	Unknown	V4	PBMC						
NHMM-MC-0723-001	MGUS-023	Follow-up	MGUS	Unknown	V4	PBMC						203
NHMM-MC-0023-001	MGUS-024	Baseline	MGUS	Caucasian	V4	PBMC						
NHMM-MC-0214-001	MGUS-024	Follow-up	MGUS	Caucasian	V4	PBMC						182
NHMM-MC-0550-001	MGUS-025	Baseline	MGUS	Caucasian	V4	PBMC						
NHMM-MC-0837-001	MGUS-025	Follow-up	MGUS	Caucasian	V4	PBMC						369
NHMM-MC-0208-001	MGUS-026	Baseline	MGUS	Caucasian	V4	PBMC						
NHMM-MC-0848-001	MGUS-026	Follow-up	MGUS	Caucasian	V4	PBMC						715
NHMM-MC-0057-001	MGUS-027	Baseline	MGUS	Black or African American	V4	PBMC						
NHMM-MC-0452-001	MGUS-027	Follow-up	MGUS	Black or African American	V4	PBMC						357
NHMM-MC-0320-001	MGUS-028	Baseline	MGUS	Caucasian	V4	PBMC						
NHMM-MC-0876-002	MGUS-028	Follow-up	MGUS	Caucasian	V4	PBMC						660
NHMM-MC-0364-001	MGUS-029	Baseline	MGUS	Caucasian	V4	PBMC						
NHMM-MC-0942-001	MGUS-029	Follow-up	MGUS	Caucasian	V4	PBMC						737
NHMM-MC-0029-001	MGUS-030	Baseline	MGUS	Caucasian	V4	PBMC						
NHMM-MC-0888-002	MGUS-030	Follow-up	MGUS	Caucasian	V4	PBMC						945
NHMM-MC-0314-001	MGUS-031	Baseline	MGUS	Black or African American	V4	PBMC						
NHMM-MC-0047-001	MGUS-032	Baseline	MGUS	Black or African American	V4	PBMC						
NHMM-MC-0890-002	MGUS-032	Follow-up	MGUS	Black or African American	V4	PBMC						917
NHMM-MC-0161-001	MGUS-033	Baseline	MGUS	Caucasian	V4	PBMC						
NHMM-MC-0760-001	MGUS-033	Follow-up	MGUS	Caucasian	V4	PBMC						562
NHMM-MC-0013-001	MGUS-034	Baseline	MGUS	Black or African American	V4	PBMC						
NHMM-MC-0957-001	MGUS-034	Follow-up	MGUS	Black or African American	V4	PBMC						1113
NHMM-MC-0915-001	MGUS-035	Baseline	MGUS	Caucasian	V4	PBMC						
NHMM-MC-1049-001	MGUS-035	Follow-up	MGUS	Caucasian	V4	PBMC						378
NHMM-MC-0031-001	MGUS-036	Baseline	MGUS	Caucasian	V4	PBMC						
NHMM-MC-0630-001	MGUS-036	Follow-up	MGUS	Caucasian	V4	PBMC						546
NHMM-MC-0670-001	MGUS-038	Baseline	MGUS	Caucasian	V4	PBMC						
NHMM-MC-0382-001	MGUS-039	Baseline	MGUS	Caucasian	V4	PBMC						
NHMM-MC-0938-001	MGUS-039	Follow-up	MGUS	Caucasian	V4	PBMC						735
NHMM-MC-0784-001	MGUS-040	Baseline	MGUS	Caucasian	V4	PBMC						
NHMM-MC-0964-001	MGUS-040	Follow-up	MGUS	Caucasian	V4	PBMC						364
NHMM-MC-0276-001	MGUS-041	Baseline	MGUS	Caucasian	V4	PBMC						
NHMM-MC-0944-001	MGUS-041	Follow-up	MGUS	Caucasian	V4	PBMC						812
NHMM-MC-0035-001	MGUS-042	Baseline	MGUS	Caucasian	V4	PBMC						
NHMM-MC-0962-002	MGUS-042	Follow-up	MGUS	Caucasian	V4	PBMC						1097
NHMM-MC-0017-001	MGUS-043	Baseline	MGUS	Caucasian	V4	PBMC						
NHMM-MC-0950-002	MGUS-043	Follow-up	MGUS	Caucasian	V4	PBMC						1090
NHMM-MC-0081-001	MGUS-044	Baseline	MGUS	Caucasian	V4	PBMC						
NHMM-MC-0426-001	MGUS-044	Follow-up	MGUS	Caucasian	V4	PBMC						301
NHMM-MC-0061-001	MGUS-045	Baseline	MGUS	Black or African American	V4	PBMC						
NHMM-MC-0931-001	MGUS-045	Follow-up	MGUS	Black or African American	V4	PBMC						973
NHMM-MC-0097-001	MGUS-046	Baseline	MGUS	Caucasian	V4	PBMC						
NHMM-MC-0352-001	MGUS-046	Follow-up	MGUS	Caucasian	V4	PBMC						228
NHMM-MC-0021-001	MGUS-047	Baseline	MGUS	Caucasian	V4	PBMC						
NHMM-MC-0977-002	MGUS-047	Follow-up	MGUS	Caucasian	V4	PBMC						1149

S1 Table. Individual sample characteristics (n = 612). Minimal residual disease (MRD) assessment is shown for smolder multiple myeloma (SMM) samples after cycles 1, 4, 8, 20, and 32 of carfilzomib, lenalidomide, and dexamethasone (KRd). Deepest response assessed by the International Myeloma Working Group following induction therapy for multiple myeloma (MM) is shown. Monoclonal gammopathy of undetermined signifiance (MGUS) samples are from diagnose and follow-up of untreated patients. Healthy samples

Sample	Patient	Time point	Diagnosis	Race	ImmunoSeq version	Tissue	KRd Cycle 8	KRd Cycle 20	KRd Cycle 32	Induction best response	Source of healthy data	Follow-up days for MGUS
NHMM-MC-0268-001	MGUS-048	Baseline	MGUS	Caucasian	V4	PBMC						
NHMM-MC-0893-001	MGUS-048	Follow-up	MGUS	Caucasian	V4	PBMC						721
NHMM-MC-0780-001	MGUS-049	Baseline	MGUS	Caucasian	V4	PBMC						
NHMM-MC-0967-001	MGUS-049	Follow-up	MGUS	Caucasian	V4	PBMC						371
NHMM-MC-0458-001	MGUS-050	Baseline	MGUS	Caucasian	V4	PBMC						
NHMM-MC-0813-001	MGUS-050	Follow-up	MGUS	Caucasian	V4	PBMC						418
NHMM-MC-0049-001	MGUS-051	Baseline	MGUS	Caucasian	V4	PBMC						
NHMM-MC-0981-001	MGUS-051	Follow-up	MGUS	Caucasian	V4	PBMC						1125
NHMM-MC-0270-001	MGUS-052	Baseline	MGUS	Caucasian	V4	PBMC						
NHMM-MC-0898-001	MGUS-052	Follow-up	MGUS	Caucasian	V4	PBMC						728
NHMM-MC-0272-001	MGUS-053	Baseline	MGUS	Caucasian	V4	PBMC						
NHMM-MC-0672-001	MGUS-053	Follow-up	MGUS	Caucasian	V4	PBMC						364
NHMM-MC-0102-001	MGUS-054	Baseline	MGUS	Caucasian	V4	PBMC						
NHMM-MC-0296-001	MGUS-054	Follow-up	MGUS	Caucasian	V4	PBMC						182
NHMM-MC-0484-001	MGUS-055	Baseline	MGUS	Caucasian	V4	PBMC						
NHMM-MC-0978-001	MGUS-055	Follow-up	MGUS	Caucasian	V4	PBMC						742
NHMM-MC-0912-001	MGUS-056	Baseline	MGUS	Caucasian	V4	PBMC						
NHMM-MC-1047-001	MGUS-056	Follow-up	MGUS	Caucasian	V4	PBMC						454
NHMM-MC-0715-001	MGUS-057	Baseline	MGUS	Black or African American	V4	PBMC						
NHMM-MC-0902-001	MGUS-057	Follow-up	MGUS	Black or African American	V4	PBMC						350
NHMM-MC-0011-001	MGUS-058	Baseline	MGUS	Caucasian	V4	PBMC						
NHMM-MC-0386-001	MGUS-058	Follow-up	MGUS	Caucasian	V4	PBMC						364
NHMM-MC-0412-001	MGUS-059	Baseline	MGUS	Caucasian	V4	PBMC						
NHMM-MC-0768-002	MGUS-059	Follow-up	MGUS	Caucasian	V4	PBMC						369
NHMM-MC-0454-001	MGUS-060	Baseline	MGUS	Caucasian	V4	PBMC						
NHMM-MC-0829-001	MGUS-060	Follow-up	MGUS	Caucasian	V4	PBMC						452
NHMM-MC-0171-001	MGUS-061	Baseline	MGUS	Black or African American	V4	PBMC						
NHMM-MC-0985-002	MGUS-061	Follow-up	MGUS	Black or African American	V4	PBMC						1034
NHMM-MC-0400-001	MGUS-062	Baseline	MGUS	Caucasian	V4	PBMC						
NHMM-MC-0958-001	MGUS-062	Follow-up	MGUS	Caucasian	V4	PBMC						754
NHMM-MC-0586-001	MGUS-063	Baseline	MGUS	Caucasian	V4	PBMC						
NHMM-MC-0889-001	MGUS-063	Follow-up	MGUS	Caucasian	V4	PBMC						434
NHMM-MC-0099-001	MGUS-064	Baseline	MGUS	Caucasian	V4	PBMC						
NHMM-MC-0807-001	MGUS-065	Baseline	MGUS	Caucasian	V4	PBMC						
NHMM-MC-0982-002	MGUS-065	Follow-up	MGUS	Caucasian	V4	PBMC						362
NHMM-MC-0448-001	MGUS-066	Baseline	MGUS	Caucasian	V4	PBMC						
NHMM-MC-0965-001	MGUS-066	Follow-up	MGUS	Caucasian	V4	PBMC						733
NHMM-MC-0258-001	MGUS-067	Baseline	MGUS	Caucasian	V4	PBMC						
NHMM-MC-0674-001	MGUS-067	Follow-up	MGUS	Caucasian	V4	PBMC						367
NHMM-MC-0642-001	MGUS-068	Baseline	MGUS	Caucasian	V4	PBMC						
NHMM-MC-0975-001	MGUS-068	Follow-up	MGUS	Caucasian	V4	PBMC						588
NHMM-MC-0901-001	MGUS-069	Baseline	MGUS	Caucasian	V4	PBMC						
NHMM-MC-0632-001	MGUS-070	Baseline	MGUS	Caucasian	V4	PBMC						
NHMM-MC-0816-001	MGUS-070	Follow-up	MGUS	Caucasian	V4	PBMC						273
NHMM-MC-0916-001	MGUS-071	Baseline	MGUS	Caucasian	V4	PBMC						
NHMM-MC-0988-001	MGUS-071	Follow-up	MGUS	Caucasian	V4	PBMC						182
NHMM-MC-0131-001	MGUS-072	Baseline	MGUS	Caucasian	V4	PBMC						
NHMM-MC-0989-001	MGUS-072	Follow-up	MGUS	Caucasian	V4	PBMC						1075
NHMM-MC-0894-002	MGUS-073	Baseline	MGUS	Caucasian	V4	PBMC						
NHMM-MC-1056-001	MGUS-073	Follow-up	MGUS	Caucasian	V4	PBMC						181
NHMM-MC-0109-001	MGUS-074	Baseline	MGUS	Caucasian	V4	PBMC						
NHMM-MC-0526-001	MGUS-074	Follow-up	MGUS	Caucasian	V4	PBMC						382
NHMM-MC-0157-001	MGUS-075	Baseline	MGUS	Asian	V4	PBMC						
NHMM-MC-0840-001	MGUS-075	Follow-up	MGUS	Asian	V4	PBMC						728
NHMM-MC-0440-001	MGUS-076	Baseline	MGUS	Black or African American	V4	PBMC						
NHMM-MC-0960-001	MGUS-076	Follow-up	MGUS	Black or African American	V4	PBMC						719
NHMM-MC-0145-001	MGUS-077	Baseline	MGUS	Black or African American	V4	PBMC						
NHMM-MC-0830-001	MGUS-077	Follow-up	MGUS	Black or African American	V4	PBMC						733
NHMM-MC-0552-001	MGUS-078	Baseline	MGUS	Caucasian	V4	PBMC						
NHMM-MC-0853-001	MGUS-078	Follow-up	MGUS	Caucasian	V4	PBMC						397
NHMM-MC-0362-001	MGUS-079	Baseline	MGUS	Asian	V4	PBMC						
NHMM-MC-0920-001	MGUS-079	Follow-up	MGUS	Asian	V4	PBMC						700
NHMM-MC-0169-001	MGUS-080	Baseline	MGUS	Caucasian	V4	PBMC						

S1 Table. Individual sample characteristics (n = 612). Minimal residual disease (MRD) assessment is shown for smolder multiple myeloma (SMM) samples after cycles 1, 4, 8, 20, and 32 of carfilzomib, lenalidomide, and dexamethasone (KRd). Deepest response assessed by the International Myeloma Working Group following induction therapy for multiple myeloma (MM) is shown. Monoclonal gammopathy of undetermined signifiance (MGUS) samples are from diagnose and follow-up of untreated patients. Healthy samples

Sample	Patient	Time point	Diagnosis	Race	ImmunoSeq version	Tissue	KRd Cycle 8	KRd Cycle 20	KRd Cycle 32	Induction best response	Source of healthy data	Follow-up days for MGUS
NHMM-MC-0856-001	MGUS-080	Follow-up	MGUS	Caucasian	V4	PBMC						757
NHMM-MC-0054-001	MGUS-081	Baseline	MGUS	Caucasian	V4	PBMC						
NHMM-MC-0648-001	MGUS-081	Follow-up	MGUS	Caucasian	V4	PBMC						525
MM-PBMC-256	MM-001	Baseline	MM	Caucasian	V3	PBMC				PR		
MM-PBMC-234	MM-002	Baseline	MM	Caucasian	V3	PBMC				MRD negative		
MM-PBMC-328	MM-003	Baseline	MM	Caucasian	V3	PBMC				nCR		
MM-PBMC-347	MM-004	Baseline	MM	Caucasian	V3	PBMC				VGPR		
MM-PBMC-339	MM-005	Baseline	MM	Caucasian	V3	PBMC				MRD negative		
MM-PBMC-355	MM-006	Baseline	MM	Caucasian	V3	PBMC				MRD negative		
MM-PBMC-364	MM-007	Baseline	MM	Black or African American	V3	PBMC				MRD negative		
MM-PBMC-388	MM-008	Baseline	MM	Caucasian	V3	PBMC				MRD negative		
MM-PBMC-386	MM-009	Baseline	MM	Caucasian	V3	PBMC				VGPR		
MM-PBMC-395	MM-010	Baseline	MM	Caucasian	V3	PBMC				PR		
MM-PBMC-014	MM-011	Baseline	MM	Black or African American	V3	PBMC				MRD negative		
MM-PBMC-116	MM-012	Baseline	MM	Caucasian	V3	PBMC				MRD negative		
MM-PBMC-174	MM-013	Baseline	MM	Black or African American	V3	PBMC				MRD negative		
MM-PBMC-188	MM-014	Baseline	MM	Caucasian	V3	PBMC				MRD negative		
MM-PBMC-426	MM-015	Baseline	MM	Caucasian	V3	PBMC				VGPR		
MM-PBMC-441	MM-016	Baseline	MM	Caucasian	V3	PBMC				MRD negative		
MM-PBMC-452	MM-017	Baseline	MM	Caucasian	V3	PBMC				MRD negative		
MM-PBMC-474	MM-018	Baseline	MM	Caucasian	V3	PBMC				nCR		
MM-PBMC-476	MM-019	Baseline	MM	Caucasian	V3	PBMC				VGPR		
MM-PBMC-496	MM-020	Baseline	MM	Caucasian	V3	PBMC				VGPR		
MM-PBMC-491	MM-021	Baseline	MM	Caucasian	V3	PBMC				MRD negative		
MM-PBMC-509	MM-022	Baseline	MM	Caucasian	V3	PBMC				VGPR		
MM-PBMC-513	MM-023	Baseline	MM	Caucasian	V3	PBMC				MRD negative		
MM-PBMC-524	MM-024	Baseline	MM	Caucasian	V3	PBMC				MRD negative		
MM-PBMC-566	MM-025	Baseline	MM	Black or African American	V3	PBMC				nCR		
MM-PBMC-600	MM-026	Baseline	MM	Caucasian	V3	PBMC				MRD negative		
MM-PBMC-621	MM-027	Baseline	MM	Caucasian	V3	PBMC				MRD negative		
MM-PBMC-653	MM-028	Baseline	MM	Caucasian	V3	PBMC				CR		
MM-PBMC-666	MM-029	Baseline	MM	Caucasian	V3	PBMC				MRD negative		
MM-PBMC-697	MM-030	Baseline	MM	Caucasian	V3	PBMC				PR		
MM-PBMC-707	MM-031	Baseline	MM	Caucasian	V3	PBMC				sCR		
SMM-PBMC-001	SMM-001	Baseline	SMM	Caucasian	V4	PBMC	MRD positive					
CRDS-MC-0017-001	SMM-001	Cycle 1	SMM	Caucasian	V4	PBMC	MRD positive					
SMM-PBMC-275	SMM-001	Cycle 32	SMM	Caucasian	V4	PBMC	MRD positive					
CRDS-MC-0048-001	SMM-001	Cycle 4	SMM	Caucasian	V4	PBMC	MRD positive					
SMM-PBMC-068	SMM-001	Cycle 8	SMM	Caucasian	V4	PBMC	MRD positive					
SMM-PBMC-008	SMM-002	Baseline	SMM	Caucasian	V4	PBMC	MRD negative	MRD positive	MRD positive			
CRDS-MC-0019-001	SMM-002	Cycle 1	SMM	Caucasian	V4	PBMC	MRD negative	MRD positive	MRD positive			
SMM-PBMC-196	SMM-002	Cycle 20	SMM	Caucasian	V4	PBMC	MRD negative	MRD positive	MRD positive			
SMM-PBMC-336	SMM-002	Cycle 32	SMM	Caucasian	V4	PBMC	MRD negative	MRD positive	MRD positive			
CRDS-MC-0050-001	SMM-002	Cycle 4	SMM	Caucasian	V4	PBMC	MRD negative	MRD positive	MRD positive			
SMM-PBMC-087	SMM-002	Cycle 8	SMM	Caucasian	V4	PBMC	MRD negative	MRD positive	MRD positive			
SMM-PBMC-023	SMM-003	Baseline	SMM	Caucasian	V4	PBMC	MRD negative	MRD negative	MRD negative			
CRDS-MC-0043-001	SMM-003	Cycle 1	SMM	Caucasian	V4	PBMC	MRD negative	MRD negative	MRD negative			
SMM-PBMC-221	SMM-003	Cycle 20	SMM	Caucasian	V4	PBMC	MRD negative	MRD negative	MRD negative			
SMM-PBMC-389	SMM-003	Cycle 32	SMM	Caucasian	V4	PBMC	MRD negative	MRD negative	MRD negative			
CRDS-MC-0061-001	SMM-003	Cycle 4	SMM	Caucasian	V4	PBMC	MRD negative	MRD negative	MRD negative			
SMM-PBMC-100	SMM-003	Cycle 8	SMM	Caucasian	V4	PBMC	MRD negative	MRD negative	MRD negative			
SMM-PBMC-033	SMM-004	Baseline	SMM	Caucasian	V4	PBMC	MRD negative	MRD positive	MRD negative			
CRDS-MC-0046-001	SMM-004	Cycle 1	SMM	Caucasian	V4	PBMC	MRD negative	MRD positive	MRD negative			
SMM-PBMC-227	SMM-004	Cycle 20	SMM	Caucasian	V4	PBMC	MRD negative	MRD positive	MRD negative			
SMM-PBMC-395	SMM-004	Cycle 32	SMM	Caucasian	V4	PBMC	MRD negative	MRD positive	MRD negative			
CRDS-MC-0069-001	SMM-004	Cycle 4	SMM	Caucasian	V4	PBMC	MRD negative	MRD positive	MRD negative			
SMM-PBMC-108	SMM-004	Cycle 8	SMM	Caucasian	V4	PBMC	MRD negative	MRD positive	MRD negative			
SMM-PBMC-054	SMM-005	Baseline	SMM	Caucasian	V4	PBMC	MRD negative	MRD negative	MRD negative			
CRDS-MC-0062-001	SMM-005	Cycle 1	SMM	Caucasian	V4	PBMC	MRD negative	MRD negative	MRD negative			
SMM-PBMC-255	SMM-005	Cycle 20	SMM	Caucasian	V4	PBMC	MRD negative	MRD negative	MRD negative			
SMM-PBMC-407	SMM-005	Cycle 32	SMM	Caucasian	V4	PBMC	MRD negative	MRD negative	MRD negative			
CRDS-MC-0091-001	SMM-005	Cycle 4	SMM	Caucasian	V4	PBMC	MRD negative	MRD negative	MRD negative			
SMM-PBMC-143	SMM-005	Cycle 8	SMM	Caucasian	V4	PBMC	MRD negative	MRD negative	MRD negative			

S1 Table. Individual sample characteristics (n = 612). Minimal residual disease (MRD) assessment is shown for smolder multiple myeloma (SMM) samples after cycles 1, 4, 8, 20, and 32 of carfilzomib, lenalidomide, and dexamethasone (KRd). Deepest response assessed by the International Myeloma Working Group following induction therapy for multiple myeloma (MM) is shown. Monoclonal gammopathy of undetermined signifiance (MGUS) samples are from diagnose and follow-up of untreated patients. Healthy samples

Sample	Patient	Time point	Diagnosis	Race	ImmunoSeq version	Tissue	KRd Cycle 8	KRd Cycle 20	KRd Cycle 32	Induction best response	Source of healthy data	Follow-up days for MGUS
SMM-PBMC-063	SMM-006	Baseline	SMM	Caucasian	V4	PBMC	MRD negative	MRD positive	MRD positive			
CRDS-MC-0072-001	SMM-006	Cycle 1	SMM	Caucasian	V4	PBMC	MRD negative	MRD positive	MRD positive			
SMM-PBMC-265	SMM-006	Cycle 20	SMM	Caucasian	V4	PBMC	MRD negative	MRD positive	MRD positive			
SMM-PBMC-412	SMM-006	Cycle 32	SMM	Caucasian	V4	PBMC	MRD negative	MRD positive	MRD positive			
CRDS-MC-0111-001	SMM-006	Cycle 4	SMM	Caucasian	V4	PBMC	MRD negative	MRD positive	MRD positive			
SMM-PBMC-153	SMM-006	Cycle 8	SMM	Caucasian	V4	PBMC	MRD negative	MRD positive	MRD positive			
SMM-PBMC-073	SMM-007	Baseline	SMM	Caucasian	V4	PBMC	MRD negative	MRD negative	MRD negative			
CRDS-MC-0089-001	SMM-007	Cycle 1	SMM	Caucasian	V4	PBMC	MRD negative	MRD negative	MRD negative			
SMM-PBMC-277	SMM-007	Cycle 20	SMM	Caucasian	V4	PBMC	MRD negative	MRD negative	MRD negative			
SMM-PBMC-415	SMM-007	Cycle 32	SMM	Caucasian	V4	PBMC	MRD negative	MRD negative	MRD negative			
CRDS-MC-0126-001	SMM-007	Cycle 4	SMM	Caucasian	V4	PBMC	MRD negative	MRD negative	MRD negative			
SMM-PBMC-159	SMM-007	Cycle 8	SMM	Caucasian	V4	PBMC	MRD negative	MRD negative	MRD negative			
SMM-PBMC-074	SMM-008	Baseline	SMM	Black or African American	V4	PBMC	MRD negative	MRD negative	MRD negative			
CRDS-MC-0088-001	SMM-008	Cycle 1	SMM	Black or African American	V4	PBMC	MRD negative	MRD negative	MRD negative			
SMM-PBMC-273	SMM-008	Cycle 20	SMM	Black or African American	V4	PBMC	MRD negative	MRD negative	MRD negative			
SMM-PBMC-416	SMM-008	Cycle 32	SMM	Black or African American	V4	PBMC	MRD negative	MRD negative	MRD negative			
CRDS-MC-0119-001	SMM-008	Cycle 4	SMM	Black or African American	V4	PBMC	MRD negative	MRD negative	MRD negative			
SMM-PBMC-157	SMM-008	Cycle 8	SMM	Black or African American	V4	PBMC	MRD negative	MRD negative	MRD negative			
SMM-PBMC-096	SMM-009	Baseline	SMM	Caucasian	V4	PBMC	MRD negative		MRD negative			
CRDS-MC-0110-001	SMM-009	Cycle 1	SMM	Caucasian	V4	PBMC	MRD negative		MRD negative			
CRDS-MC-0268-001	SMM-009	Cycle 20	SMM	Caucasian	V4	PBMC	MRD negative		MRD negative			
CRDS-MC-0146-001	SMM-009	Cycle 4	SMM	Caucasian	V4	PBMC	MRD negative		MRD negative			
SMM-PBMC-173	SMM-009	Cycle 8	SMM	Caucasian	V4	PBMC	MRD negative		MRD negative			
SMM-PBMC-103	SMM-010	Baseline	SMM	Caucasian	V4	PBMC	MRD negative	MRD negative	MRD negative			
CRDS-MC-0113-001	SMM-010	Cycle 1	SMM	Caucasian	V4	PBMC	MRD negative	MRD negative	MRD negative			
SMM-PBMC-296	SMM-010	Cycle 20	SMM	Caucasian	V4	PBMC	MRD negative	MRD negative	MRD negative			
SMM-PBMC-425	SMM-010	Cycle 32	SMM	Caucasian	V4	PBMC	MRD negative	MRD negative	MRD negative			
CRDS-MC-0148-001	SMM-010	Cycle 4	SMM	Caucasian	V4	PBMC	MRD negative	MRD negative	MRD negative			
SMM-PBMC-177	SMM-010	Cycle 8	SMM	Caucasian	V4	PBMC	MRD negative	MRD negative	MRD negative			
SMM-PBMC-112	SMM-011	Baseline	SMM	Caucasian	V4	PBMC	MRD negative	MRD negative	MRD negative			
CRDS-MC-0129-001	SMM-011	Cycle 1	SMM	Caucasian	V4	PBMC	MRD negative	MRD negative	MRD negative			
SMM-PBMC-299	SMM-011	Cycle 20	SMM	Caucasian	V4	PBMC	MRD negative	MRD negative	MRD negative			
SMM-PBMC-427	SMM-011	Cycle 32	SMM	Caucasian	V4	PBMC	MRD negative	MRD negative	MRD negative			
CRDS-MC-0155-001	SMM-011	Cycle 4	SMM	Caucasian	V4	PBMC	MRD negative	MRD negative	MRD negative			
SMM-PBMC-184	SMM-011	Cycle 8	SMM	Caucasian	V4	PBMC	MRD negative	MRD negative	MRD negative			
SMM-PBMC-115	SMM-012	Baseline	SMM	Caucasian	V4	PBMC	MRD negative	MRD negative	MRD negative			
CRDS-MC-0132-001	SMM-012	Cycle 1	SMM	Caucasian	V4	PBMC	MRD negative	MRD negative	MRD negative			
SMM-PBMC-301	SMM-012	Cycle 20	SMM	Caucasian	V4	PBMC	MRD negative	MRD negative	MRD negative			
SMM-PBMC-429	SMM-012	Cycle 32	SMM	Caucasian	V4	PBMC	MRD negative	MRD negative	MRD negative			
CRDS-MC-0156-001	SMM-012	Cycle 4	SMM	Caucasian	V4	PBMC	MRD negative	MRD negative	MRD negative			
SMM-PBMC-187	SMM-012	Cycle 8	SMM	Caucasian	V4	PBMC	MRD negative	MRD negative	MRD negative			
SMM-PBMC-179	SMM-013	Baseline	SMM	Caucasian	V4	PBMC	MRD positive	MRD positive	MRD positive			
CRDS-MC-0185-001	SMM-013	Cycle 1	SMM	Caucasian	V4	PBMC	MRD positive	MRD positive	MRD positive			
SMM-PBMC-413	SMM-013	Cycle 20	SMM	Caucasian	V4	PBMC	MRD positive	MRD positive	MRD positive			
SMM-PBMC-446	SMM-013	Cycle 32	SMM	Caucasian	V3	PBMC	MRD positive	MRD positive	MRD positive			
CRDS-MC-0228-001	SMM-013	Cycle 4	SMM	Caucasian	V4	PBMC	MRD positive	MRD positive	MRD positive			
SMM-PBMC-267	SMM-013	Cycle 8	SMM	Caucasian	V4	PBMC	MRD positive	MRD positive	MRD positive			
SMM-PBMC-188	SMM-014	Baseline	SMM	Caucasian	V4	PBMC	MRD negative	MRD negative	MRD negative			
CRDS-MC-0195-001	SMM-014	Cycle 1	SMM	Caucasian	V4	PBMC	MRD negative	MRD negative	MRD negative			
SMM-PBMC-420	SMM-014	Cycle 20	SMM	Caucasian	V4	PBMC	MRD negative	MRD negative	MRD negative			
SMM-PBMC-440	SMM-014	Cycle 32	SMM	Caucasian	V4	PBMC	MRD negative	MRD negative	MRD negative			
CRDS-MC-0245-001	SMM-014	Cycle 4	SMM	Caucasian	V4	PBMC	MRD negative	MRD negative	MRD negative			
SMM-PBMC-288	SMM-014	Cycle 8	SMM	Caucasian	V4	PBMC	MRD negative	MRD negative	MRD negative			
SMM-PBMC-190	SMM-015	Baseline	SMM	Caucasian	V4	PBMC	MRD negative	MRD negative	MRD negative			
CRDS-MC-0197-001	SMM-015	Cycle 1	SMM	Caucasian	V4	PBMC	MRD negative	MRD negative	MRD negative			
SMM-PBMC-418	SMM-015	Cycle 20	SMM	Caucasian	V4	PBMC	MRD negative	MRD negative	MRD negative			
SMM-PBMC-438	SMM-015	Cycle 32	SMM	Caucasian	V4	PBMC	MRD negative	MRD negative	MRD negative			
CRDS-MC-0239-001	SMM-015	Cycle 4	SMM	Caucasian	V4	PBMC	MRD negative	MRD negative	MRD negative			
SMM-PBMC-279	SMM-015	Cycle 8	SMM	Caucasian	V4	PBMC	MRD negative	MRD negative	MRD negative			
SMM-PBMC-209	SMM-016	Baseline	SMM	Caucasian	V4	PBMC	MRD negative	MRD negative	MRD negative			
CRDS-MC-0216-001	SMM-016	Cycle 1	SMM	Caucasian	V4	PBMC	MRD negative	MRD negative	MRD negative			
SMM-PBMC-422	SMM-016	Cycle 20	SMM	Caucasian	V4	PBMC	MRD negative	MRD negative	MRD negative			
CRDS-MC-0249-001	SMM-016	Cycle 4	SMM	Caucasian	V4	PBMC	MRD negative	MRD negative	MRD negative			

S1 Table. Individual sample characteristics (n = 612). Minimal residual disease (MRD) assessment is shown for smolder multiple myeloma (SMM) samples after cycles 1, 4, 8, 20, and 32 of carfilzomib, lenalidomide, and dexamethasone (KRd). Deepest response assessed by the International Myeloma Working Group following induction therapy for multiple myeloma (MM) is shown. Monoclonal gammopathy of undetermined signifiance (MGUS) samples are from diagnose and follow-up of untreated patients. Healthy samples

Sample	Patient	Time point	Diagnosis	Race	ImmunoSeq version	Tissue	KRd Cycle 8	KRd Cycle 20	KRd Cycle 32	Induction best response	Source of healthy data	Follow-up days for MGUS
SMM-PBMC-291	SMM-016	Cycle 8	SMM	Caucasian	V4	PBMC	MRD negative	MRD negative	MRD negative			
SMM-PBMC-201	SMM-017	Baseline	SMM	Black or African American	V4	PBMC	MRD negative	MRD positive	MRD positive			
CRDS-MC-0220-001	SMM-017	Cycle 1	SMM	Black or African American	V4	PBMC	MRD negative	MRD positive	MRD positive			
SMM-PBMC-421	SMM-017	Cycle 20	SMM	Black or African American	V4	PBMC	MRD negative	MRD positive	MRD positive			
SMM-PBMC-441	SMM-017	Cycle 32	SMM	Black or African American	V4	PBMC	MRD negative	MRD positive	MRD positive			
CRDS-MC-0248-001	SMM-017	Cycle 4	SMM	Black or African American	V4	PBMC	MRD negative	MRD positive	MRD positive			
SMM-PBMC-290	SMM-017	Cycle 8	SMM	Black or African American	V4	PBMC	MRD negative	MRD positive	MRD positive			
SMM-PBMC-203	SMM-018	Baseline	SMM	Caucasian	V4	PBMC	MRD negative	MRD positive	MRD positive			
CRDS-MC-0219-001	SMM-018	Cycle 1	SMM	Caucasian	V4	PBMC	MRD negative	MRD positive	MRD positive			
SMM-PBMC-424	SMM-018	Cycle 20	SMM	Caucasian	V4	PBMC	MRD negative	MRD positive	MRD positive			
SMM-PBMC-443	SMM-018	Cycle 32	SMM	Caucasian	V4	PBMC	MRD negative	MRD positive	MRD positive			
CRDS-MC-0250-001	SMM-018	Cycle 4	SMM	Caucasian	V4	PBMC	MRD negative	MRD positive	MRD positive			
SMM-PBMC-289	SMM-018	Cycle 8	SMM	Caucasian	V4	PBMC	MRD negative	MRD positive	MRD positive			
SMM-PBMC-459	SMM-019	Baseline	SMM	Caucasian	V4	PBMC	MRD negative		MRD negative			
CRDS-MC-0463-001	SMM-019	Cycle 1	SMM	Caucasian	V4	PBMC	MRD negative		MRD negative			
CRDS-MC-0482-001	SMM-019	Cycle 20	SMM	Caucasian	V4	PBMC	MRD negative		MRD negative			
CRDS-MC-0501-001	SMM-019	Cycle 32	SMM	Caucasian	V4	PBMC	MRD negative		MRD negative			
CRDS-MC-0467-001	SMM-019	Cycle 4	SMM	Caucasian	V3	PBMC	MRD negative		MRD negative			
CRDS-MC-0474-001	SMM-019	Cycle 8	SMM	Caucasian	V3	PBMC	MRD negative		MRD negative			
SMM-PBMC-484	SMM-020	Baseline	SMM	Black or African American	V3	PBMC	MRD negative	MRD negative	MRD negative			
CRDS-MC-0497-001	SMM-020	Cycle 1	SMM	Black or African American	V3	PBMC	MRD negative	MRD negative	MRD negative			
CRDS-MC-0801-001	SMM-020	Cycle 20	SMM	Black or African American	V3	PBMC	MRD negative	MRD negative	MRD negative			
CRDS-MC-0949-001	SMM-020	Cycle 32	SMM	Black or African American	V3	PBMC	MRD negative	MRD negative	MRD negative			
CRDS-MC-0539-001	SMM-020	Cycle 4	SMM	Black or African American	V3	PBMC	MRD negative	MRD negative	MRD negative			
CRDS-MC-0614-001	SMM-020	Cycle 8	SMM	Black or African American	V3	PBMC	MRD negative	MRD negative	MRD negative			
SMM-PBMC-488	SMM-021	Baseline	SMM	Black or African American	V3	PBMC	MRD negative	MRD negative	MRD negative			
CRDS-MC-0498-001	SMM-021	Cycle 1	SMM	Black or African American	V3	PBMC	MRD negative	MRD negative	MRD negative			
CRDS-MC-0791-001	SMM-021	Cycle 20	SMM	Black or African American	V3	PBMC	MRD negative	MRD negative	MRD negative			
CRDS-MC-0534-001	SMM-021	Cycle 4	SMM	Black or African American	V4	PBMC	MRD negative	MRD negative	MRD negative			
CRDS-MC-0604-001	SMM-021	Cycle 8	SMM	Black or African American	V4	PBMC	MRD negative	MRD negative	MRD negative			
SMM-PBMC-461	SMM-022	Baseline	SMM	Black or African American	V4	PBMC			MRD negative			
CRDS-MC-0465-001	SMM-022	Cycle 1	SMM	Black or African American	V4	PBMC			MRD negative			
CRDS-MC-0768-001	SMM-022	Cycle 20	SMM	Black or African American	V4	PBMC			MRD negative			
CRDS-MC-0982-001	SMM-022	Cycle 32	SMM	Black or African American	V4	PBMC			MRD negative			
CRDS-MC-0487-001	SMM-022	Cycle 4	SMM	Black or African American	V4	PBMC			MRD negative			
CRDS-MC-0572-001	SMM-022	Cycle 8	SMM	Black or African American	V4	PBMC			MRD negative			
SMM-PBMC-473	SMM-023	Baseline	SMM	Black or African American	V4	PBMC	MRD negative	MRD positive	MRD positive			
CRDS-MC-0478-001	SMM-023	Cycle 1	SMM	Black or African American	V4	PBMC	MRD negative	MRD positive	MRD positive			
CRDS-MC-0764-001	SMM-023	Cycle 20	SMM	Black or African American	V4	PBMC	MRD negative	MRD positive	MRD positive			
CRDS-MC-0898-001	SMM-023	Cycle 32	SMM	Black or African American	V4	PBMC	MRD negative	MRD positive	MRD positive			
CRDS-MC-0503-001	SMM-023	Cycle 4	SMM	Black or African American	V4	PBMC	MRD negative	MRD positive	MRD positive			
CRDS-MC-0569-001	SMM-023	Cycle 8	SMM	Black or African American	V4	PBMC	MRD negative	MRD positive	MRD positive			
SMM-PBMC-483	SMM-024	Baseline	SMM	Caucasian	V4	PBMC	MRD negative	MRD negative	MRD negative			
CRDS-MC-0502-001	SMM-024	Cycle 1	SMM	Caucasian	V4	PBMC	MRD negative	MRD negative	MRD negative			
CRDS-MC-0779-001	SMM-024	Cycle 20	SMM	Caucasian	V4	PBMC	MRD negative	MRD negative	MRD negative			
CRDS-MC-0528-001	SMM-024	Cycle 4	SMM	Caucasian	V4	PBMC	MRD negative	MRD negative	MRD negative			
CRDS-MC-0591-001	SMM-024	Cycle 8	SMM	Caucasian	V4	PBMC	MRD negative	MRD negative	MRD negative			
CRDS-MC-0477-001	SMM-025	Baseline	SMM	Black or African American	V4	PBMC	MRD negative	MRD negative	MRD negative			
CRDS-MC-0486-001	SMM-025	Cycle 1	SMM	Black or African American	V4	PBMC	MRD negative	MRD negative	MRD negative			
CRDS-MC-0794-001	SMM-025	Cycle 20	SMM	Black or African American	V4	PBMC	MRD negative	MRD negative	MRD negative			
CRDS-MC-0947-001	SMM-025	Cycle 32	SMM	Black or African American	V4	PBMC	MRD negative	MRD negative	MRD negative			
CRDS-MC-0537-001	SMM-025	Cycle 4	SMM	Black or African American	V4	PBMC	MRD negative	MRD negative	MRD negative			
CRDS-MC-0599-001	SMM-025	Cycle 8	SMM	Black or African American	V4	PBMC	MRD negative	MRD negative	MRD negative			
CRDS-MC-0490-001	SMM-026	Baseline	SMM	Caucasian	V4	PBMC	MRD positive	MRD positive	MRD positive			
CRDS-MC-0508-001	SMM-026	Cycle 1	SMM	Caucasian	V4	PBMC	MRD positive	MRD positive	MRD positive			
CRDS-MC-0804-001	SMM-026	Cycle 20	SMM	Caucasian	V4	PBMC	MRD positive	MRD positive	MRD positive			
CRDS-MC-0950-001	SMM-026	Cycle 32	SMM	Caucasian	V4	PBMC	MRD positive	MRD positive	MRD positive			
CRDS-MC-0549-001	SMM-026	Cycle 4	SMM	Caucasian	V4	PBMC	MRD positive	MRD positive	MRD positive			
CRDS-MC-0613-001	SMM-026	Cycle 8	SMM	Caucasian	V4	PBMC	MRD positive	MRD positive	MRD positive			
CRDS-MC-0523-001	SMM-027	Baseline	SMM	Caucasian	V4	PBMC	MRD negative	MRD negative	MRD negative			
CRDS-MC-0541-001	SMM-027	Cycle 1	SMM	Caucasian	V4	PBMC	MRD negative	MRD negative	MRD negative			
CRDS-MC-0858-001	SMM-027	Cycle 20	SMM	Caucasian	V4	PBMC	MRD negative	MRD negative	MRD negative			
CRDS-MC-0607-001	SMM-027	Cycle 4	SMM	Caucasian	V4	PBMC	MRD negative	MRD negative	MRD negative			

S1 Table. Individual sample characteristics (n = 612). Minimal residual disease (MRD) assessment is shown for smolder multiple myeloma (SMM) samples after cycles 1, 4, 8, 20, and 32 of carfilzomib, lenalidomide, and dexamethasone (KRd). Deepest response assessed by the International Myeloma Working Group following induction therapy for multiple myeloma (MM) is shown. Monoclonal gammopathy of undetermined signifiance (MGUS) samples are from diagnose and follow-up of untreated patients. Healthy samples

Sample	Patient	Time point	Diagnosis	Race	ImmunoSeq version	Tissue	KRd Cycle 8	KRd Cycle 20	KRd Cycle 32	Induction best response	Source of healthy data	Follow-up days for MGUS
CRDS-MC-0675-001	SMM-027	Cycle 8	SMM	Caucasian	V4	PBMC	MRD negative	MRD negative	MRD negative			
CRDS-MC-0526-001	SMM-028	Baseline	SMM	Black or African American	V4	PBMC	MRD negative	MRD negative	MRD negative			
CRDS-MC-0543-001	SMM-028	Cycle 1	SMM	Black or African American	V4	PBMC	MRD negative	MRD negative	MRD negative			
CRDS-MC-0838-001	SMM-028	Cycle 20	SMM	Black or African American	V4	PBMC	MRD negative	MRD negative	MRD negative			
CRDS-MC-0589-001	SMM-028	Cycle 4	SMM	Black or African American	V3	PBMC	MRD negative	MRD negative	MRD negative			
CRDS-MC-0655-001	SMM-028	Cycle 8	SMM	Black or African American	V3	PBMC	MRD negative	MRD negative	MRD negative			
CRDS-MC-0536-001	SMM-029	Baseline	SMM	Caucasian	V3	PBMC	MRD negative	MRD negative	MRD negative			
CRDS-MC-0555-001	SMM-029	Cycle 1	SMM	Caucasian	V3	PBMC	MRD negative	MRD negative	MRD negative			
CRDS-MC-0846-001	SMM-029	Cycle 20	SMM	Caucasian	V3	PBMC	MRD negative	MRD negative	MRD negative			
CRDS-MC-0601-001	SMM-029	Cycle 4	SMM	Caucasian	V3	PBMC	MRD negative	MRD negative	MRD negative			
CRDS-MC-0663-001	SMM-029	Cycle 8	SMM	Caucasian	V3	PBMC	MRD negative	MRD negative	MRD negative			
CRDS-MC-0531-001	SMM-030	Baseline	SMM	Caucasian	V3	PBMC	MRD negative	MRD negative	MRD negative			
CRDS-MC-0544-001	SMM-030	Cycle 1	SMM	Caucasian	V3	PBMC	MRD negative	MRD negative	MRD negative			
CRDS-MC-0842-001	SMM-030	Cycle 20	SMM	Caucasian	V3	PBMC	MRD negative	MRD negative	MRD negative			
CRDS-MC-0594-001	SMM-030	Cycle 4	SMM	Caucasian	V3	PBMC	MRD negative	MRD negative	MRD negative			
CRDS-MC-0662-001	SMM-030	Cycle 8	SMM	Caucasian	V3	PBMC	MRD negative	MRD negative	MRD negative			
CRDS-MC-0548-001	SMM-031	Baseline	SMM	Caucasian	V3	PBMC	MRD positive	MRD positive	MRD positive			
CRDS-MC-0564-001	SMM-031	Cycle 1	SMM	Caucasian	V3	PBMC	MRD positive	MRD positive	MRD positive			
CRDS-MC-0878-001	SMM-031	Cycle 20	SMM	Caucasian	V3	PBMC	MRD positive	MRD positive	MRD positive			
CRDS-MC-0612-001	SMM-031	Cycle 4	SMM	Caucasian	V3	PBMC	MRD positive	MRD positive	MRD positive			
CRDS-MC-0680-001	SMM-031	Cycle 8	SMM	Caucasian	V3	PBMC	MRD positive	MRD positive	MRD positive			
CRDS-MC-0570-001	SMM-032	Baseline	SMM	Caucasian	V3	PBMC	MRD negative	MRD negative	MRD negative			
CRDS-MC-0587-001	SMM-032	Cycle 1	SMM	Caucasian	V3	PBMC	MRD negative	MRD negative	MRD negative			
CRDS-MC-0637-001	SMM-032	Cycle 4	SMM	Caucasian	V3	PBMC	MRD negative	MRD negative	MRD negative			
CRDS-MC-0695-001	SMM-032	Cycle 8	SMM	Caucasian	V3	PBMC	MRD negative	MRD negative	MRD negative			
CRDS-MC-0575-001	SMM-033	Baseline	SMM	Black or African American	V3	PBMC	MRD negative	MRD positive	MRD positive			
CRDS-MC-0593-001	SMM-033	Cycle 1	SMM	Black or African American	V3	PBMC	MRD negative	MRD positive	MRD positive			
CRDS-MC-0881-001	SMM-033	Cycle 20	SMM	Black or African American	V3	PBMC	MRD negative	MRD positive	MRD positive			
CRDS-MC-0645-001	SMM-033	Cycle 4	SMM	Black or African American	V3	PBMC	MRD negative	MRD positive	MRD positive			
CRDS-MC-0700-001	SMM-033	Cycle 8	SMM	Black or African American	V3	PBMC	MRD negative	MRD positive	MRD positive			
CRDS-MC-0615-001	SMM-034	Baseline	SMM	Caucasian	V3	PBMC	MRD negative	MRD negative	MRD positive			
CRDS-MC-0636-003	SMM-034	Cycle 1	SMM	Caucasian	V3	PBMC	MRD negative	MRD negative	MRD positive			
CRDS-MC-0888-001	SMM-034	Cycle 20	SMM	Caucasian	V3	PBMC	MRD negative	MRD negative	MRD positive			
CRDS-MC-0683-001	SMM-034	Cycle 4	SMM	Caucasian	V3	PBMC	MRD negative	MRD negative	MRD positive			
CRDS-MC-0739-001	SMM-034	Cycle 8	SMM	Caucasian	V3	PBMC	MRD negative	MRD negative	MRD positive			
CRDS-MC-0622-001	SMM-035	Baseline	SMM	Black or African American	V3	PBMC	MRD negative	MRD positive	MRD positive			
CRDS-MC-0643-001	SMM-035	Cycle 1	SMM	Black or African American	V3	PBMC	MRD negative	MRD positive	MRD positive			
CRDS-MC-0892-001	SMM-035	Cycle 20	SMM	Black or African American	V3	PBMC	MRD negative	MRD positive	MRD positive			
CRDS-MC-0687-001	SMM-035	Cycle 4	SMM	Black or African American	V3	PBMC	MRD negative	MRD positive	MRD positive			
CRDS-MC-0761-001	SMM-035	Cycle 8	SMM	Black or African American	V3	PBMC	MRD negative	MRD positive	MRD positive			
CRDS-MC-0628-001	SMM-036	Baseline	SMM	Black or African American	V3	PBMC	MRD positive	MRD positive	MRD positive			
CRDS-MC-0648-001	SMM-036	Cycle 1	SMM	Black or African American	V3	PBMC	MRD positive	MRD positive	MRD positive			
CRDS-MC-0900-001	SMM-036	Cycle 20	SMM	Black or African American	V3	PBMC	MRD positive	MRD positive	MRD positive			
CRDS-MC-0689-001	SMM-036	Cycle 4	SMM	Black or African American	V3	PBMC	MRD positive	MRD positive	MRD positive			
CRDS-MC-0763-001	SMM-036	Cycle 8	SMM	Black or African American	V3	PBMC	MRD positive	MRD positive	MRD positive			
CRDS-MC-0627-001	SMM-037	Baseline	SMM	Asian	V3	PBMC	MRD negative	MRD negative	MRD negative			
CRDS-MC-0647-001	SMM-037	Cycle 1	SMM	Asian	V3	PBMC	MRD negative	MRD negative	MRD negative			
CRDS-MC-0896-001	SMM-037	Cycle 20	SMM	Asian	V3	PBMC	MRD negative	MRD negative	MRD negative			
CRDS-MC-0690-001	SMM-037	Cycle 4	SMM	Asian	V3	PBMC	MRD negative	MRD negative	MRD negative			
CRDS-MC-0757-001	SMM-037	Cycle 8	SMM	Asian	V3	PBMC	MRD negative	MRD negative	MRD negative			
CRDS-MC-0653-001	SMM-038	Baseline	SMM	Black or African American	V3	PBMC	MRD positive	MRD positive	MRD positive			
CRDS-MC-0673-001	SMM-038	Cycle 1	SMM	Black or African American	V3	PBMC	MRD positive	MRD positive	MRD positive			
CRDS-MC-0919-001	SMM-038	Cycle 20	SMM	Black or African American	V3	PBMC	MRD positive	MRD positive	MRD positive			
CRDS-MC-0713-001	SMM-038	Cycle 4	SMM	Black or African American	V3	PBMC	MRD positive	MRD positive	MRD positive			
CRDS-MC-0778-001	SMM-038	Cycle 8	SMM	Black or African American	V3	PBMC	MRD positive	MRD positive	MRD positive			
CRDS-MC-0659-001	SMM-039	Baseline	SMM		V3	PBMC						
CRDS-MC-0679-001	SMM-040	Baseline	SMM	Caucasian	V3	PBMC	MRD negative	MRD negative	MRD negative			
CRDS-MC-0691-001	SMM-040	Cycle 1	SMM	Caucasian	V3	PBMC	MRD negative	MRD negative	MRD negative			
CRDS-MC-0750-001	SMM-040	Cycle 4	SMM	Caucasian	V3	PBMC	MRD negative	MRD negative	MRD negative			
CRDS-MC-0828-001	SMM-040	Cycle 8	SMM	Caucasian	V3	PBMC	MRD negative	MRD negative	MRD negative			
CRDS-MC-0694-001	SMM-041	Baseline	SMM	Black or African American	V3	PBMC	MRD negative	MRD negative	MRD negative			
CRDS-MC-0714-001	SMM-041	Cycle 1	SMM	Black or African American	V3	PBMC	MRD negative	MRD negative	MRD negative			
CRDS-MC-0766-001	SMM-041	Cycle 4	SMM	Black or African American	V3	PBMC	MRD negative	MRD negative	MRD negative			

S1 Table. Individual sample characteristics (n = 612). Minimal residual disease (MRD) assessment is shown for smolder multiple myeloma (SMM) samples after cycles 1, 4, 8, 20, and 32 of carfilzomib, lenalidomide, and dexamethasone (KRd). Deepest response assessed by the International Myeloma Working Group following induction therapy for multiple myeloma (MM) is shown. Monoclonal gammopathy of undetermined signifiance (MGUS) samples are from diagnose and follow-up of untreated patients. Healthy samples

Sample	Patient	Time point	Diagnosis	Race	ImmunoSeq version	Tissue	KRd Cycle 8	KRd Cycle 20	KRd Cycle 32	Induction best response	Source of healthy data	Follow-up days for MGUS
CRDS-MC-0834-001	SMM-041	Cycle 8	SMM	Black or African American	V3	PBMC	MRD negative	MRD negative	MRD negative			
CRDS-MC-0702-001	SMM-042	Baseline	SMM	Black or African American	V3	PBMC	MRD negative	MRD negative	MRD negative			
CRDS-MC-0723-001	SMM-042	Cycle 1	SMM	Black or African American	V3	PBMC	MRD negative	MRD negative	MRD negative			
CRDS-MC-0773-001	SMM-042	Cycle 4	SMM	Black or African American	V3	PBMC	MRD negative	MRD negative	MRD negative			
CRDS-MC-0830-001	SMM-042	Cycle 8	SMM	Black or African American	V3	PBMC	MRD negative	MRD negative	MRD negative			
CRDS-MC-0697-001	SMM-043	Baseline	SMM	Caucasian	V3	PBMC	MRD negative	MRD negative	MRD negative			
CRDS-MC-0719-001	SMM-043	Cycle 1	SMM	Caucasian	V3	PBMC	MRD negative	MRD negative	MRD negative			
CRDS-MC-0770-001	SMM-043	Cycle 4	SMM	Caucasian	V3	PBMC	MRD negative	MRD negative	MRD negative			
CRDS-MC-0825-001	SMM-043	Cycle 8	SMM	Caucasian	V3	PBMC	MRD negative	MRD negative	MRD negative			
CRDS-MC-0718-001	SMM-044	Baseline	SMM	Caucasian	V3	PBMC	MRD positive	MRD positive	MRD positive			
CRDS-MC-0735-001	SMM-044	Cycle 1	SMM	Caucasian	V3	PBMC	MRD positive	MRD positive	MRD positive			
CRDS-MC-0780-001	SMM-044	Cycle 4	SMM	Caucasian	V3	PBMC	MRD positive	MRD positive	MRD positive			
CRDS-MC-0839-001	SMM-044	Cycle 8	SMM	Caucasian	V3	PBMC	MRD positive	MRD positive	MRD positive			
CRDS-MC-0759-001	SMM-045	Baseline	SMM	Caucasian	V3	PBMC	MRD positive	MRD positive	MRD positive			
CRDS-MC-0775-001	SMM-045	Cycle 1	SMM	Caucasian	V3	PBMC	MRD positive	MRD positive	MRD positive			
CRDS-MC-0820-001	SMM-045	Cycle 4	SMM	Caucasian	V3	PBMC	MRD positive	MRD positive	MRD positive			
CRDS-MC-0874-001	SMM-045	Cycle 8	SMM	Caucasian	V3	PBMC	MRD positive	MRD positive	MRD positive			
CRDS-MC-0776-001	SMM-046	Baseline	SMM	Caucasian	V3	PBMC	MRD negative	MRD negative	MRD negative			
CRDS-MC-0784-001	SMM-046	Cycle 1	SMM	Caucasian	V3	PBMC	MRD negative	MRD negative	MRD negative			
CRDS-MC-0833-001	SMM-046	Cycle 4	SMM	Caucasian	V3	PBMC	MRD negative	MRD negative	MRD negative			
CRDS-MC-0885-001	SMM-046	Cycle 8	SMM	Caucasian	V3	PBMC	MRD negative	MRD negative	MRD negative			
CRDS-MC-0810-001	SMM-047	Baseline	SMM	Black or African American	V3	PBMC	MRD positive	MRD positive	MRD positive			
CRDS-MC-0835-001	SMM-047	Cycle 1	SMM	Black or African American	V3	PBMC	MRD positive	MRD positive	MRD positive			
CRDS-MC-0866-001	SMM-047	Cycle 4	SMM	Black or African American	V3	PBMC	MRD positive	MRD positive	MRD positive			
CRDS-MC-0886-001	SMM-047	Cycle 8	SMM	Black or African American	V3	PBMC	MRD positive	MRD positive	MRD positive			
CRDS-MC-0829-001	SMM-048	Baseline	SMM	Asian	V3	PBMC	MRD negative	MRD negative	MRD negative			
CRDS-MC-0844-001	SMM-048	Cycle 1	SMM	Asian	V3	PBMC	MRD negative	MRD negative	MRD negative			
CRDS-MC-0909-001	SMM-048	Cycle 4	SMM	Asian	V3	PBMC	MRD negative	MRD negative	MRD negative			
CRDS-MC-0831-001	SMM-049	Baseline	SMM	Black or African American	V3	PBMC	MRD negative	MRD negative	MRD negative			
CRDS-MC-0847-001	SMM-049	Cycle 1	SMM	Black or African American	V3	PBMC	MRD negative	MRD negative	MRD negative			
CRDS-MC-0914-001	SMM-049	Cycle 4	SMM	Black or African American	V3	PBMC	MRD negative	MRD negative	MRD negative			
CRDS-MC-0855-001	SMM-050	Baseline	SMM	Caucasian	V3	PBMC	MRD positive	MRD positive	MRD positive			
CRDS-MC-0868-001	SMM-050	Cycle 1	SMM	Caucasian	V3	PBMC	MRD positive	MRD positive	MRD positive			
CRDS-MC-0943-001	SMM-050	Cycle 4	SMM	Caucasian	V3	PBMC	MRD positive	MRD positive	MRD positive			
CRDS-MC-0860-001	SMM-051	Baseline	SMM	Hispanic	V3	PBMC	MRD negative	MRD negative	MRD negative			
CRDS-MC-0867-001	SMM-051	Cycle 1	SMM	Hispanic	V3	PBMC	MRD negative	MRD negative	MRD negative			
CRDS-MC-0942-001	SMM-051	Cycle 4	SMM	Hispanic	V3	PBMC	MRD negative	MRD negative	MRD negative			
CRDS-MC-0884-001	SMM-052	Baseline	SMM	Caucasian	V3	PBMC	MRD negative	MRD negative	MRD positive			
CRDS-MC-0889-001	SMM-052	Cycle 1	SMM	Caucasian	V3	PBMC	MRD negative	MRD negative	MRD positive			
CRDS-MC-0887-001	SMM-053	Baseline	SMM	Caucasian	V3	PBMC	MRD negative	MRD negative	MRD negative			
CRDS-MC-0902-001	SMM-053	Cycle 1	SMM	Caucasian	V3	PBMC	MRD negative	MRD negative	MRD negative			
CRDS-MC-0895-001	SMM-054	Baseline	SMM	Caucasian	V3	PBMC	MRD positive	MRD positive	MRD positive			
CRDS-MC-0913-001	SMM-054	Cycle 1	SMM	Caucasian	V3	PBMC	MRD positive	MRD positive	MRD positive			
CRDS-MC-0911-001	SMM-055	Baseline	SMM	Caucasian	V3	PBMC	MRD negative	MRD negative	MRD negative			
CRDS-MC-0933-001	SMM-055	Cycle 1	SMM	Caucasian	V3	PBMC	MRD negative	MRD negative	MRD negative			
