# Supplementary material for: Machine learning reveals distinct T-cell receptor clusters in plasma cell dyscrasias compared to healthy controls
Source: PLoS One. 2025 Oct 27;20(10):e0334053. doi: 10.1371/journal.pone.0334053 (PMC12558469; doi:10.1371/journal.pone.0334053)
Supplement: S2 Table — Only healthy donors ≥ 40 years of age were used in the final analysis. (PDF) [file pone.0334053.s003.pdf]

S2 Table. Number of unique productive sequences, Gini coefficient, and downsampled Gini coefficient computed per sample.
Only healthy donors ≥ 40 years of age were used in the final analysis.

Sample	Patient	Time point	Diagnosis	Productive rearangements	Gini	Gini downsampled
Keck0003_MC1	Keck0003_MC1	Baseline	Healthy	233486	0.195278926	0.052781003
Keck0004_MC1	Keck0004_MC1	Baseline	Healthy	150298	0.192487269	0.078491228
Keck0005_MC1	Keck0005_MC1	Baseline	Healthy	155916	0.113849996	0.028964308
Keck0006_MC1	Keck0006_MC1	Baseline	Healthy	167665	0.192097408	0.061228451
Keck0008_MC1	Keck0008_MC1	Baseline	Healthy	158973	0.151807133	0.036582175
Keck0010_MC1	Keck0010_MC1	Baseline	Healthy	137541	0.158974903	0.043213043
Keck0011_MC1	Keck0011_MC1	Baseline	Healthy	115430	0.195250183	0.066437912
Keck0012_MC1	Keck0012_MC1	Baseline	Healthy	109005	0.1498059	0.056515922
Keck0013_MC1	Keck0013_MC1	Baseline	Healthy	265537	0.203774866	0.069402227
Keck0021_MC1	Keck0021_MC1	Baseline	Healthy	301733	0.150499754	0.027348884
Keck0024_MC1	Keck0024_MC1	Baseline	Healthy	108492	0.381615577	0.250667757
Keck0027_MC1	Keck0027_MC1	Baseline	Healthy	72648	0.183363609	0.092210916
Keck0030_MC1	Keck0030_MC1	Baseline	Healthy	138321	0.189413451	0.07244954
Keck0031_MC1	Keck0031_MC1	Baseline	Healthy	199184	0.142245038	0.031390492
Keck0033_MC1	Keck0033_MC1	Baseline	Healthy	130728	0.16717819	0.08320779
Keck0036_MC1	Keck0036_MC1	Baseline	Healthy	136956	0.295578495	0.177632255
Keck0037_MC1	Keck0037_MC1	Baseline	Healthy	229522	0.161378871	0.04697555
Keck0039_MC1	Keck0039_MC1	Baseline	Healthy	98082	0.208915954	0.082949848
Keck0040_MC1	Keck0040_MC1	Baseline	Healthy	182628	0.191545009	0.0662972
Keck0041_MC1	Keck0041_MC1	Baseline	Healthy	131898	0.331174428	0.166849515
Keck0044_MC1	Keck0044_MC1	Baseline	Healthy	166361	0.300681882	0.13483733
Keck0045_MC1	Keck0045_MC1	Baseline	Healthy	119965	0.233086981	0.10460874
Keck0046_MC1	Keck0046_MC1	Baseline	Healthy	131205	0.241555289	0.081973679
Keck0047_MC1	Keck0047_MC1	Baseline	Healthy	154968	0.197837244	0.079371347
Keck0048_MC1	Keck0048_MC1	Baseline	Healthy	175436	0.237849263	0.084461387
Keck0049_MC1	Keck0049_MC1	Baseline	Healthy	250438	0.233847433	0.052369942
Keck0050_MC1	Keck0050_MC1	Baseline	Healthy	138526	0.268442538	0.151449184
Keck0051_MC1	Keck0051_MC1	Baseline	Healthy	137727	0.424208673	0.252031694
Keck0052_MC1	Keck0052_MC1	Baseline	Healthy	44656	0.218999574	0.179655289
Keck0053_MC1	Keck0053_MC1	Baseline	Healthy	144959	0.185147842	0.077210418
Keck0054_MC1	Keck0054_MC1	Baseline	Healthy	252230	0.270797666	0.085189676
Keck0055_MC1	Keck0055_MC1	Baseline	Healthy	470781	0.434997195	0.203610636
Keck0056_MC1	Keck0056_MC1	Baseline	Healthy	292879	0.296283603	0.116542994
Keck0057_MC1	Keck0057_MC1	Baseline	Healthy	404155	0.438503785	0.231008382
Keck0058_MC1	Keck0058_MC1	Baseline	Healthy	245552	0.262263182	0.095612159
Keck0059_MC1	Keck0059_MC1	Baseline	Healthy	815687	0.726399999	0.545282831
Keck0062_MC1	Keck0062_MC1	Baseline	Healthy	355792	0.35268196	0.148601634
Keck0063_MC1	Keck0063_MC1	Baseline	Healthy	140377	0.251486892	0.095145813
Keck0064_MC1	Keck0064_MC1	Baseline	Healthy	257934	0.284245492	0.094882459
Keck0065_MC1	Keck0065_MC1	Baseline	Healthy	262260	0.338994649	0.131666052
Keck0066_MC1	Keck0066_MC1	Baseline	Healthy	204529	0.382253426	0.172844886
Keck0067_MC1	Keck0067_MC1	Baseline	Healthy	326492	0.537120738	0.353031137
Keck0068_MC1	Keck0068_MC1	Baseline	Healthy	333420	0.339074349	0.157172182
Keck0069_MC1	Keck0069_MC1	Baseline	Healthy	793040	0.295075172	0.051719659
Keck0070_MC1	Keck0070_MC1	Baseline	Healthy	794743	0.331666367	0.056713071
Keck0071_MC1	Keck0071_MC1	Baseline	Healthy	551562	0.419502685	0.099112151
Keck0072_MC1	Keck0072_MC1	Baseline	Healthy	300433	0.224785861	0.056289421
Keck0073_MC1	Keck0073_MC1	Baseline	Healthy	451095	0.42231666	0.25204674
Keck0074_MC1	Keck0074_MC1	Baseline	Healthy	369847	0.388742129	0.145137755
Keck0075_MC1	Keck0075_MC1	Baseline	Healthy	237650	0.241599818	0.071600423
Keck0076_MC1	Keck0076_MC1	Baseline	Healthy	565423	0.510364734	0.320889454
Keck0077_MC1	Keck0077_MC1	Baseline	Healthy	352482	0.286472578	0.104813271
Keck0078_MC1	Keck0078_MC1	Baseline	Healthy	310264	0.295316685	0.087784684
Keck0079_MC1	Keck0079_MC1	Baseline	Healthy	335235	0.412113233	0.158844182
Keck0080_MC1	Keck0080_MC1	Baseline	Healthy	577358	0.280321539	0.055146296
Keck0081_MC1	Keck0081_MC1	Baseline	Healthy	488096	0.418119884	0.153866423

S2 Table. Number of unique productive sequences, Gini coefficient, and downsampled Gini coefficient computed per sample.
Only healthy donors ≥ 40 years of age were used in the final analysis.

Sample	Patient	Time point	Diagnosis	Productive rearangements	Gini	Gini downsampled
Keck0082_MC1	Keck0082_MC1	Baseline	Healthy	593522	0.461611433	0.25698877
Keck0083_MC1	Keck0083_MC1	Baseline	Healthy	466294	0.31368112	0.109672573
Keck0084_MC1	Keck0084_MC1	Baseline	Healthy	726272	0.613845413	0.398701822
Keck0085_MC1	Keck0085_MC1	Baseline	Healthy	342181	0.395050278	0.17127444
Keck0086_MC1	Keck0086_MC1	Baseline	Healthy	273701	0.284258102	0.094706796
Keck0087_MC1	Keck0087_MC1	Baseline	Healthy	416270	0.371933709	0.172053998
Keck0088_MC1	Keck0088_MC1	Baseline	Healthy	368993	0.341898685	0.134743632
Keck0089_MC1	Keck0089_MC1	Baseline	Healthy	329804	0.357582231	0.173785876
Keck0091_MC1	Keck0091_MC1	Baseline	Healthy	378284	0.401847078	0.174961512
Keck0093_MC1	Keck0093_MC1	Baseline	Healthy	706743	0.35197556	0.107742143
Keck0094_MC1	Keck0094_MC1	Baseline	Healthy	592217	0.329412724	0.099677643
Keck0095_MC1	Keck0095_MC1	Baseline	Healthy	696203	0.293254563	0.059327789
Keck0096_MC1	Keck0096_MC1	Baseline	Healthy	632231	0.49243396	0.236538229
Keck0101_MC1	Keck0101_MC1	Baseline	Healthy	745814	0.541032605	0.316766463
Keck0102_MC1	Keck0102_MC1	Baseline	Healthy	297642	0.297985481	0.097576367
Keck0103_MC1	Keck0103_MC1	Baseline	Healthy	239924	0.321090866	0.117627021
Keck0104_MC1	Keck0104_MC1	Baseline	Healthy	556400	0.311482545	0.065920008
Keck0105_MC1	Keck0105_MC1	Baseline	Healthy	405013	0.286386631	0.078159047
Keck0106_MC1	Keck0106_MC1	Baseline	Healthy	347857	0.359619107	0.102401737
Keck0108_MC1	Keck0108_MC1	Baseline	Healthy	254094	0.432825888	0.23402522
Keck0109_MC1	Keck0109_MC1	Baseline	Healthy	250526	0.35744791	0.159704933
Keck0110_MC1	Keck0110_MC1	Baseline	Healthy	285702	0.319861391	0.155350713
Keck0112_MC1	Keck0112_MC1	Baseline	Healthy	306424	0.386545016	0.183794584
Keck0115_MC1	Keck0115_MC1	Baseline	Healthy	556291	0.554596162	0.372019636
Keck0117_MC1	Keck0117_MC1	Baseline	Healthy	513475	0.480519806	0.241497104
Keck0118_MC1	Keck0118_MC1	Baseline	Healthy	660975	0.534858953	0.292716153
Keck0119_MC1	Keck0119_MC1	Baseline	Healthy	487792	0.304504099	0.077726042
Keck0120_MC1	Keck0120_MC1	Baseline	Healthy	474402	0.331649939	0.083818653
Subject_100	Subject_100	Baseline	Healthy	205508	0.503840874	0.333582159
Subject_101	Subject_101	Baseline	Healthy	216220	0.339312233	0.115374806
Subject_102	Subject_102	Baseline	Healthy	217972	0.260853729	0.090797213
Subject_103	Subject_103	Baseline	Healthy	156010	0.191895352	0.069127404
Subject_104	Subject_104	Baseline	Healthy	249592	0.163352926	0.037989138
Subject_105	Subject_105	Baseline	Healthy	143785	0.353356957	0.196495528
Subject_106	Subject_106	Baseline	Healthy	145510	0.368577149	0.200240643
Subject_12	Subject_12	Baseline	Healthy	214879	0.172181666	0.032318643
Subject_13	Subject_13	Baseline	Healthy	238451	0.352459188	0.219817354
Subject_14	Subject_14	Baseline	Healthy	72097	0.355707052	0.299768193
Subject_15	Subject_15	Baseline	Healthy	172132	0.194242591	0.086162746
Subject_151	Subject_151	Baseline	Healthy	57086	0.188658481	0.104946117
Subject_152	Subject_152	Baseline	Healthy	124207	0.199354279	0.055370934
Subject_153	Subject_153	Baseline	Healthy	78736	0.184146954	0.090966252
Subject_154	Subject_154	Baseline	Healthy	69860	0.106855357	0.036039157
Subject_155	Subject_155	Baseline	Healthy	71996	0.180684412	0.09782352
Subject_156	Subject_156	Baseline	Healthy	70505	0.127680095	0.059605271
Subject_157	Subject_157	Baseline	Healthy	43555	0.195497406	0.140751864
Subject_158	Subject_158	Baseline	Healthy	100817	0.161315746	0.06589817
Subject_16	Subject_16	Baseline	Healthy	121347	0.28529111	0.175235923
Subject_18	Subject_18	Baseline	Healthy	256228	0.296291123	0.074238324
Subject_22	Subject_22	Baseline	Healthy	106633	0.199803318	0.105377635
Subject_24	Subject_24	Baseline	Healthy	145333	0.215663022	0.092613872
Subject_26	Subject_26	Baseline	Healthy	112930	0.261657252	0.110629365
Subject_27	Subject_27	Baseline	Healthy	209045	0.139215335	0.03594515
Subject_39	Subject_39	Baseline	Healthy	118623	0.178703399	0.064946783
Subject_40	Subject_40	Baseline	Healthy	242208	0.356282768	0.244092247
Subject_41	Subject_41	Baseline	Healthy	179008	0.263155719	0.084615324

S2 Table. Number of unique productive sequences, Gini coefficient, and downsampled Gini coefficient computed per sample.
Only healthy donors ≥ 40 years of age were used in the final analysis.

Sample	Patient	Time point	Diagnosis	Productive rearangements	Gini	Gini downsampled
Subject_42	Subject_42	Baseline	Healthy	212871	0.396937069	0.253649418
Subject_44	Subject_44	Baseline	Healthy	187785	0.201138627	0.056324834
Subject_45	Subject_45	Baseline	Healthy	152684	0.27955575	0.100497822
Subject_46	Subject_46	Baseline	Healthy	183577	0.362253448	0.141395939
Subject_47	Subject_47	Baseline	Healthy	273714	0.143609042	0.031474715
Subject_48	Subject_48	Baseline	Healthy	210542	0.477021746	0.265005531
Subject_49	Subject_49	Baseline	Healthy	209198	0.46553685	0.312727422
Subject_50	Subject_50	Baseline	Healthy	260656	0.516162261	0.311525841
Subject_51	Subject_51	Baseline	Healthy	103469	0.413925657	0.224200675
Subject_52	Subject_52	Baseline	Healthy	272565	0.38622661	0.201824492
Subject_53	Subject_53	Baseline	Healthy	245984	0.229563721	0.06790311
Subject_54	Subject_54	Baseline	Healthy	269762	0.237407701	0.050657141
Subject_55	Subject_55	Baseline	Healthy	244328	0.215730455	0.087486945
Subject_56	Subject_56	Baseline	Healthy	259819	0.209292792	0.046818051
Subject_57	Subject_57	Baseline	Healthy	151627	0.352638711	0.195944005
Subject_58	Subject_58	Baseline	Healthy	231343	0.236999885	0.134171966
Subject_59	Subject_59	Baseline	Healthy	269783	0.219820086	0.072593689
Subject_60	Subject_60	Baseline	Healthy	204343	0.405507299	0.254760275
Subject_62	Subject_62	Baseline	Healthy	187952	0.218013544	0.07133663
Subject_63	Subject_63	Baseline	Healthy	183204	0.484440315	0.341321866
Subject_64	Subject_64	Baseline	Healthy	187864	0.567110465	0.40900931
Subject_65	Subject_65	Baseline	Healthy	184915	0.236156201	0.077357059
Subject_66	Subject_66	Baseline	Healthy	152255	0.285704159	0.133349782
Subject_67	Subject_67	Baseline	Healthy	154520	0.433433936	0.278836397
Subject_68	Subject_68	Baseline	Healthy	148051	0.348409407	0.236404444
Subject_69	Subject_69	Baseline	Healthy	199531	0.273239602	0.083789016
Subject_70	Subject_70	Baseline	Healthy	239034	0.278911789	0.089331462
Subject_71	Subject_71	Baseline	Healthy	212135	0.452445316	0.237096022
Subject_72	Subject_72	Baseline	Healthy	246418	0.364735349	0.19829125
Subject_73	Subject_73	Baseline	Healthy	240727	0.314450739	0.175799635
Subject_74	Subject_74	Baseline	Healthy	175256	0.35171749	0.197600852
Subject_75	Subject_75	Baseline	Healthy	284537	0.383482876	0.219035117
Subject_76	Subject_76	Baseline	Healthy	181330	0.23628322	0.075844706
Subject_77	Subject_77	Baseline	Healthy	209283	0.189717772	0.060132781
Subject_79	Subject_79	Baseline	Healthy	277894	0.211469709	0.04348943
Subject_80	Subject_80	Baseline	Healthy	89535	0.296425846	0.151926259
Subject_81	Subject_81	Baseline	Healthy	113323	0.345053857	0.164480581
Subject_82	Subject_82	Baseline	Healthy	160203	0.422935925	0.303366128
Subject_83	Subject_83	Baseline	Healthy	235761	0.397239443	0.209259098
Subject_84	Subject_84	Baseline	Healthy	129334	0.302917935	0.168797385
Subject_85	Subject_85	Baseline	Healthy	205256	0.197513591	0.083222051
Subject_86	Subject_86	Baseline	Healthy	181708	0.355188697	0.190503722
Subject_88	Subject_88	Baseline	Healthy	175088	0.530080105	0.345852446
Subject_89	Subject_89	Baseline	Healthy	150622	0.243371708	0.103582929
Subject_90	Subject_90	Baseline	Healthy	180941	0.304308633	0.157828503
Subject_91	Subject_91	Baseline	Healthy	223671	0.434850531	0.192297775
Subject_92	Subject_92	Baseline	Healthy	226111	0.404953213	0.263649967
Subject_93	Subject_93	Baseline	Healthy	237507	0.432734236	0.236380629
Subject_94	Subject_94	Baseline	Healthy	197183	0.300226746	0.104797928
Subject_95	Subject_95	Baseline	Healthy	233353	0.528596021	0.337511057
Subject_96	Subject_96	Baseline	Healthy	183242	0.726696596	0.60770042
Subject_97	Subject_97	Baseline	Healthy	154520	0.306133746	0.100127438
Subject_98	Subject_98	Baseline	Healthy	373319	0.633042006	0.518293576
Subject_99	Subject_99	Baseline	Healthy	294255	0.334374049	0.136395506
NHMM-MC-0781-001	MGUS-001	Baseline	MGUS	213497	0.472678741	0.269313419
NHMM-MC-1025-001	MGUS-001	Follow-up	MGUS	109443	0.351845186	0.20851099

S2 Table. Number of unique productive sequences, Gini coefficient, and downsampled Gini coefficient computed per sample.
Only healthy donors ≥ 40 years of age were used in the final analysis.

Sample	Patient	Time point	Diagnosis	Productive rearangements	Gini	Gini downsampled
NHMM-MC-0159-001	MGUS-002	Baseline	MGUS	324626	0.431941749	0.281380265
NHMM-MC-0839-002	MGUS-002	Follow-up	MGUS	183470	0.291174398	0.169637033
NHMM-MC-0238-001	MGUS-003	Baseline	MGUS	102788	0.366344637	0.24209659
NHMM-MC-0678-001	MGUS-003	Follow-up	MGUS	102870	0.312015795	0.181960529
NHMM-MC-0043-001	MGUS-004	Baseline	MGUS	330944	0.305223095	0.162439524
NHMM-MC-0666-001	MGUS-004	Follow-up	MGUS	209878	0.280382526	0.180241974
NHMM-MC-0882-001	MGUS-005	Baseline	MGUS	76549	0.568502793	0.477544569
NHMM-MC-0966-001	MGUS-005	Follow-up	MGUS	132319	0.599725504	0.491825427
NHMM-MC-0143-001	MGUS-006	Baseline	MGUS	203952	0.273232435	0.084350674
NHMM-MC-0141-001	MGUS-007	Baseline	MGUS	238122	0.450946892	0.200582338
NHMM-MC-0280-001	MGUS-008	Baseline	MGUS	36263	0.328463926	0.232092789
NHMM-MC-0921-002	MGUS-008	Follow-up	MGUS	278231	0.358627807	0.154787758
NHMM-MC-0226-001	MGUS-009	Baseline	MGUS	324396	0.694292285	0.534616775
NHMM-MC-0866-001	MGUS-009	Follow-up	MGUS	228148	0.578588329	0.432065408
NHMM-MC-0005-001	MGUS-010	Baseline	MGUS	100253	0.357852896	0.185508969
NHMM-MC-0946-001	MGUS-010	Follow-up	MGUS	197750	0.372929078	0.164800305
NHMM-MC-1021-001	MGUS-011	Baseline	MGUS	10833	0.239897717	0.239897717
NHMM-MC-0936-001	MGUS-011	Follow-up	MGUS	117969	0.652943461	0.545538399
NHMM-MC-0041-001	MGUS-012	Baseline	MGUS	474819	0.556239266	0.224498083
NHMM-MC-0791-001	MGUS-012	Follow-up	MGUS	290888	0.470841227	0.230064004
NHMM-MC-0504-001	MGUS-013	Baseline	MGUS	288438	0.506177502	0.321996195
NHMM-MC-0986-001	MGUS-013	Follow-up	MGUS	195597	0.352764703	0.21974344
NHMM-MC-0189-001	MGUS-014	Baseline	MGUS	159345	0.486094445	0.338412824
NHMM-MC-0858-002	MGUS-014	Follow-up	MGUS	92249	0.423538068	0.321654227
NHMM-MC-0438-001	MGUS-015	Baseline	MGUS	409506	0.382931334	0.136045017
NHMM-MC-0968-001	MGUS-015	Follow-up	MGUS	73087	0.320252858	0.16906226
NHMM-MC-0167-001	MGUS-016	Baseline	MGUS	182218	0.499354549	0.329543418
NHMM-MC-0832-001	MGUS-016	Follow-up	MGUS	236119	0.376209748	0.212104317
NHMM-MC-0149-001	MGUS-017	Baseline	MGUS	374084	0.360059653	0.125050893
NHMM-MC-0824-001	MGUS-017	Follow-up	MGUS	175425	0.224214515	0.061204636
NHMM-MC-0125-001	MGUS-018	Baseline	MGUS	273418	0.48611375	0.173655722
NHMM-MC-0027-001	MGUS-019	Baseline	MGUS	242016	0.601134027	0.463896905
NHMM-MC-0869-002	MGUS-019	Follow-up	MGUS	242763	0.460138636	0.319372643
NHMM-MC-0877-001	MGUS-020	Baseline	MGUS	295385	0.243428857	0.064004521
NHMM-MC-0963-001	MGUS-020	Follow-up	MGUS	298163	0.198797062	0.053107186
NHMM-MC-0137-001	MGUS-021	Baseline	MGUS	358910	0.752811013	0.630114464
NHMM-MC-0191-001	MGUS-022	Baseline	MGUS	84767	0.375464833	0.242261019
NHMM-MC-0822-001	MGUS-022	Follow-up	MGUS	212856	0.333403377	0.164385902
NHMM-MC-0508-001	MGUS-023	Baseline	MGUS	224264	0.411225736	0.197926797
NHMM-MC-0723-001	MGUS-023	Follow-up	MGUS	232655	0.383383626	0.151879534
NHMM-MC-0023-001	MGUS-024	Baseline	MGUS	168743	0.298443877	0.103740552
NHMM-MC-0214-001	MGUS-024	Follow-up	MGUS	47212	0.208950818	0.105046056
NHMM-MC-0550-001	MGUS-025	Baseline	MGUS	342258	0.236171244	0.04037783
NHMM-MC-0837-001	MGUS-025	Follow-up	MGUS	154453	0.145842202	0.037648479
NHMM-MC-0208-001	MGUS-026	Baseline	MGUS	264047	0.346401791	0.128132429
NHMM-MC-0848-001	MGUS-026	Follow-up	MGUS	262533	0.265436784	0.090671176
NHMM-MC-0057-001	MGUS-027	Baseline	MGUS	254047	0.494983491	0.257829507
NHMM-MC-0452-001	MGUS-027	Follow-up	MGUS	193208	0.337325696	0.145790948
NHMM-MC-0320-001	MGUS-028	Baseline	MGUS	267303	0.406960258	0.241257166
NHMM-MC-0876-002	MGUS-028	Follow-up	MGUS	282943	0.476370584	0.346456864
NHMM-MC-0364-001	MGUS-029	Baseline	MGUS	162943	0.428485868	0.247613099
NHMM-MC-0942-001	MGUS-029	Follow-up	MGUS	296569	0.376041129	0.184344081
NHMM-MC-0029-001	MGUS-030	Baseline	MGUS	474983	0.482695193	0.25926287
NHMM-MC-0888-002	MGUS-030	Follow-up	MGUS	231246	0.430192073	0.291318294
NHMM-MC-0314-001	MGUS-031	Baseline	MGUS	71906	0.353124268	0.206815292
NHMM-MC-0047-001	MGUS-032	Baseline	MGUS	312619	0.454716634	0.282074187

S2 Table. Number of unique productive sequences, Gini coefficient, and downsampled Gini coefficient computed per sample.
Only healthy donors ≥ 40 years of age were used in the final analysis.

Sample	Patient	Time point	Diagnosis	Productive rearangements	Gini	Gini downsampled
NHMM-MC-0890-002	MGUS-032	Follow-up	MGUS	170183	0.314498538	0.192420434
NHMM-MC-0161-001	MGUS-033	Baseline	MGUS	245371	0.313789915	0.076609787
NHMM-MC-0760-001	MGUS-033	Follow-up	MGUS	277924	0.253619445	0.050148516
NHMM-MC-0013-001	MGUS-034	Baseline	MGUS	133363	0.568682708	0.390753221
NHMM-MC-0957-001	MGUS-034	Follow-up	MGUS	222778	0.546911853	0.33576168
NHMM-MC-0915-001	MGUS-035	Baseline	MGUS	372001	0.641244718	0.443898915
NHMM-MC-1049-001	MGUS-035	Follow-up	MGUS	29135	0.48560699	0.435099281
NHMM-MC-0031-001	MGUS-036	Baseline	MGUS	311953	0.389421236	0.13655416
NHMM-MC-0630-001	MGUS-036	Follow-up	MGUS	294853	0.370941975	0.130379066
NHMM-MC-0670-001	MGUS-038	Baseline	MGUS	444823	0.737967525	0.574265194
NHMM-MC-0382-001	MGUS-039	Baseline	MGUS	404371	0.344530012	0.133591091
NHMM-MC-0938-001	MGUS-039	Follow-up	MGUS	376501	0.305816637	0.123685486
NHMM-MC-0784-001	MGUS-040	Baseline	MGUS	234225	0.400856201	0.201955317
NHMM-MC-0964-001	MGUS-040	Follow-up	MGUS	290118	0.362885578	0.171489188
NHMM-MC-0276-001	MGUS-041	Baseline	MGUS	249932	0.4842939	0.215356763
NHMM-MC-0944-001	MGUS-041	Follow-up	MGUS	176050	0.389902024	0.16649927
NHMM-MC-0035-001	MGUS-042	Baseline	MGUS	340042	0.627239586	0.401266558
NHMM-MC-0962-002	MGUS-042	Follow-up	MGUS	193834	0.605833908	0.441678671
NHMM-MC-0017-001	MGUS-043	Baseline	MGUS	276583	0.729378158	0.625573091
NHMM-MC-0950-002	MGUS-043	Follow-up	MGUS	199425	0.714801073	0.620170405
NHMM-MC-0081-001	MGUS-044	Baseline	MGUS	64011	0.32304461	0.175136153
NHMM-MC-0426-001	MGUS-044	Follow-up	MGUS	206262	0.370632506	0.140503873
NHMM-MC-0061-001	MGUS-045	Baseline	MGUS	301481	0.564106001	0.376293746
NHMM-MC-0931-001	MGUS-045	Follow-up	MGUS	228344	0.390903297	0.247567839
NHMM-MC-0097-001	MGUS-046	Baseline	MGUS	386980	0.461197965	0.26856771
NHMM-MC-0352-001	MGUS-046	Follow-up	MGUS	235444	0.330783639	0.188327327
NHMM-MC-0021-001	MGUS-047	Baseline	MGUS	216287	0.555168958	0.311311045
NHMM-MC-0977-002	MGUS-047	Follow-up	MGUS	275889	0.510345278	0.267274431
NHMM-MC-0268-001	MGUS-048	Baseline	MGUS	97948	0.508819572	0.407420228
NHMM-MC-0893-001	MGUS-048	Follow-up	MGUS	269016	0.435655416	0.328059992
NHMM-MC-0780-001	MGUS-049	Baseline	MGUS	369492	0.253143666	0.064308574
NHMM-MC-0967-001	MGUS-049	Follow-up	MGUS	329102	0.166756324	0.031176563
NHMM-MC-0458-001	MGUS-050	Baseline	MGUS	241201	0.276769761	0.079526952
NHMM-MC-0813-001	MGUS-050	Follow-up	MGUS	53205	0.267994904	0.141647648
NHMM-MC-0049-001	MGUS-051	Baseline	MGUS	92002	0.222771935	0.070095028
NHMM-MC-0981-001	MGUS-051	Follow-up	MGUS	249702	0.165245646	0.029311681
NHMM-MC-0270-001	MGUS-052	Baseline	MGUS	70749	0.592620236	0.512728634
NHMM-MC-0898-001	MGUS-052	Follow-up	MGUS	162394	0.713560355	0.643350953
NHMM-MC-0272-001	MGUS-053	Baseline	MGUS	210447	0.437093743	0.260990511
NHMM-MC-0672-001	MGUS-053	Follow-up	MGUS	296804	0.387281516	0.183612506
NHMM-MC-0102-001	MGUS-054	Baseline	MGUS	43789	0.233798791	0.135229558
NHMM-MC-0296-001	MGUS-054	Follow-up	MGUS	169218	0.240734825	0.094643473
NHMM-MC-0484-001	MGUS-055	Baseline	MGUS	287013	0.300631671	0.093135182
NHMM-MC-0978-001	MGUS-055	Follow-up	MGUS	333076	0.205449109	0.047011243
NHMM-MC-0912-001	MGUS-056	Baseline	MGUS	208457	0.631659237	0.488639179
NHMM-MC-1047-001	MGUS-056	Follow-up	MGUS	26904	0.477129515	0.434108443
NHMM-MC-0715-001	MGUS-057	Baseline	MGUS	667654	0.723350855	0.594209685
NHMM-MC-0902-001	MGUS-057	Follow-up	MGUS	276350	0.549154638	0.43113847
NHMM-MC-0011-001	MGUS-058	Baseline	MGUS	228858	0.485501515	0.22858076
NHMM-MC-0386-001	MGUS-058	Follow-up	MGUS	170046	0.46621781	0.257936907
NHMM-MC-0412-001	MGUS-059	Baseline	MGUS	392207	0.236236516	0.037234099
NHMM-MC-0768-002	MGUS-059	Follow-up	MGUS	243627	0.140176669	0.017587998
NHMM-MC-0454-001	MGUS-060	Baseline	MGUS	258791	0.322984243	0.120952411
NHMM-MC-0829-001	MGUS-060	Follow-up	MGUS	292392	0.270164132	0.086950175
NHMM-MC-0171-001	MGUS-061	Baseline	MGUS	72860	0.397279053	0.222986493
NHMM-MC-0985-002	MGUS-061	Follow-up	MGUS	142999	0.360871906	0.168849224

S2 Table. Number of unique productive sequences, Gini coefficient, and downsampled Gini coefficient computed per sample.
Only healthy donors ≥ 40 years of age were used in the final analysis.

Sample	Patient	Time point	Diagnosis	Productive rearangements	Gini	Gini downsampled
NHMM-MC-0400-001	MGUS-062	Baseline	MGUS	236350	0.347047787	0.144246356
NHMM-MC-0958-001	MGUS-062	Follow-up	MGUS	288498	0.266001008	0.091718135
NHMM-MC-0586-001	MGUS-063	Baseline	MGUS	287472	0.468173016	0.260071015
NHMM-MC-0889-001	MGUS-063	Follow-up	MGUS	247832	0.546312495	0.393215361
NHMM-MC-0099-001	MGUS-064	Baseline	MGUS	299824	0.540566812	0.358578975
NHMM-MC-0807-001	MGUS-065	Baseline	MGUS	279673	0.25287839	0.107102235
NHMM-MC-0982-002	MGUS-065	Follow-up	MGUS	189303	0.220285453	0.088408244
NHMM-MC-0448-001	MGUS-066	Baseline	MGUS	248549	0.412363982	0.240376176
NHMM-MC-0965-001	MGUS-066	Follow-up	MGUS	238965	0.35005044	0.197656345
NHMM-MC-0258-001	MGUS-067	Baseline	MGUS	252777	0.239288302	0.082141486
NHMM-MC-0674-001	MGUS-067	Follow-up	MGUS	208672	0.178605526	0.053644394
NHMM-MC-0642-001	MGUS-068	Baseline	MGUS	217678	0.46623234	0.317277978
NHMM-MC-0975-001	MGUS-068	Follow-up	MGUS	222745	0.385936709	0.216676987
NHMM-MC-0901-001	MGUS-069	Baseline	MGUS	300133	0.223092619	0.068816705
NHMM-MC-0632-001	MGUS-070	Baseline	MGUS	243326	0.413949523	0.148757417
NHMM-MC-0816-001	MGUS-070	Follow-up	MGUS	227482	0.345956406	0.115433756
NHMM-MC-0916-001	MGUS-071	Baseline	MGUS	285817	0.50295185	0.321234327
NHMM-MC-0988-001	MGUS-071	Follow-up	MGUS	233473	0.374970138	0.236479598
NHMM-MC-0131-001	MGUS-072	Baseline	MGUS	141796	0.450098904	0.306212667
NHMM-MC-0989-001	MGUS-072	Follow-up	MGUS	257566	0.327816991	0.212971969
NHMM-MC-0894-002	MGUS-073	Baseline	MGUS	668970	0.58711292	0.298825939
NHMM-MC-1056-001	MGUS-073	Follow-up	MGUS	55698	0.473453253	0.372163774
NHMM-MC-0109-001	MGUS-074	Baseline	MGUS	74970	0.371667232	0.227824564
NHMM-MC-0526-001	MGUS-074	Follow-up	MGUS	195073	0.346948615	0.156076353
NHMM-MC-0157-001	MGUS-075	Baseline	MGUS	63338	0.225003829	0.084212535
NHMM-MC-0840-001	MGUS-075	Follow-up	MGUS	125229	0.240974429	0.075009838
NHMM-MC-0440-001	MGUS-076	Baseline	MGUS	84632	0.381543244	0.237494403
NHMM-MC-0960-001	MGUS-076	Follow-up	MGUS	230402	0.427913234	0.226849892
NHMM-MC-0145-001	MGUS-077	Baseline	MGUS	238792	0.340576947	0.153974908
NHMM-MC-0830-001	MGUS-077	Follow-up	MGUS	212847	0.253862878	0.103661139
NHMM-MC-0552-001	MGUS-078	Baseline	MGUS	393317	0.409929006	0.192823438
NHMM-MC-0853-001	MGUS-078	Follow-up	MGUS	54843	0.358965422	0.23591632
NHMM-MC-0362-001	MGUS-079	Baseline	MGUS	142184	0.490088824	0.36269446
NHMM-MC-0920-001	MGUS-079	Follow-up	MGUS	248034	0.49481869	0.372694252
NHMM-MC-0169-001	MGUS-080	Baseline	MGUS	91344	0.233388974	0.098572771
NHMM-MC-0856-001	MGUS-080	Follow-up	MGUS	247625	0.208578485	0.064752346
NHMM-MC-0054-001	MGUS-081	Baseline	MGUS	59546	0.22132423	0.097650811
NHMM-MC-0648-001	MGUS-081	Follow-up	MGUS	250245	0.215401524	0.050957229
MM-PBMC-256	MM-001	Baseline	MM	91243	0.195737163	0.062949369
MM-PBMC-234	MM-002	Baseline	MM	108728	0.181957508	0.081611978
MM-PBMC-328	MM-003	Baseline	MM	195430	0.320149804	0.172358483
MM-PBMC-347	MM-004	Baseline	MM	123659	0.571107017	0.446912745
MM-PBMC-339	MM-005	Baseline	MM	162232	0.414895372	0.267168524
MM-PBMC-355	MM-006	Baseline	MM	177633	0.466094535	0.348939676
MM-PBMC-364	MM-007	Baseline	MM	177381	0.362097153	0.238528051
MM-PBMC-388	MM-008	Baseline	MM	303312	0.499564198	0.368595429
MM-PBMC-386	MM-009	Baseline	MM	166699	0.190219782	0.044500683
MM-PBMC-395	MM-010	Baseline	MM	167536	0.293486001	0.160553637
MM-PBMC-014	MM-011	Baseline	MM	206789	0.282386039	0.063810402
MM-PBMC-116	MM-012	Baseline	MM	190765	0.455553929	0.303460851
MM-PBMC-174	MM-013	Baseline	MM	145884	0.290683017	0.133617597
MM-PBMC-188	MM-014	Baseline	MM	170358	0.742215655	0.638910092
MM-PBMC-426	MM-015	Baseline	MM	250935	0.365923515	0.211847548
MM-PBMC-441	MM-016	Baseline	MM	234901	0.393163034	0.211853464
MM-PBMC-452	MM-017	Baseline	MM	217296	0.268044479	0.08890465
MM-PBMC-474	MM-018	Baseline	MM	390130	0.53534485	0.368355591

S2 Table. Number of unique productive sequences, Gini coefficient, and downsampled Gini coefficient computed per sample.
Only healthy donors ≥ 40 years of age were used in the final analysis.

Sample	Patient	Time point	Diagnosis	Productive rearangements	Gini	Gini downsampled
MM-PBMC-476	MM-019	Baseline	MM	221662	0.70009607	0.55774088
MM-PBMC-496	MM-020	Baseline	MM	318892	0.699148564	0.626818958
MM-PBMC-491	MM-021	Baseline	MM	259698	0.324447714	0.108659554
MM-PBMC-509	MM-022	Baseline	MM	298474	0.540911312	0.381414868
MM-PBMC-513	MM-023	Baseline	MM	326453	0.413284248	0.143635919
MM-PBMC-524	MM-024	Baseline	MM	260725	0.731055262	0.591353223
MM-PBMC-566	MM-025	Baseline	MM	267380	0.60723301	0.481628935
MM-PBMC-600	MM-026	Baseline	MM	236344	0.375270848	0.127390826
MM-PBMC-621	MM-027	Baseline	MM	186670	0.263213615	0.09229919
MM-PBMC-653	MM-028	Baseline	MM	221830	0.324916645	0.103588151
MM-PBMC-666	MM-029	Baseline	MM	277539	0.417966714	0.185326671
MM-PBMC-697	MM-030	Baseline	MM	126609	0.271903034	0.158127061
MM-PBMC-707	MM-031	Baseline	MM	242571	0.467042678	0.323240686
SMM-PBMC-001	SMM-001	Baseline	SMM	167070	0.263881344	0.115434186
CRDS-MC-0017-001	SMM-001	Cycle 1	SMM	58033	0.172898187	0.080077949
SMM-PBMC-275	SMM-001	Cycle 32	SMM	152277	0.347331477	0.199802122
CRDS-MC-0048-001	SMM-001	Cycle 4	SMM	128168	0.252970546	0.122006806
SMM-PBMC-068	SMM-001	Cycle 8	SMM	207242	0.299586832	0.122823497
SMM-PBMC-008	SMM-002	Baseline	SMM	177447	0.50289685	0.29125547
CRDS-MC-0019-001	SMM-002	Cycle 1	SMM	53742	0.402280928	0.285742038
SMM-PBMC-196	SMM-002	Cycle 20	SMM	144060	0.491638933	0.277002259
SMM-PBMC-336	SMM-002	Cycle 32	SMM	134277	0.474116973	0.273878775
CRDS-MC-0050-001	SMM-002	Cycle 4	SMM	40120	0.349955644	0.251496144
SMM-PBMC-087	SMM-002	Cycle 8	SMM	79468	0.387742807	0.214820773
SMM-PBMC-023	SMM-003	Baseline	SMM	179526	0.231357758	0.072088964
CRDS-MC-0043-001	SMM-003	Cycle 1	SMM	61996	0.149244637	0.059537563
SMM-PBMC-221	SMM-003	Cycle 20	SMM	137174	0.20789085	0.058050144
SMM-PBMC-389	SMM-003	Cycle 32	SMM	141685	0.220461051	0.064602321
CRDS-MC-0061-001	SMM-003	Cycle 4	SMM	178033	0.161744362	0.035992308
SMM-PBMC-100	SMM-003	Cycle 8	SMM	118088	0.199844893	0.052102984
SMM-PBMC-033	SMM-004	Baseline	SMM	314303	0.499510867	0.221287932
CRDS-MC-0046-001	SMM-004	Cycle 1	SMM	102010	0.320018387	0.142849969
SMM-PBMC-227	SMM-004	Cycle 20	SMM	212118	0.413153861	0.150163077
SMM-PBMC-395	SMM-004	Cycle 32	SMM	202550	0.440860686	0.182100612
CRDS-MC-0069-001	SMM-004	Cycle 4	SMM	192854	0.344716122	0.116672638
SMM-PBMC-108	SMM-004	Cycle 8	SMM	167343	0.348722871	0.137467436
SMM-PBMC-054	SMM-005	Baseline	SMM	158632	0.256883553	0.0860528
CRDS-MC-0062-001	SMM-005	Cycle 1	SMM	132518	0.186929742	0.054620575
SMM-PBMC-255	SMM-005	Cycle 20	SMM	126273	0.271179546	0.086369715
SMM-PBMC-407	SMM-005	Cycle 32	SMM	186336	0.313842401	0.095765569
CRDS-MC-0091-001	SMM-005	Cycle 4	SMM	137802	0.177614935	0.044253535
SMM-PBMC-143	SMM-005	Cycle 8	SMM	122524	0.307071184	0.147731039
SMM-PBMC-063	SMM-006	Baseline	SMM	217632	0.46025798	0.238512991
CRDS-MC-0072-001	SMM-006	Cycle 1	SMM	155168	0.343221094	0.158711231
SMM-PBMC-265	SMM-006	Cycle 20	SMM	198655	0.454760858	0.207858792
SMM-PBMC-412	SMM-006	Cycle 32	SMM	175596	0.462535792	0.239648592
CRDS-MC-0111-001	SMM-006	Cycle 4	SMM	133059	0.350641415	0.155607059
SMM-PBMC-153	SMM-006	Cycle 8	SMM	163654	0.410316236	0.202808794
SMM-PBMC-073	SMM-007	Baseline	SMM	280489	0.252796504	0.049040242
CRDS-MC-0089-001	SMM-007	Cycle 1	SMM	185703	0.204388393	0.050357147
SMM-PBMC-277	SMM-007	Cycle 20	SMM	246699	0.297987194	0.072976075
SMM-PBMC-415	SMM-007	Cycle 32	SMM	211483	0.270192644	0.064958812
CRDS-MC-0126-001	SMM-007	Cycle 4	SMM	192410	0.167332789	0.039537823
SMM-PBMC-159	SMM-007	Cycle 8	SMM	244751	0.255070945	0.054162131
SMM-PBMC-074	SMM-008	Baseline	SMM	299466	0.40116491	0.22392124
CRDS-MC-0088-001	SMM-008	Cycle 1	SMM	210902	0.28894188	0.12962123

S2 Table. Number of unique productive sequences, Gini coefficient, and downsampled Gini coefficient computed per sample.
Only healthy donors ≥ 40 years of age were used in the final analysis.

Sample	Patient	Time point	Diagnosis	Productive rearangements	Gini	Gini downsampled
SMM-PBMC-273	SMM-008	Cycle 20	SMM	261033	0.529005151	0.344744962
SMM-PBMC-416	SMM-008	Cycle 32	SMM	261138	0.549716162	0.368347202
CRDS-MC-0119-001	SMM-008	Cycle 4	SMM	133609	0.28444844	0.161228446
SMM-PBMC-157	SMM-008	Cycle 8	SMM	107752	0.582733225	0.428337034
SMM-PBMC-096	SMM-009	Baseline	SMM	233391	0.377639536	0.170693719
CRDS-MC-0110-001	SMM-009	Cycle 1	SMM	82160	0.223400081	0.112421389
CRDS-MC-0268-001	SMM-009	Cycle 20	SMM	69353	0.152445904	0.063810619
CRDS-MC-0146-001	SMM-009	Cycle 4	SMM	116898	0.317352906	0.189231344
SMM-PBMC-173	SMM-009	Cycle 8	SMM	105114	0.308911759	0.158465252
SMM-PBMC-103	SMM-010	Baseline	SMM	285139	0.281953695	0.068203521
CRDS-MC-0113-001	SMM-010	Cycle 1	SMM	204352	0.191051934	0.046409154
SMM-PBMC-296	SMM-010	Cycle 20	SMM	135690	0.334272751	0.135781862
SMM-PBMC-425	SMM-010	Cycle 32	SMM	236558	0.372952783	0.127986335
CRDS-MC-0148-001	SMM-010	Cycle 4	SMM	98623	0.212537822	0.074068481
SMM-PBMC-177	SMM-010	Cycle 8	SMM	102598	0.270992536	0.10353949
SMM-PBMC-112	SMM-011	Baseline	SMM	172474	0.265780068	0.072071897
CRDS-MC-0129-001	SMM-011	Cycle 1	SMM	109849	0.181021636	0.048748626
SMM-PBMC-299	SMM-011	Cycle 20	SMM	216591	0.335423989	0.093488736
SMM-PBMC-427	SMM-011	Cycle 32	SMM	205417	0.364694374	0.108622714
CRDS-MC-0155-001	SMM-011	Cycle 4	SMM	76201	0.17393578	0.067033809
SMM-PBMC-184	SMM-011	Cycle 8	SMM	75441	0.266022102	0.123481357
SMM-PBMC-115	SMM-012	Baseline	SMM	189286	0.380131071	0.221714262
CRDS-MC-0132-001	SMM-012	Cycle 1	SMM	47134	0.180137952	0.094760413
SMM-PBMC-301	SMM-012	Cycle 20	SMM	117222	0.390557906	0.207612272
SMM-PBMC-429	SMM-012	Cycle 32	SMM	96992	0.39750122	0.238280334
CRDS-MC-0156-001	SMM-012	Cycle 4	SMM	45556	0.165567398	0.08612626
SMM-PBMC-187	SMM-012	Cycle 8	SMM	103783	0.404016636	0.272747282
SMM-PBMC-179	SMM-013	Baseline	SMM	186903	0.521192249	0.389321033
CRDS-MC-0185-001	SMM-013	Cycle 1	SMM	95311	0.39759675	0.304398577
SMM-PBMC-413	SMM-013	Cycle 20	SMM	153251	0.34243705	0.190251955
SMM-PBMC-446	SMM-013	Cycle 32	SMM	160973	0.339501039	0.208749697
CRDS-MC-0228-001	SMM-013	Cycle 4	SMM	127993	0.294835816	0.194367879
SMM-PBMC-267	SMM-013	Cycle 8	SMM	99695	0.348352431	0.256016253
SMM-PBMC-188	SMM-014	Baseline	SMM	283571	0.822912352	0.71334002
CRDS-MC-0195-001	SMM-014	Cycle 1	SMM	185874	0.621680414	0.502564632
SMM-PBMC-420	SMM-014	Cycle 20	SMM	175232	0.615767303	0.459493159
SMM-PBMC-440	SMM-014	Cycle 32	SMM	214073	0.616117919	0.461222203
CRDS-MC-0245-001	SMM-014	Cycle 4	SMM	146309	0.613920035	0.516651305
SMM-PBMC-288	SMM-014	Cycle 8	SMM	219815	0.652684352	0.425838688
SMM-PBMC-190	SMM-015	Baseline	SMM	244280	0.297799524	0.152725858
CRDS-MC-0197-001	SMM-015	Cycle 1	SMM	199084	0.195560601	0.0605816
SMM-PBMC-418	SMM-015	Cycle 20	SMM	205038	0.296306586	0.124511255
SMM-PBMC-438	SMM-015	Cycle 32	SMM	137460	0.322815965	0.156741885
CRDS-MC-0239-001	SMM-015	Cycle 4	SMM	191802	0.26465067	0.166921553
SMM-PBMC-279	SMM-015	Cycle 8	SMM	226379	0.278637576	0.117522997
SMM-PBMC-209	SMM-016	Baseline	SMM	248051	0.372960673	0.193808113
CRDS-MC-0216-001	SMM-016	Cycle 1	SMM	136371	0.314645176	0.161986224
SMM-PBMC-422	SMM-016	Cycle 20	SMM	174588	0.435140947	0.246428907
CRDS-MC-0249-001	SMM-016	Cycle 4	SMM	172643	0.371943866	0.201116748
SMM-PBMC-291	SMM-016	Cycle 8	SMM	140302	0.40495761	0.245841631
SMM-PBMC-201	SMM-017	Baseline	SMM	230277	0.202083857	0.032856792
CRDS-MC-0220-001	SMM-017	Cycle 1	SMM	180634	0.145091616	0.025830848
SMM-PBMC-421	SMM-017	Cycle 20	SMM	216971	0.301289813	0.109044121
SMM-PBMC-441	SMM-017	Cycle 32	SMM	178917	0.279213316	0.068972914
CRDS-MC-0248-001	SMM-017	Cycle 4	SMM	129213	0.172793011	0.048122291
SMM-PBMC-290	SMM-017	Cycle 8	SMM	76861	0.159157526	0.044028813

S2 Table. Number of unique productive sequences, Gini coefficient, and downsampled Gini coefficient computed per sample.
Only healthy donors ≥ 40 years of age were used in the final analysis.

Sample	Patient	Time point	Diagnosis	Productive rearangements	Gini	Gini downsampled
SMM-PBMC-203	SMM-018	Baseline	SMM	223629	0.324959324	0.102087641
CRDS-MC-0219-001	SMM-018	Cycle 1	SMM	232185	0.295362686	0.090006288
SMM-PBMC-424	SMM-018	Cycle 20	SMM	234867	0.625332241	0.473490052
SMM-PBMC-443	SMM-018	Cycle 32	SMM	201109	0.567443587	0.416959876
CRDS-MC-0250-001	SMM-018	Cycle 4	SMM	171448	0.218433498	0.069991719
SMM-PBMC-289	SMM-018	Cycle 8	SMM	124268	0.405243029	0.236913764
SMM-PBMC-459	SMM-019	Baseline	SMM	283739	0.261712925	0.079564981
CRDS-MC-0463-001	SMM-019	Cycle 1	SMM	156629	0.178689672	0.042152431
CRDS-MC-0482-001	SMM-019	Cycle 20	SMM	358374	0.242730286	0.045933414
CRDS-MC-0501-001	SMM-019	Cycle 32	SMM	199487	0.187483255	0.033350165
CRDS-MC-0467-001	SMM-019	Cycle 4	SMM	279845	0.200308794	0.027129938
CRDS-MC-0474-001	SMM-019	Cycle 8	SMM	180712	0.188571887	0.036505201
SMM-PBMC-484	SMM-020	Baseline	SMM	171439	0.600337913	0.513502568
CRDS-MC-0497-001	SMM-020	Cycle 1	SMM	208528	0.347609568	0.215171363
CRDS-MC-0801-001	SMM-020	Cycle 20	SMM	96481	0.433822784	0.323944671
CRDS-MC-0949-001	SMM-020	Cycle 32	SMM	128364	0.588550968	0.468275509
CRDS-MC-0539-001	SMM-020	Cycle 4	SMM	280764	0.361240105	0.179964733
CRDS-MC-0614-001	SMM-020	Cycle 8	SMM	146693	0.427897719	0.285602312
SMM-PBMC-488	SMM-021	Baseline	SMM	247699	0.494936563	0.355030766
CRDS-MC-0498-001	SMM-021	Cycle 1	SMM	281591	0.356736808	0.20396083
CRDS-MC-0791-001	SMM-021	Cycle 20	SMM	159067	0.390134782	0.264819814
CRDS-MC-0534-001	SMM-021	Cycle 4	SMM	226505	0.42593835	0.308623702
CRDS-MC-0604-001	SMM-021	Cycle 8	SMM	252105	0.530359525	0.418322594
SMM-PBMC-461	SMM-022	Baseline	SMM	199378	0.416889449	0.259203535
CRDS-MC-0465-001	SMM-022	Cycle 1	SMM	243666	0.368927357	0.188639979
CRDS-MC-0768-001	SMM-022	Cycle 20	SMM	75235	0.342963574	0.215961383
CRDS-MC-0982-001	SMM-022	Cycle 32	SMM	97736	0.436648775	0.287054403
CRDS-MC-0487-001	SMM-022	Cycle 4	SMM	278508	0.324599458	0.153870239
CRDS-MC-0572-001	SMM-022	Cycle 8	SMM	44855	0.252810277	0.181941727
SMM-PBMC-473	SMM-023	Baseline	SMM	236950	0.682738615	0.565873811
CRDS-MC-0478-001	SMM-023	Cycle 1	SMM	229666	0.491956617	0.346109663
CRDS-MC-0764-001	SMM-023	Cycle 20	SMM	169648	0.469401048	0.335731227
CRDS-MC-0898-001	SMM-023	Cycle 32	SMM	162574	0.60490814	0.480274934
CRDS-MC-0503-001	SMM-023	Cycle 4	SMM	220717	0.5567597	0.423994067
CRDS-MC-0569-001	SMM-023	Cycle 8	SMM	160306	0.632277678	0.530246707
SMM-PBMC-483	SMM-024	Baseline	SMM	171696	0.633540543	0.541534555
CRDS-MC-0502-001	SMM-024	Cycle 1	SMM	88538	0.361599253	0.270271692
CRDS-MC-0779-001	SMM-024	Cycle 20	SMM	154896	0.322434162	0.163417582
CRDS-MC-0528-001	SMM-024	Cycle 4	SMM	132445	0.312259725	0.175115742
CRDS-MC-0591-001	SMM-024	Cycle 8	SMM	134865	0.315079516	0.19344054
CRDS-MC-0477-001	SMM-025	Baseline	SMM	241181	0.562457704	0.334762477
CRDS-MC-0486-001	SMM-025	Cycle 1	SMM	154179	0.461680593	0.24559683
CRDS-MC-0794-001	SMM-025	Cycle 20	SMM	201120	0.589628331	0.404583364
CRDS-MC-0947-001	SMM-025	Cycle 32	SMM	147551	0.652582132	0.485819666
CRDS-MC-0537-001	SMM-025	Cycle 4	SMM	238828	0.663387869	0.492445898
CRDS-MC-0599-001	SMM-025	Cycle 8	SMM	246632	0.689632272	0.505793995
CRDS-MC-0490-001	SMM-026	Baseline	SMM	394122	0.336682747	0.072965505
CRDS-MC-0508-001	SMM-026	Cycle 1	SMM	312058	0.348331731	0.079307578
CRDS-MC-0804-001	SMM-026	Cycle 20	SMM	333508	0.331455368	0.058277063
CRDS-MC-0950-001	SMM-026	Cycle 32	SMM	167461	0.34544688	0.097240695
CRDS-MC-0549-001	SMM-026	Cycle 4	SMM	336936	0.315520216	0.05773878
CRDS-MC-0613-001	SMM-026	Cycle 8	SMM	223583	0.274279397	0.066921375
CRDS-MC-0523-001	SMM-027	Baseline	SMM	312098	0.31444211	0.091822282
CRDS-MC-0541-001	SMM-027	Cycle 1	SMM	129822	0.250992263	0.084512991
CRDS-MC-0858-001	SMM-027	Cycle 20	SMM	193928	0.238725457	0.055023056
CRDS-MC-0607-001	SMM-027	Cycle 4	SMM	118514	0.259227737	0.103902026

S2 Table. Number of unique productive sequences, Gini coefficient, and downsampled Gini coefficient computed per sample. Only healthy donors ≥ 40 years of age were used in the final analysis.

Sample	Patient	Time point	Diagnosis	Productive rearangements	Gini	Gini downsampled
CRDS-MC-0675-001	SMM-027	Cycle 8	SMM	125397	0.33922623	0.169524162
CRDS-MC-0526-001	SMM-028	Baseline	SMM	377761	0.375808626	0.101956021
CRDS-MC-0543-001	SMM-028	Cycle 1	SMM	351727	0.372082606	0.088152945
CRDS-MC-0838-001	SMM-028	Cycle 20	SMM	255023	0.301824586	0.066995866
CRDS-MC-0589-001	SMM-028	Cycle 4	SMM	80644	0.326285812	0.138909721
CRDS-MC-0655-001	SMM-028	Cycle 8	SMM	187841	0.339057708	0.107725634
CRDS-MC-0536-001	SMM-029	Baseline	SMM	327313	0.160389303	0.030083277
CRDS-MC-0555-001	SMM-029	Cycle 1	SMM	227969	0.174360531	0.034983881
CRDS-MC-0846-001	SMM-029	Cycle 20	SMM	147602	0.17609428	0.052618426
CRDS-MC-0601-001	SMM-029	Cycle 4	SMM	186264	0.138568854	0.027257154
CRDS-MC-0663-001	SMM-029	Cycle 8	SMM	88579	0.136795209	0.036607367
CRDS-MC-0531-001	SMM-030	Baseline	SMM	389876	0.164774606	0.032982049
CRDS-MC-0544-001	SMM-030	Cycle 1	SMM	220522	0.190576619	0.051488997
CRDS-MC-0842-001	SMM-030	Cycle 20	SMM	132339	0.239186794	0.129964352
CRDS-MC-0594-001	SMM-030	Cycle 4	SMM	220690	0.333574516	0.203793065
CRDS-MC-0662-001	SMM-030	Cycle 8	SMM	215150	0.277789329	0.139395927
CRDS-MC-0548-001	SMM-031	Baseline	SMM	397654	0.25743242	0.053453916
CRDS-MC-0564-001	SMM-031	Cycle 1	SMM	205033	0.201304707	0.046039885
CRDS-MC-0878-001	SMM-031	Cycle 20	SMM	181984	0.330664044	0.12684039
CRDS-MC-0612-001	SMM-031	Cycle 4	SMM	275294	0.199731545	0.033970814
CRDS-MC-0680-001	SMM-031	Cycle 8	SMM	247608	0.26427612	0.065658238
CRDS-MC-0570-001	SMM-032	Baseline	SMM	267313	0.459988566	0.26328491
CRDS-MC-0587-001	SMM-032	Cycle 1	SMM	139112	0.366718741	0.199416546
CRDS-MC-0637-001	SMM-032	Cycle 4	SMM	125593	0.534745433	0.394972781
CRDS-MC-0695-001	SMM-032	Cycle 8	SMM	152190	0.55222104	0.389946579
CRDS-MC-0575-001	SMM-033	Baseline	SMM	293710	0.39164937	0.161364683
CRDS-MC-0593-001	SMM-033	Cycle 1	SMM	157655	0.260594284	0.090017578
CRDS-MC-0881-001	SMM-033	Cycle 20	SMM	127647	0.391713755	0.20322217
CRDS-MC-0645-001	SMM-033	Cycle 4	SMM	213754	0.367789026	0.138405985
CRDS-MC-0700-001	SMM-033	Cycle 8	SMM	181250	0.323139793	0.106569966
CRDS-MC-0615-001	SMM-034	Baseline	SMM	141061	0.157509584	0.039755688
CRDS-MC-0636-003	SMM-034	Cycle 1	SMM	178809	0.23004813	0.055425007
CRDS-MC-0888-001	SMM-034	Cycle 20	SMM	204469	0.245918455	0.059608571
CRDS-MC-0683-001	SMM-034	Cycle 4	SMM	139168	0.187929694	0.043718645
CRDS-MC-0739-001	SMM-034	Cycle 8	SMM	147644	0.177725958	0.039863307
CRDS-MC-0622-001	SMM-035	Baseline	SMM	233178	0.240009947	0.06044583
CRDS-MC-0643-001	SMM-035	Cycle 1	SMM	143110	0.192342769	0.049376005
CRDS-MC-0892-001	SMM-035	Cycle 20	SMM	142592	0.222624605	0.053104036
CRDS-MC-0687-001	SMM-035	Cycle 4	SMM	125422	0.168969532	0.044861124
CRDS-MC-0761-001	SMM-035	Cycle 8	SMM	190894	0.149287421	0.025132056
CRDS-MC-0628-001	SMM-036	Baseline	SMM	270987	0.293320148	0.148791931
CRDS-MC-0648-001	SMM-036	Cycle 1	SMM	211239	0.267793391	0.094439609
CRDS-MC-0900-001	SMM-036	Cycle 20	SMM	190762	0.332900422	0.158966567
CRDS-MC-0689-001	SMM-036	Cycle 4	SMM	171601	0.275587788	0.117260711
CRDS-MC-0763-001	SMM-036	Cycle 8	SMM	268349	0.295609485	0.126215485
CRDS-MC-0627-001	SMM-037	Baseline	SMM	233646	0.681392577	0.41064439
CRDS-MC-0647-001	SMM-037	Cycle 1	SMM	198696	0.59164983	0.301560197
CRDS-MC-0896-001	SMM-037	Cycle 20	SMM	174491	0.432599426	0.152080233
CRDS-MC-0690-001	SMM-037	Cycle 4	SMM	181179	0.492354044	0.195164587
CRDS-MC-0757-001	SMM-037	Cycle 8	SMM	113074	0.317938924	0.108620145
CRDS-MC-0653-001	SMM-038	Baseline	SMM	242830	0.265349718	0.086193489
CRDS-MC-0673-001	SMM-038	Cycle 1	SMM	153114	0.266484333	0.137175153
CRDS-MC-0919-001	SMM-038	Cycle 20	SMM	180285	0.266606543	0.106057021
CRDS-MC-0713-001	SMM-038	Cycle 4	SMM	96564	0.266347649	0.133839418
CRDS-MC-0778-001	SMM-038	Cycle 8	SMM	174632	0.228047385	0.08339518
CRDS-MC-0659-001	SMM-039	Baseline	SMM	220997	0.565386712	0.396646948

S2 Table. Number of unique productive sequences, Gini coefficient, and downsampled Gini coefficient computed per sample.
Only healthy donors ≥ 40 years of age were used in the final analysis.

Sample	Patient	Time point	Diagnosis	Productive rearangements	Gini	Gini downsampled
CRDS-MC-0679-001	SMM-040	Baseline	SMM	374160	0.429230123	0.230881586
CRDS-MC-0691-001	SMM-040	Cycle 1	SMM	189886	0.237926852	0.091017209
CRDS-MC-0750-001	SMM-040	Cycle 4	SMM	357815	0.261789836	0.099332811
CRDS-MC-0828-001	SMM-040	Cycle 8	SMM	105313	0.43598861	0.32290982
CRDS-MC-0694-001	SMM-041	Baseline	SMM	270567	0.232397106	0.084417936
CRDS-MC-0714-001	SMM-041	Cycle 1	SMM	165845	0.247258475	0.092574414
CRDS-MC-0766-001	SMM-041	Cycle 4	SMM	254586	0.180317496	0.037087664
CRDS-MC-0834-001	SMM-041	Cycle 8	SMM	189201	0.266625296	0.12194584
CRDS-MC-0702-001	SMM-042	Baseline	SMM	217120	0.302053023	0.136042094
CRDS-MC-0723-001	SMM-042	Cycle 1	SMM	176622	0.284448669	0.125837161
CRDS-MC-0773-001	SMM-042	Cycle 4	SMM	182965	0.317070261	0.186408007
CRDS-MC-0830-001	SMM-042	Cycle 8	SMM	232565	0.396271188	0.238568434
CRDS-MC-0697-001	SMM-043	Baseline	SMM	204637	0.253458782	0.102949702
CRDS-MC-0719-001	SMM-043	Cycle 1	SMM	131049	0.214174863	0.080942175
CRDS-MC-0770-001	SMM-043	Cycle 4	SMM	263314	0.160841783	0.050218447
CRDS-MC-0825-001	SMM-043	Cycle 8	SMM	163819	0.149363975	0.041650829
CRDS-MC-0718-001	SMM-044	Baseline	SMM	225896	0.419997565	0.1886761
CRDS-MC-0735-001	SMM-044	Cycle 1	SMM	128111	0.272056304	0.11028781
CRDS-MC-0780-001	SMM-044	Cycle 4	SMM	211687	0.284719294	0.098441449
CRDS-MC-0839-001	SMM-044	Cycle 8	SMM	256884	0.398821151	0.199432857
CRDS-MC-0759-001	SMM-045	Baseline	SMM	92834	0.248117689	0.097766862
CRDS-MC-0775-001	SMM-045	Cycle 1	SMM	91906	0.216798495	0.097642133
CRDS-MC-0820-001	SMM-045	Cycle 4	SMM	137370	0.304131283	0.137889805
CRDS-MC-0874-001	SMM-045	Cycle 8	SMM	176378	0.405610621	0.193213848
CRDS-MC-0776-001	SMM-046	Baseline	SMM	227937	0.436735441	0.299660209
CRDS-MC-0784-001	SMM-046	Cycle 1	SMM	160184	0.367439084	0.22899883
CRDS-MC-0833-001	SMM-046	Cycle 4	SMM	130431	0.355506181	0.226464707
CRDS-MC-0885-001	SMM-046	Cycle 8	SMM	230669	0.633696054	0.48948824
CRDS-MC-0810-001	SMM-047	Baseline	SMM	328173	0.370435755	0.214554258
CRDS-MC-0835-001	SMM-047	Cycle 1	SMM	261531	0.280597902	0.113654541
CRDS-MC-0866-001	SMM-047	Cycle 4	SMM	196510	0.371936295	0.22651221
CRDS-MC-0886-001	SMM-047	Cycle 8	SMM	188823	0.296482549	0.114073912
CRDS-MC-0829-001	SMM-048	Baseline	SMM	305922	0.361591652	0.222853086
CRDS-MC-0844-001	SMM-048	Cycle 1	SMM	91159	0.15381056	0.082845803
CRDS-MC-0909-001	SMM-048	Cycle 4	SMM	127288	0.244139796	0.128453514
CRDS-MC-0831-001	SMM-049	Baseline	SMM	216187	0.218364627	0.084457849
CRDS-MC-0847-001	SMM-049	Cycle 1	SMM	189309	0.171836603	0.043130058
CRDS-MC-0914-001	SMM-049	Cycle 4	SMM	135951	0.311041345	0.150294583
CRDS-MC-0855-001	SMM-050	Baseline	SMM	215577	0.267538792	0.126384306
CRDS-MC-0868-001	SMM-050	Cycle 1	SMM	167584	0.386113563	0.212011073
CRDS-MC-0943-001	SMM-050	Cycle 4	SMM	185064	0.38543558	0.203120806
CRDS-MC-0860-001	SMM-051	Baseline	SMM	167849	0.295949344	0.170946421
CRDS-MC-0867-001	SMM-051	Cycle 1	SMM	189748	0.295034903	0.131354535
CRDS-MC-0942-001	SMM-051	Cycle 4	SMM	134758	0.494665388	0.362532362
CRDS-MC-0884-001	SMM-052	Baseline	SMM	155764	0.136247157	0.035163572
CRDS-MC-0889-001	SMM-052	Cycle 1	SMM	174749	0.263715633	0.089363752
CRDS-MC-0887-001	SMM-053	Baseline	SMM	190729	0.321913333	0.125919284
CRDS-MC-0902-001	SMM-053	Cycle 1	SMM	193458	0.31935162	0.11190666
CRDS-MC-0895-001	SMM-054	Baseline	SMM	208970	0.284474911	0.1197364
CRDS-MC-0913-001	SMM-054	Cycle 1	SMM	173973	0.319453935	0.129509093
CRDS-MC-0911-001	SMM-055	Baseline	SMM	217844	0.520516758	0.410024277
CRDS-MC-0933-001	SMM-055	Cycle 1	SMM	143266	0.459843094	0.307785922
