# Supplementary material for: Machine learning reveals distinct T-cell receptor clusters in plasma cell dyscrasias compared to healthy controls
Source: PLoS One. 2025 Oct 27;20(10):e0334053. doi: 10.1371/journal.pone.0334053 (PMC12558469; doi:10.1371/journal.pone.0334053)
Supplement: S3 Table — Mean physical property for each cluster are shown. Sample count are provided for healthy and plasma cell dyscrasia (PCD) patients. Wilcoxon Rank Sum test P value and false discovery rate (FDR) are reported for each cluster along with top performing machine learning model variable importance rank (1 is the most important and 507 is the least). Length, number of amino acids; gravy, grand average of hydrophobicity index; bulk, average bulkiness of amino acids; aliphatic, aliphatic index; polarity, average polarity of amino acids; charge, net charge; basic, fraction of informative positions that are Arg, His or Lys; acidic, fraction of informative positions that are Asp or Glu; aromatic, fraction of informative positions that are His, Phe, Trp or Tyr. (PDF) [file pone.0334053.s004.pdf]

S4 Table. Performance metrics for each classifier across five repeated stratified 80/20 train/test splits. For each machine learning model, performance was evaluated using five independent train/test iterations. AUROC area under the receiver operating characteristic curve; rf, Random Forest; svmRadial, Support Vector Machine with Radial Basis Function Kernel; glmnet, Elastic Net Regularization; nnet, Neural Network; gbm, Gradient Boosting Machine; knn, k-Nearest Neighbors.

Repeat	Model	Accuracy	Kappa	Sensitivity	Specificity	AUROC
1	rf	0.957	0.913	0.913	1.000	1.000
2	rf	0.978	0.957	1.000	0.957	1.000
3	rf	0.978	0.957	0.957	1.000	1.000
4	rf	0.957	0.913	0.957	0.957	0.998
5	rf	0.957	0.913	0.957	0.957	0.996
1	svmRadial	1.000	1.000	1.000	1.000	1.000
2	svmRadial	1.000	1.000	1.000	1.000	1.000
3	svmRadial	1.000	1.000	1.000	1.000	1.000
4	svmRadial	0.978	0.957	0.957	1.000	1.000
5	svmRadial	0.957	0.913	0.957	0.957	0.996
1	glmnet	0.978	0.957	0.957	1.000	1.000
2	glmnet	0.978	0.957	0.957	1.000	0.994
3	glmnet	1.000	1.000	1.000	1.000	1.000
4	glmnet	1.000	1.000	1.000	1.000	1.000
5	glmnet	1.000	1.000	1.000	1.000	1.000
1	nnet	0.848	0.696	1.000	0.696	0.870
2	nnet	0.978	0.957	0.957	1.000	0.975
3	nnet	0.978	0.957	1.000	0.957	0.976
4	nnet	0.957	0.913	0.913	1.000	0.978
5	nnet	0.957	0.913	0.957	0.957	0.977
1	gbm	0.891	0.783	0.826	0.957	0.911
2	gbm	0.848	0.696	0.913	0.783	0.896
3	gbm	0.870	0.739	0.826	0.913	0.943
4	gbm	0.652	0.304	0.565	0.739	0.760
5	gbm	0.674	0.348	0.783	0.565	0.783
1	knn	0.783	0.565	0.565	1.000	0.957
2	knn	0.739	0.478	0.478	1.000	0.883
3	knn	0.717	0.435	0.435	1.000	0.839
4	knn	0.696	0.391	0.391	1.000	0.836
5	knn	0.674	0.348	0.348	1.000	0.913
