# Supplementary material for: Machine learning reveals distinct T-cell receptor clusters in plasma cell dyscrasias compared to healthy controls
Source: PLoS One. 2025 Oct 27;20(10):e0334053. doi: 10.1371/journal.pone.0334053 (PMC12558469; doi:10.1371/journal.pone.0334053)
Supplement: Attachment — Submitted filename: Review for Manuscript Number PONE-D-25-19122.docx [file pone.0334053.s006.docx]

The manuscript presents an extensive T-cell–receptor (TCR) repertoire analysis across the plasma-cell-dyscrasia continuum and introduces a convergent-clustering with machine-learning (ML) pipeline that reportedly distinguishes patients from healthy donors. The topic is important and, in principle, within PLOS ONE’s scope. However, several methodological and interpretative weaknesses currently prevent me from recommending acceptance. Addressing the issues below should be feasible without re-designing the study, but will require substantial re-analysis and clarification.

1. Differential-abundance screening uses a two-sided Fisher test with α = 0.001 but no FDR control. With 1.24 M tests, ~1 240 false-positive clusters are expected by chance. Re-run the analysis with FDR correction and report the number of clusters that remain significant. Retrain the ML models with this revised feature set.
2. AUROC = 0.84 is based on a single 70/30 random split drawn from the same sequencing batches. No confidence interval (CI) is provided; external generalisability is unknown.

Please: (a) Implement repeated stratified hold-outs (e.g., 5 × 20 %) or provide an independent validation cohort. (b) Report AUROC, accuracy, sensitivity, specificity, and Kappa with 95 % CIs. (c) Compare the best model to the others with a statistical test (e.g., DeLong).

3. Provide an enrichment analysis comparing antigen types in disease-enriched vs healthy-enriched clusters. If no significant bias emerges, tone down the claim that the 111/507 clusters containing database-matched CDR3βs “likely reflect differential antigen recognition.”.
4. Except for the R packages and their versions, please also clarify the executable workflow or script directory with hyperparameters.

In summary, this study has clear potential. Addressing the above issues and documenting the full reproducibility pipeline should bring the manuscript up to the “methodologically sound” standard required for publication.
