# Supplementary material for: Machine learning reveals distinct T-cell receptor clusters in plasma cell dyscrasias compared to healthy controls
Source: PLoS One. 2025 Oct 27;20(10):e0334053. doi: 10.1371/journal.pone.0334053 (PMC12558469; doi:10.1371/journal.pone.0334053)
Supplement: Attachment — Submitted filename: Reviewer_comments_auresp_2.docx [file pone.0334053.s009.docx]

**RESPONSE TO REVIEWER COMMENTS**

Reviewer #1: This manuscript presents an analysis of the peripheral blood T-cell receptor (TCR) β-chain repertoire in patients with monoclonal gammopathy of undetermined significance (MGUS), multiple myeloma (MM), and healthy controls (HC), using high-throughput sequencing. The authors identify distinct TCR repertoire features across groups, including differences in clonal expansion, public/shared clones, and CDR3 amino acid usage.

The study offers a novel perspective on the immune microenvironment in plasma cell dyscrasias and highlights the potential utility of TCR repertoire profiling in disease stratification. However, there are several significant concerns related to cohort characterization, data interpretation, and methodological clarity that must be addressed before the manuscript can be considered for publication.

1. The manuscript lacks key clinical details about the MGUS and MM cohorts. It is not specified whether patients were treatment-naïve, or if any had received prior immunosuppressive therapy. Since TCR diversity is highly sensitive to immune status, such information is crucial for data interpretation.

We thank the reviewer for raising this important point. To clarify, the MGUS and MM cohorts include patients who were treatment naïve at the time of sample collection, and we have revised the methods section accordingly.

**Revised Methods:**

*TCR sequencing was performed on the peripheral blood mononuclear cells (PBMCs) of 80 patients with untreated MGUS, 55 with SMM, and 31 with newly diagnosed MM before treatment (NDMM, S1 Table).*

2. Additionally, information on infection or inflammatory status should be included, as these factors can influence TCR repertoire features independently of disease state.

We thank the reviewer for this suggestion. While infection and inflammatory status are key variables that can influence the TCR repertoire, this information was not systematically collected for our cohort per IRB protocol, and we are unable to include it in the analysis. We have now acknowledged this limitation in the discussion section of the manuscript.

**Revised Discussion:**

*The biological significance of these differentially abundant clusters remains uncertain, but several possibilities may explain their enrichment in plasma cell dyscrasias. First, these clusters could represent tumor-reactive T cells that have undergone clonal expansion in response to malignant or pre-malignant plasma cell antigens. Second, they may reflect bystander T-cell expansions driven by chronic immune activation, microbial exposure, or tissue remodeling. Third, differences in the TCRB repertoire between patients and controls could be influenced by non-disease factors such as subclinical infections or vaccination history. We acknowledge that information regarding recent infections, inflammatory status, and prior immunization was not systematically collected for all study patients.*

3. The study includes only 10 healthy controls, 8 MGUS, and 12 MM patients. The small cohort size limits the robustness and generalizability of the findings, especially for analyses of public clone distribution and amino acid bias.

We apologize if this was not sufficiently clear in the manuscript and would like to clarify the cohort numbers. The analysis was performed on 80 patients with untreated MGUS, 55 with SMM, 31 with newly diagnosed MM, and 166 healthy controls. To our knowledge, this is the largest T-cell repertoire study in patients with plasma cell dyscrasias to date.

4. The authors are encouraged to incorporate effect size metrics, confidence intervals, or bootstrapping to enhance statistical reliability.

We thank the reviewer for this constructive suggestion to enhance the statistical reliability of our findings. We agree that effect size metrics and confidence intervals are essential for robust interpretation and have incorporated these statistical measures throughout the manuscript:

- Confidence intervals: We have included 95% confidence intervals in our machine learning performance evaluation, specifically for the AUROC, accuracy, sensitivity, and specificity metrics, as displayed in Figure 3C.
- Effect size: For comparisons of amino acid characteristics and the Gini coefficient, effect size is determined by Wilcoxon rank-sum test p-values as shown in Figures 2 and 4.
- Bootstrapping: We employed a repeated stratified hold-out procedure (5 iterations of 80/20 splits) for internal validation. This bootstrapping-like approach allowed us to assess the stability and performance of our models on different subsets of the data, with the results summarized as means and 95% confidence intervals.

5. The criteria used to define “clonal expansion,” “shared clones,” and “public clones” are not clearly described. For example, what frequency threshold is used to identify expanded clones? Please provide a consistent and detailed definition of these terms in the Methods section and ensure they are applied uniformly throughout the analysis.

We thank the reviewer for highlighting the need to more clearly define these key terms. The terms in question are used in our Background and Discussion sections to provide context from the existing literature. To provide further clarity, below is how we interpret these concepts:

- Clonal expansion: In the cited literature, this often refers to an increase in the frequency of specific T-cell clones, sometimes identified by an arbitrary frequency threshold. In contrast, our study avoids such thresholds by measuring the repertoire clonality as a continuous variable using the Gini coefficient.
- Shared and public clones: The literature often uses “shared clones” to refer to identical TCR sequences found in different individuals, while “public clones” are well-known examples of these shared sequences, such as the ones listed in the databases we queried. Our study moves beyond simple sequence identity to identify functionally shared immune responses using ClusTCR to cluster structurally similar TCRs.

7. The study lacks any form of validation—either technical replicates or an independent validation cohort. The reproducibility of the sequencing data should be addressed, potentially through read saturation plots or duplicate library controls.

We thank the reviewer for raising the critical point of study validation and reproducibility. We acknowledge that validating our findings in a separate, independent patient cohort would be the gold standard. While this was not feasible for the present study, we did incorporate a rigorous validation step for our primary machine learning findings. Specifically, we used a stratified 70/30 split of our data. The 30% held-out test set was completely isolated from all feature selection and model training steps. This held-out set functions as an independent validation set for the machine learning model, and the performance on this set (AUROC 0.859) demonstrates the generalizability of the identified TCRB signature.

8. Figures are generally informative, but legends could be improved. For example, Figures 2 and 4 require clearer color legends and more descriptive axis labeling.

We thank the reviewer for their thoughtful feedback on our figures and for the suggestion to improve clarity. We have used distinct colors for the box plots in Figures 2A-D and 4B to provide clear visual separation between the different experimental groups and timepoints. We find this is particularly helpful for the reader in quickly distinguishing the adjacent plots within the dense, multi-panel figures, thereby improving overall readability. All other figures have legends describing the meaning of the color.

9. Figure 5 would benefit from including statistical annotations (e.g., asterisks for p-values) to indicate the significance of amino acid usage differences.

We thank the reviewer for the suggestion to enhance the presentation of our statistical findings. We believe this comment refers to Figure 4B, which details the differences in physicochemical properties, as this is where the significance of amino acid characteristics is presented. In designing the figure, we made the choice to present the exact p-values rather than using asterisk annotations on the plot. Our rationale is that providing the precise p-value offers a higher degree of statistical detail and transparency than the categorical thresholds represented by asterisks

10. The manuscript briefly mentions clonal expansion and public clones but does not explore underlying mechanisms or implications. Are these changes driven by specific antigens? Is there evidence for immune exhaustion or senescence in MGUS/MM?

We thank the reviewer for these excellent questions. In our analysis, we did not observe clonal expansion of public clones, nor a disproportionate abundance of public clones when comparing healthy individuals to those with plasma cell dyscrasias. In the discussion, we note that “future studies should focus on identifying the antigenic targets of the TCRs that comprise the differentially abundant TCRB clusters identified in this study. This could provide valuable insights into the mechanisms underlying the immune response in plasma cell dyscrasias and potentially reveal novel therapeutic targets. Additionally, exploring the phenotypic states and functional properties of these distinct T-cell populations could further elucidate their role in disease pathogenesis and response to therapy.”

11. A more detailed discussion of how repertoire features may reflect tumor-immune interactions would be valuable.

We thank the reviewer for these insightful comments, which address key areas of interest in the field. Our study was focused on the TCR repertoire and was not designed to directly assess antigen specificity or T-cell functional states. However, our findings provide a foundation for these critical future investigations. In the discussion, we acknowledge that “The biological significance of these differentially abundant clusters remains uncertain, but several possibilities may explain their enrichment in plasma cell dyscrasias. First, these clusters could represent tumor-reactive T cells that have undergone clonal expansion in response to malignant or pre-malignant plasma cell antigens. Second, they may reflect bystander T-cell expansions driven by chronic immune activation, microbial exposure, or tissue remodeling. Third, differences in the TCRB repertoire between patients and controls could be influenced by non-disease factors such as subclinical infections or vaccination history.”

12. The manuscript is generally well written but could benefit from some minor language polishing. Some expressions (e.g., “suggests a trend”) should be supported by statistical evidence or removed.

We thank the reviewer for their positive feedback and for the helpful suggestion to ensure our language is precise. Our use of the term “trend” is intended to describe a consistent pattern in the data that we believe may be biologically relevant, even when it does not meet our pre-defined threshold for statistical significance. To ensure full transparency, in every instance where we mention a trend (e.g., in our discussion of Figures 2 and S4), we have also reported the outcome of the corresponding statistical test. This allows the reader to independently assess both the visual pattern and its level of statistical support.

13. Gene and protein names should follow standard formatting conventions throughout.

We have carefully reviewed the entire manuscript to ensure that all gene and protein names adhere to standard formatting conventions.

Reviewer #2: I appreciate the authors’ comprehensive responses to the first-round comments. The revised version is improved in clarity, methodological rigor, and scientific presentation.

The study presents a valuable analysis of the TCRB repertoire in plasma cell dyscrasias, with integration of immune repertoire sequencing, machine learning-based classification, and antigen annotation. The distinction between global TCR diversity and cluster-level features appropriately highlights the complexity of T-cell biology in this disease context.

While I support the publication of this work, I have a few minor comments and suggestions as outlined below.

1. The MRD results are mentioned briefly. Does MRD status correlate with any TCR feature?

We thank the reviewer for this question. We investigated whether achieving sustained MRD negativity in high-risk SMM patients was associated with overall TCRB diversity. As shown in Figure 2E and stated in the Results and Discussion sections, our analysis did not reveal a statistically significant difference in TCRB diversity between patients who achieved sustained MRD negativity and those who did not.

2. Line 259-261: The overlap analysis is interesting but lacks interpretation. Is this overlap more or less than expected by chance?

We thank the reviewer for raising this important point. In our initial analysis, the Venn diagram in Figure 3A included all available samples, which meant that some patients contributed more than others if they had multiple time points. To avoid potential bias from unequal sampling and to ensure that each patient contributed equally, we revised the analysis to include only baseline samples. This approach provides a clearer and more consistent representation of exclusivity across disease groups.

To assess whether the observed group-specific exclusivity differed from what would be expected by chance, we performed a permutation test. Specifically, we preserved the number of baseline samples in each disease category (Healthy, MGUS, SMM, MM) and randomly permuted group labels across samples 1,000 times. For each permutation, we recalculated the proportion of clusters exclusive to each group. We then compared the observed exclusivity to the null distribution, computing mean and two-sided permutation p-values.

The results below indicate that the observed exclusivity percentages were generally consistent with chance expectations. MGUS (10.7% observed vs. 10.0% expected, p = 0.42) and MM (3.4% vs. 3.6%, p = 0.77) fell within the permutation-derived confidence intervals, while SMM (8.6% vs. 6.6%, p = 0.016) and healthy (21.3% vs. 24.4%, p = 0.04) showed modest but statistically significant enrichment. Overall, these results suggest that the exclusivity of TCR clusters across diagnostic categories is largely consistent with random expectation, with SMM and healthy representing potential exceptions. The methods and results sections have been updated accordingly.

**Revised Methods:**
*Group-specific TCRB cluster exclusivity was evaluated by permutation testing, in which baseline sample labels were shuffled 1,000 times while preserving group sizes, and observed exclusivity was compared against the resulting null distribution to derive empirical p-values.*

**Revised Results:**

*Overlap analyses between baseline sample clusters demonstrated that 21.3% are exclusive to healthy, 10.7% to MGUS, 8.6% to SMM, and 3.4% to MM (Figure 3A). A permutation test confirmed that the observed exclusivity percentages were consistent with chance expectations for MGUS (10.7% observed vs. 10.0% expected, p = 0.42) and MM (3.4% vs. 3.6%, p = 0.77) while SMM (8.6% vs. 6.6%, p = 0.016) and healthy (21.3% vs. 24.4%, p = 0.04) showed evidence of enrichment beyond random expectations.*

3. Line 266: How were variable importance scores interpreted across different models? Did top clusters in different models overlap significantly?

We thank the reviewer for this excellent suggestion. We compared variable importance across the four top-performing models (Random Forest, Support Vector Machines with RBF kernel, Elastic Net, and Neural Network) using pairwise Spearman correlations of the variable-importance rankings. This analysis showed that Random Forest and Support Vector Machines demonstrated the greatest similarity (r ≈ 0.47), while Random Forest also exhibited modest agreement with Elastic Net (r ≈ 0.23) and Neural Network (r ≈ 0.15). In contrast, Elastic Net showed little to no alignment with either Support Vector Machines (r ≈ –0.18) or Neural Network (r ≈ 0.02). These results indicate that some clusters are consistently ranked highly across models, particularly between Random Forest and Support Vector Machines, while others are more model-specific. The concordance between non-linear models such as Random Forest and Support Vector Machines strengthens confidence in the robustness of the commonly identified clusters, whereas the weaker alignment with the linear Elastic Net model suggests that the underlying biological signal may be complex and non-linear. We have updated Table S3 to provide the full variable-importance rankings for each of the four top-performing models.

**Spearman correlation of variable importance rankings**

4. Please emphasize more clearly that differentially abundant clusters were identified despite no difference in total diversity, which strengthens the idea that qualitative rather than quantitative TCR features matter.

We agree this is a central conclusion of our study and have revised the Discussion section to emphasize this point more clearly.

**Revised Discussion:**

“Although overall TCRB diversity was not associated with clinical outcomes, our machine learning model trained on differentially abundant clusters was able to distinguish healthy individuals from patients with plasma cell dyscrasias, underscoring that qualitative rather than quantitative TCR features are driving these differences.”

5. The limitation that many sequences did not map to known antigens may reflect gaps in current databases, not necessarily lack of disease relevance.

We agree completely with the reviewer's insightful point. We have revised our Discussion section to frame this finding not merely as a limitation, but as a reflection of the current state of public antigen databases.

**Revised Discussion:**

“Notably, most of the differentially abundant clusters we identified did not map to known antigens. This likely reflects the significant gaps in current TCR-antigen reference databases rather than a true lack of disease relevance. These unannotated, disease-associated clusters may in fact represent T-cell responses to novel tumor neoantigens or other undiscovered disease-specific epitopes, making them high-priority candidates for future functional investigation.”

Reviewer #3:

1. Validation of Clustering Results

a. The current manuscript does not report how stable or robust the clustering results are. The authors didn’t clarify whether they ran the clustering multiple times to assess consistency.

We thank the reviewer for this important comment and for the opportunity to clarify our methodology. In our study, we used ClustTCR to group TCRB CDR3 sequences based on similarity in amino acid sequences. Importantly, the ClustTCR algorithm is deterministic, meaning that given the same input data and parameters, it will produce the same set of clusters every time. Because there is no random element in the clustering process, the results are stable and reproducible by design. Running the algorithm multiple times would only produce identical outputs.

b. No silhouette score, Davies–Bouldin index, or other clustering quality metric is reported to justify the choice of the number of clusters.

We thank the reviewer for raising this point. The ClusTCR algorithm we used is fundamentally different from partitioning methods like k-means, where a user must pre-specify the number of clusters. ClusTCR is a graph-based clustering method where the number of clusters is an output of the analysis, emerging naturally from the data’s structure based on a similarity threshold. Therefore, quality metrics like the Silhouette score, which are used to justify the choice of a pre-set number of clusters, are not applicable to our method. We used the default parameters recommended in the original ClusTCR publication.

2. Sample Size and Overfitting

a. The ML models (especially clustering) are susceptible to overfitting, particularly with limited samples. It’s unclear whether the authors used methods to address this (e.g., bootstrapping, downsampling, or dimensionality selection tuning).

We thank the reviewer for raising this critical point. We agree that preventing overfitting is paramount and would like to clarify the multiple steps we took to ensure the robustness of our findings, as detailed in the Methods section.

- To ensure an unbiased assessment of final model generalizability, we partitioned our data into a 70% training set for model development and a 30% held-out test set for a single, final evaluation.
- To prevent information leakage during feature selection, we identified differentially abundant TCRB clusters using only the 70% training data.
- To obtain a stable estimate of performance during model tuning, we employed a repeated hold-out procedure, similar to bootstrapping, which exclusively used the training set, thereby protecting against results being an artifact of any single data split.

We are confident that this comprehensive approach ensures that our reported results are robust and minimize overfitting.

3. Biological Interpretation of Clusters

a. Some clusters are interpreted as being specific to disease state in their current manuscript, but this is not rigorously tested. A formal statistical test comparing cluster membership distributions across disease vs. healthy controls is needed.

We completely agree that a formal statistical test is required to rigorously identify clusters that are differentially abundant between the disease and healthy cohorts. This is precisely the approach we took for our analysis. As detailed in the Methods section (under “Statistical analysis”), we performed a two-sided Fisher’s Exact Test for each cluster to compare its prevalence in the plasma cell dyscrasia cohort with that in the healthy control cohort. The results of this analysis are described in the Results section, where we state that we identified 507 differentially abundant clusters at a significance threshold of p < 0.001. These 507 clusters were the features used for all subsequent machine learning models. The value of the P values and false discovery rates are reported in Table S3.

b. Additionally, reporting effect sizes and confidence intervals would strengthen the inferences.

We agree that effect size metrics and confidence intervals are essential for robust interpretation and have incorporated these statistical measures throughout the manuscript:

- Confidence Intervals: We have included 95% confidence intervals in our machine learning performance evaluation, specifically for the AUROC, accuracy, sensitivity, and specificity metrics, as displayed in Figure 3C.
- Effect Size: For comparisons of amino acid characteristics and the Gini coefficient, effect size is determined by Wilcoxon rank-sum test p-values as shown in Figures 2 and 4.

4. Lack of Integration with Clinical Data

a. Their study would benefit from correlating clusters or clonality features with available clinical parameters (e.g., disease stage, treatment status), if accessible.

We agree that correlating clonality features with clinical parameters is a primary goal of this work, and we would like to clarify where these analyses are presented throughout the manuscript. We performed several correlations between clonality (measured by the Gini coefficient) and key clinical parameters:

- Disease Stage: Figure 2A directly compares clonality across healthy controls, MGUS, SMM, and newly diagnosed MM patients.
- Treatment Status: Figure 2D tracks changes in clonality at multiple timepoints throughout treatment for MM patients.
- Clinical Outcome: Figure 2E correlates clonality with the achievement of sustained Minimal Residual Disease (MRD) negativity.
- Age: Supplementary Figure S2 shows the correlation between clonality and patient age

5. Multiple Testing Correction

a. No indication is given that corrections for multiple hypothesis testing were applied, despite numerous comparisons. This should be addressed in their next round.

We thank the reviewer for raising this critical point regarding multiple hypothesis testing. We agree completely and would like to clarify our approach. As detailed in the Methods section (under “Statistical analysis”), we perform a formal false discovery rate (FDR) correction on the p-values from all ~1.2 million cluster comparisons. However, due to the extremely large number of tests, no single cluster remained significant at a strict FDR of < 0.05. We have reported these FDR-corrected q-values for all clusters in Table S3.

Given that a strict FDR correction can be overly conservative in such high-dimensional settings, we then employed a common and pragmatic approach for feature selection. We used a stringent nominal p-value threshold of < 0.001 to identify a candidate set of 507 differentially abundant clusters to build our predictive models.

The validity of this feature set was ultimately demonstrated by the high performance of our model on the completely unseen held-out test set. This downstream validation confirms that our feature selection method successfully identified a robust and generalizable biological signature, even if individual features did not survive the most stringent statistical correction.
