# Supplementary material for: Machine learning reveals distinct T-cell receptor clusters in plasma cell dyscrasias compared to healthy controls
Source: PLoS One. 2025 Oct 27;20(10):e0334053. doi: 10.1371/journal.pone.0334053 (PMC12558469; doi:10.1371/journal.pone.0334053)
Supplement: Attachment — Submitted filename: Reviewer comments.docx [file pone.0334053.s008.docx]

**RESPONSES TO REVIEWER COMMENTS**

**Reviewer #1:** The study provides a detailed and large-scale analysis of TCR repertoire diversity in plasma cell dyscrasias, including MGUS, SMM, and MM, compared to healthy controls. The use of high-throughput sequencing and machine learning adds robustness to the findings. However, the study can be improved with the following aspects:

1. The abundant different TCR clusters between healthy and patients comes only from machine learning (ML)-identified clusters, which are not functionally validated. The title to reflect the ML findings more accurately.

We thank the reviewer for this insightful comment. We agree that the identification of differentially abundant TCR clusters between healthy individuals and patients with plasma cell dyscrasias in our study was based on machine learning models trained on high-throughput sequencing data, and that these clusters have not yet been functionally validated.

In response, we have revised the manuscript title to more accurately reflect the nature of our findings. The updated title is: **“Machine learning reveals distinct T-cell receptor clusters in plasma cell dyscrasias compared to healthy controls”.**

We believe this revised title better conveys that our conclusions are based on computational analysis rather than functional assays, and appreciate the reviewer’s suggestion to clarify this point.

2. Line 39 and lines 122-123: The rationale for focusing on TCRß is not explicitly justified, the authors can briefly explain why TCRß sequencing was prioritized (e.g., technical feasibility, prior literature).

We appreciate the reviewer’s suggestion to clarify the rationale for focusing on the TCRβ chain. In response, we have added a new paragraph to the Background section (now the second paragraph), which introduces the structure and function of the T-cell receptor and explains why the β chain, particularly the highly variable CDR3 region, is central to antigen recognition. This revision provides biological justification for our focus on TCRβ repertoire diversity in the context of plasma cell dyscrasias.

**Revised Background:**

*Each T cell expresses a unique T-cell receptor (TCR), most commonly composed of an α and a β chain, that determines antigen specificity through its interaction with peptide–MHC complexes. The complementarity-determining region 3 (CDR3) of the TCR, particularly within the β chain, is the most variable and critical for recognizing antigenic peptides. TCR diversity refers to the number and relative distribution of unique CDR3 sequences within a repertoire, capturing both the richness of distinct clonotypes and their evenness of representation. In MM and its precursor conditions, decreased repertoire diversity may indicate antigen-driven clonal expansions or T-cell senescence, whereas preserved diversity may reflect intact immune surveillance. Repertoire analysis can therefore provide important insights into immune competence, disease progression, and treatment response.*

3. Lines 115, 176, 230-231: The authors claim that the age of the healthy group was younger than the patient groups (MGUS/SMM/MM), please provide the median ages for all groups and discuss whether age-matching was attempted.

We appreciate the reviewer’s attention to this important methodological detail. In response, we have added the median ages of each group to the Results section to provide greater transparency. Specifically, the median age of healthy controls was 55 years, compared to 58 years for MGUS, 60 years for SMM, and 60 years for MM.

Additionally, we have clarified in the Methods section that *age-matching was not prospectively implemented, as the healthy control data were obtained from existing publicly available datasets. However, to mitigate the potential confounding effect of age, we excluded healthy donors under 40 years of age and adjusted for age in all multivariate analyses involving TCR repertoire diversity.*

4. In the main manuscript, the authors claimed that their TCRB sequencing data are available through ImmuneACCESS (e.g., lines 127-128, lines 160-161), but no accession numbers are provided. It will be better if they can provide a specific access accession code.

We thank the reviewer for pointing this out. We have updated the manuscript to include the specific DOI accession number associated with our dataset in the ImmuneACCESS data portal. The data can now be accessed at:

[https://clients.adaptivebiotech.com/pub/coffey-2025-s](https://urldefense.com/v3/__https:/clients.adaptivebiotech.com/pub/coffey-2025-s__;!!KVu0SnhVq1hAFvslES2Y!LncASm770FRyStjDnNCrAaHyoWCMWErA1PeidhTqj6L6TnSYmACFfPwKqdC3PGtQzHeLNfUHsbEFS8V42oP_ljj4K04zrA$) (DOI 10.21417/DGC2025S).

5. It will be better to standardize the format of the numbers in the main manuscript. e.g., line 213, change “1 240 017” to “1,240,017”.

We appreciate the reviewer’s suggestion to improve clarity and consistency in numerical formatting. We have revised the manuscript to use commas to separate thousands for large numbers throughout the text.

6. Line 228-229: The manuscript identified "differentially abundant TCRB clusters" only based on p-value. Is there any other parameters that are important for this identification in Supplementary Table 3? e.g, “Importance” or fold-change?

We thank the reviewer for this important point. To clarify our rationale for selecting differentially abundant TCRB clusters using a p-value threshold of < 0.001, we have revised the Results section to acknowledge that none of the clusters met a conventional false discovery rate (FDR) cutoff < 0.05. This is likely due to the very large number of clusters tested (~1.2 million), leading to stringent multiple testing correction. However, we note that many of the clusters identified by p-value also demonstrated moderate to high variable importance scores in the elastic net model, suggesting that they contribute meaningfully to classification performance despite conservative FDR estimates.

**Revised Results:**

*Given the large number of clusters tested, no individual cluster met a false discovery rate (FDR) threshold of < 0.05. Therefore, we used a nominal p-value threshold of < 0.001 for exploratory identification of candidate clusters. Notably, many of the clusters identified by p-value also exhibited moderate to high variable importance scores in the elastic net model, supporting their collective relevance to disease classification, despite the absence of strong univariate effects.*

7. Lines 245-247: The authors identified 111 (out of 507 clusters) “CDR3β amino acid sequences associated with T cell specificities for various microbial and human antigens or associated with various pathologic conditions”. Figure 4C should be discussed in detail in the main manuscript.

We thank the reviewer for this helpful suggestion. In response, we have expanded the corresponding section of the Results to more fully describe the findings shown in Figure 4C.

**Revised Results:**

*This search uncovered 111 CDR3β amino acid sequences previously reported to be associated with microbial (e.g., cytomegalovirus, Epstein-Barr virus, influenza, tuberculosis, SARS-CoV-2, yellow fever), autoimmune (e.g., multiple sclerosis, type 1 diabetes, celiac disease, rheumatoid arthritis), or cancer-related (e.g., melanoma, neoantigen) antigens. Figure 4C presents a circle packing plot that illustrates the hierarchical relationships among these clusters. In this visualization, outer grey circles represent individual TCRB clusters, and inner colored circles denote CDR3β sequences with known antigen specificity, colored by disease or antigen type. Notably, several TCRB clusters contained distinct CDR3β sequences that shared the same antigen specificity, such as cytomegalovirus, underscoring the method’s ability to group functionally convergent TCRs based on structural similarity. Colored circles not enclosed within grey boundaries indicate singleton clonotypes with known specificity that did not cluster with others. These annotated sequences were observed across both healthy and plasma cell dyscrasia samples, suggesting shared antigen exposures or public T-cell responses rather than disease-specific enrichment.*

8. The study does not explain why TCR clusters differ between healthy and disease groups if diversity is similar. Are these clusters tumor-reactive, by stander expansions, or noise?

We appreciate the reviewer’s insightful question regarding the biological significance of differentially abundant TCR clusters despite similar overall repertoire diversity between healthy individuals and patients with plasma cell dyscrasias. This apparent paradox likely reflects the fact that global diversity metrics such as the Gini coefficient summarize the overall shape of the repertoire (i.e., distribution of clone sizes) but do not capture qualitative differences in TCR sequence composition or antigen specificity. We now emphasize this point in the Discussion, acknowledging that while these TCRB clusters are distinct between groups and contribute to classification, their biological relevance, particularly whether they are tumor-specific or bystander, is not yet known.

**Revised Discussion:**

*The biological significance of these differentially abundant clusters remains uncertain, but several possibilities may explain their enrichment in plasma cell dyscrasias. First, these clusters could represent tumor-reactive T cells that have undergone clonal expansion in response to malignant or pre-malignant plasma cell antigens. Second, they may reflect bystander T-cell expansions driven by chronic immune activation, microbial exposure, or tissue remodeling. Third, differences in the TCRB repertoire between patients and controls could be influenced by non-disease factors such as subclinical infections or vaccination history. Additionally, they could represent public or convergent TCRs that are disproportionately represented in the disease setting due to antigen-independent selection pressures. Notably, most differentially abundant clusters did not map to known antigens, likely reflecting the limited coverage of current TCR-antigen reference databases. Nevertheless, several clusters contained distinct CDR3β sequences with known specificity for the same antigen, suggesting potential convergent immune responses.*

9. Validate top clusters experimentally (e.g., TCR transfection + antigen screening).
We agree with the reviewer that experimental validation of the identified TCRB clusters is essential to determine their functional relevance and antigen specificity. However, we respectfully note that the primary aim of this study was to computationally profile the TCR repertoire and identify sequence-level features and shared motifs that distinguish plasma cell dyscrasias from healthy individuals. The scope of our current work is limited to sequence-based analyses and in silico predictions; experimental determination of antigen specificity is beyond the technical capabilities of our current study. That said, our findings provide a prioritized list of candidate clusters, many of which contain CDR3β sequences with known antigen associations, that serve as a valuable foundation for future mechanistic and functional validation studies. These points are mentioned in the Discussion section.

**Reviewer #2:** The manuscript presents an extensive T-cell–receptor (TCR) repertoire analysis across the plasma-cell-dyscrasia continuum and introduces a convergent-clustering with machine-learning (ML) pipeline that reportedly distinguishes patients from healthy donors. The topic is important and, in principle, within PLOS ONE’s scope. However, several methodological and interpretative weaknesses currently prevent me from recommending acceptance. Addressing the issues below should be feasible without re-designing the study, but will require substantial re-analysis and clarification.

1. Differential-abundance screening uses a two-sided Fisher test with α = 0.001 but no FDR control. With 1.24 M tests, ~1 240 false-positive clusters are expected by chance. Re-run the analysis with FDR correction and report the number of clusters that remain significant. Retrain the ML models with this revised feature set.

We thank the reviewer for this important point. As noted in our response to Reviewer 1, Comment 6, false discovery rate (FDR) correction was performed and is reported in Supplemental Table 3 for all 507 clusters identified using a nominal p-value threshold of < 0.001. However, due to the large number of comparisons (~1.2 million), none of the clusters remained significant at an FDR < 0.05.

The fact that no individual TCRB cluster passed FDR correction highlights a key challenge in high-dimensional immune repertoire data: subtle, distributed signals often fail traditional univariate tests but can be collectively informative when analyzed using multivariate models. Our machine learning models effectively integrated information across the 507 selected clusters, achieving robust classification performance. Moreover, several clusters showed moderate to high variable importance, indicating that they contributed meaningfully to the models’ ability to distinguish between healthy individuals and those with plasma cell dyscrasias.

**Revised Results:**

*Given the large number of clusters tested, no individual cluster met a false discovery rate (FDR) threshold of < 0.05. Therefore, we used a nominal p-value threshold of < 0.001 for exploratory identification of candidate clusters. Notably, many of the clusters identified by p-value also exhibited high variable importance scores in the neural network model, supporting their collective relevance to disease classification, despite the absence of strong univariate effects.*

2. AUROC = 0.84 is based on a single 70/30 random split drawn from the same sequencing batches. No confidence interval (CI) is provided; external generalisability is unknown.

Please: (a) Implement repeated stratified hold-outs (e.g., 5 × 20 %) or provide an independent validation cohort. (b) Report AUROC, accuracy, sensitivity, specificity, and Kappa with 95 % CIs. (c) Compare the best model to the others with a statistical test (e.g., DeLong).

We thank the reviewer for their insightful comment. In response, we implemented a two-stage validation framework. First, we used repeated stratified 80/20 splits within the training dataset (70% of samples) to assess model robustness and report mean performance metrics with 95% confidence intervals. Second, we evaluated the final models on an independent 30% test set that was not used during feature selection or training to assess generalizability. We also compared classifiers using DeLong’s test. These changes address concerns regarding confidence intervals, overfitting, and external validation.These findings are reflected in the updated manuscript text, new Supplemental Table 4, and revised Figure 3C.

**Revised Methods:**
*We then assessed the predictive utility of the selected TCRB clusters using six machine learning algorithms: elastic net regularization using glmnet (v4.1-8), random forest (randomForest, v4.7-1.2), support vector machine with radial basis function kernel (kernlab, v0.9-33, via svmRadial in caret), neural network (nnet, v7.3-19), gradient boosting machine (gbm, v2.2.2), and k-nearest neighbors (class, v7.3-22) (code available in Supplemental Methods). A two-stage evaluation framework was implemented to measure model performance. First, we conducted internal validation using a repeated stratified hold-out procedure with five iterations of random 80/20 train/test splits within the training set. For each model and iteration, we calculated the area under the receiver operating characteristic curve (AUROC), accuracy, sensitivity, specificity, and Cohen’s Kappa statistic. Performance metrics were summarized across repeats using the mean and empirical 95% confidence intervals. Pairwise DeLong tests were used to compare AUROC values between models. To assess generalizability, each model was retrained on the full training dataset and evaluated on the held-out 30% test set, which was not used during feature selection or training. External performance metrics were reported separately. Variable importance scores were computed using the varImp function in the caret package, and recursive feature elimination was performed using rfe with 10-fold cross-validation to identify the most informative TCRB clusters.*

**Revised Results:**
*To validate the discriminative ability of TCR sequence-based features, we employed a two-stage machine learning framework incorporating both internal and external validation. First, we trained six machine learning classifiers on 70% of the samples using repeated stratified hold-out resampling (5 x 80/20 splits) to estimate performance variability. For each model, we calculated the mean AUROC, accuracy, sensitivity, specificity, and Cohen’s Kappa, along with 95% confidence intervals. The random forest, elastic net, and support vector machine models consistently achieved the highest average performance (mean AUROCs ≥ 0.98), while the gradient boosting machine, neural network, and k-nearest neighbors classifiers performed less well (Figure 3C). Pairwise DeLong tests indicated no statistically significant AUROC differences between the top three models, though elastic net outperformed the neural network and gradient boosting machine in several resampling iterations (Supplementary Table 4).*

*To evaluate generalizability, we retrained each model on the full 70% training set and assessed performance on a held-out 30% test set that had not been used during model development, feature selection, or tuning. The random forest classifier achieved the best external performance, with an AUROC of 0.859, accuracy of 0.814, sensitivity of 0.796, specificity of 0.833, and Kappa of 0.629 (Figure 3D). These results confirm that disease-associated TCRB clusters encode sufficient discriminatory signal to distinguish patients with plasma cell dyscrasias from healthy individuals, even when tested on unseen data.*

3. Provide an enrichment analysis comparing antigen types in disease-enriched vs healthy-enriched clusters. If no significant bias emerges, tone down the claim that the 111/507 clusters containing database-matched CDR3βs “likely reflect differential antigen recognition.”

We appreciate the reviewer’s suggestion. We performed an enrichment analysis of known antigen-specific CDR3βs. We did not identify a significant enrichment of specific antigen types among clusters that were more frequent in plasma cell dyscrasia samples compared to healthy controls. These annotated sequences were observed across both healthy and plasma cell dyscrasia samples, suggesting shared antigen exposures or public T-cell responses rather than disease-specific enrichment. We have revised the Results section to clarify this point with the following statement: *These annotated sequences were observed across both healthy and plasma cell dyscrasia samples, suggesting shared antigen exposures or public T-cell responses rather than disease-specific enrichment.*

4. Except for the R packages and their versions, please also clarify the executable workflow or script directory with hyperparameters.

We thank the reviewer for this suggestion. In the revised manuscript, we have added a new section to the Supplemental Methods that includes the full R code used to train and evaluate the machine learning models. This includes the model names, hyperparameters (via default settings), train/test split procedure, and performance metrics. We have also clarified the R package versions used in the analysis within the methods section.

**Reviewer #3:** Major Comments and Suggestions:
1. The authors concluded that TCR repertoire diversity did not correlate with disease stage or response to treatment. While this is clearly supported by the multivariate models, the clinical interpretation of this finding is underdeveloped. Please elaborate on the implications of these null results for understanding immune surveillance and disease progression.

We appreciate the reviewer’s point and have expanded the discussion in the revised manuscript to better contextualize the clinical interpretation of these null results.

**Revised Discussion:**

*The absence of a correlation between global TCRB diversity and disease stage or treatment outcomes implies that overall diversity may be an insufficient marker of effective immune surveillance in plasma cell dyscrasias. These findings suggest that failure of immune control may be driven more by qualitative features of the T-cell response, such as antigen specificity, clonal expansion of disease-relevant T cells, or functional exhaustion, rather than by the total number or evenness of distinct TCRs. It is also possible that disease-associated immune changes are more pronounced within specific tissue compartments or T-cell subsets that are not captured by bulk peripheral repertoire metrics.*

2. The machine learning framework was well-structured, but critical details need to be clarified, such as how feature selection was handled before model training? What measures were taken to prevent data leakage between training and test sets? Was age included as a covariate in the model, or controlled for via stratification?

We thank the reviewer for their thoughtful questions regarding our machine learning framework. The R code used for model training, evaluation, and performance comparison, including cross-validation, metric calculation, and ROC analysis, is provided in the Supplemental Methods to ensure transparency and reproducibility.

1. **Feature Selection**: All features (i.e., differentially abundant TCRB clusters) used for model training were pre-specified based on an independent cohort. No feature selection was performed using the test data, ensuring no information leakage. This approach preserved the integrity of the evaluation by fixing the feature set prior to any model training or testing.
2. **Data Leakage Prevention**: To prevent data leakage, model training and evaluation were conducted using repeated stratified train/test splits. All modeling steps—including training, prediction, and performance assessment—were performed independently within each iteration. The test set was held out during model fitting and used only for final evaluation.
3. **Age as a Covariate**: Age was not included as a covariate in the classification models. However, we accounted for potential confounding by age in upstream analyses of TCRB diversity by using multivariable models adjusted for age. Additionally, we confirmed that the age distribution within differentially abundant clusters did not differ significantly from the broader healthy donor cohort (Supplemental Figure 6), suggesting minimal age-driven bias in cluster-level associations.

3. Although the ROC AUC of 0.84 was promising, a confusion matrix or metrics such as sensitivity/specificity should be included for interpretability.

Thank you for this helpful suggestion. In response, we have updated Figure 3C and added a new Supplemental Table 4 to report detailed performance metrics for all models. These now include sensitivity, specificity, accuracy, AUROC, and Cohen’s Kappa, averaged across repeated train/test splits with 95% confidence intervals. This additional information improves the interpretability of model performance and allows direct comparison across classifiers.

4. While amino acid properties and antigen database searches were performed, the biological implications remain speculative. The authors stated that distinct clusters differ between patients and healthy controls, but most of these did not map to known antigens. The authors could consider discussing potential causes of this divergence (e.g., subclinical infections, age, vaccination history).

Thank you for this thoughtful observation. We agree that the biological implications of the differentially abundant TCRB clusters are largely speculative in the absence of known antigen specificity. As suggested, we have expanded the Discussion to acknowledge potential explanations for the observed divergence beyond disease-related antigen recognition.

**Revised Discussion:**

*The biological significance of these differentially abundant clusters remains uncertain, but several possibilities may explain their enrichment in plasma cell dyscrasias. First, these clusters could represent tumor-reactive T cells that have undergone clonal expansion in response to malignant or pre-malignant plasma cell antigens. Second, they may reflect bystander T-cell expansions driven by chronic immune activation, microbial exposure, or tissue remodeling. Third, differences in the TCRB repertoire between patients and controls could be influenced by non-disease factors such as subclinical infections or vaccination history. Additionally, they could represent public or convergent TCRs that are disproportionately represented in the disease setting due to antigen-independent selection pressures. Notably, most differentially abundant clusters did not map to known antigens, likely reflecting the limited coverage of current TCR-antigen reference databases. Nevertheless, several clusters contained distinct CDR3β sequences with known specificity for the same antigen, suggesting potential convergent immune responses.*

5. Age was accounted for in diversity analyses via multivariate regression and age-exclusion criteria. However, it’s unclear whether machine learning and clustering analyses were fully adjusted for age, which is a known confounder in TCR studies. Please clarify this point or conduct an age-matched subgroup analysis.

We appreciate the reviewer’s attention to the potential confounding effects of age. Age was not included as a covariate in the machine learning classification models. However, we accounted for age in upstream analyses of TCRB diversity using multivariable regression models adjusted for age, and we applied exclusion criteria to remove younger healthy donors from comparative analyses. To further assess whether age influenced the machine learning findings, we examined the age distribution within the differentially abundant TCRB clusters and found no significant deviation compared to the broader healthy donor cohort (Supplemental Figure 6). These results suggest minimal age-driven bias in the identified cluster-level associations.
